# Leveraging Glycan–Glycan Interactions to Tune the Conformation of Glycan Hairpins and Build Rigid 3D Architectures

**DOI:** 10.1021/jacs.6c04539

**Published:** 2026-04-21

**Authors:** Nishu Yadav, Yadiel Vázquez-Mena, Ana Poveda, Dominik Weh, Jesús Jiménez-Barbero, Yu Ogawa, Martina Delbianco

**Affiliations:** 1 Department of Biomolecular Systems, 28321 Max Planck Institute of Colloids and Interfaces, Am Mühlenberg 1, Potsdam 14476, Germany; 2 Department of Chemistry and Biochemistry, Freie Universität Berlin, Arnimallee 22, Berlin 14195, Germany; 3 CICbioGUNE, Basque Research and Technology Alliance, Derio 48160, Spain; 4 Ikerbasque, Basque Foundation for Science, Bilbao 48009, Spain; 5 Department of Inorganic & Organic Chemistry, Faculty of Science and Technology, University of the Basque Country, EHU-UPV, Leioa 48940, Spain; 6 Centro de Investigación Biomedica En Red de Enfermedades Respiratorias, Madrid 28029, Spain; 7 CERMAV, Univ. Grenoble Alpes, CNRS, Grenoble 38000, France; 8 Department of Sustainable and Bioinspired Materials, Max Planck Institute of Colloids and Interfaces, Am Mühlenberg 1, Potsdam 14476, Germany

## Abstract

Glycan folding and aggregation remain poorly understood despite their essential roles in structural and biological functions. We present a reductionist approach leveraging a glycan sequence that spontaneously folds into a hairpin conformation, enabling the systematic analysis of glycan intra- and intermolecular interactions. Our modular glycan hairpin design facilitated the incorporation of various oligomers representative of naturally occurring linear polysaccharides as hairpin strands, allowing us to investigate the effects of glycan–glycan interactions (i.e., strand–strand interactions). An initial screening based on molecular dynamics simulations revealed that strand composition strongly influences conformational stability, with hairpins ranging from highly flexible to exceptionally rigid structures. Selected rigid hairpin models, based on chitin strands, were synthesized and characterized in aqueous solution by using nuclear magnetic resonance spectroscopy and small-angle X-ray scattering, providing direct experimental validation of intramolecular interactions that stabilize folding. These highly rigid secondary structures assembled into micrometer-long platelet-like architectures that were characterized with transmission electron microscopy and electron diffraction to reveal details of intermolecular interactions driving supramolecular aggregation. These findings demonstrate that hairpins are modular systems to explore molecular details of glycan–glycan interactions as well as useful building blocks to craft glycan-based architectures.

## Graphical abstract

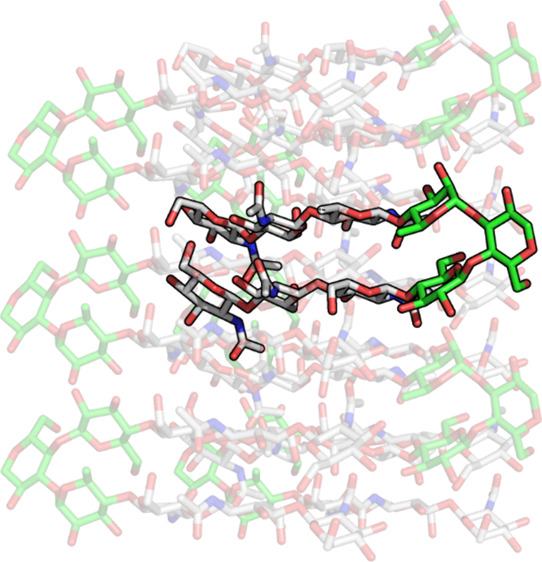

## Introduction

Self-folding oligomers are valuable soluble models for understanding the fundamental rules governing biopolymer folding and aggregation in nature.^1^ For instance, peptide hairpins revealed molecular interactions that regulate protein folding,^2–4^ recognition,^5^ and aggregation.^6^ These short structural motifs resemble the β-sheet conformation found in natural proteins, making them ideal systems for studying folding pathways and stability factors.^7^ Such reductionist approach allowed dissecting how interactions between different side chains, hydrogen bonding, hydrophobic effects, electrostatic interactions, and steric constraints contribute to protein folding.^8–13^ This understanding also offered design principles for crafting bioinspired supramolecular materials.^14^

While much attention has been given to the design of folding peptides, this strategy has hardly been applied to study glycans, the most abundant biomaterial on earth.^15^ Glycans can engage in a multitude of noncovalent interactions such as hydrogen bonding, van der Waals, and electrostatic interactions to form crystalline materials.^16^ For example, the cellulose fibrils in plant cell walls are held together by extensive hydrogen bonding networks between glucose chains and with major contributions from London dispersion forces, giving them tensile strength.^16–18^ Similarly, chitin exploits hydrogen bonding and van der Waals forces to form strong, rigid structures that provide support in the exoskeletons of arthropods and the cell walls of fungi.^19^ These interactions are also critical to their biological functions,^20^ including molecular recognition,^21,22^ energy storage,^23,24^ and structural support.^25^

Minimalistic, self-folding oligosaccharides could serve as models of natural polysaccharides, revealing molecular aspects of their conformation and aggregation. In turn, this knowledge could be used to tune glycan secondary structures and to craft novel glycan-based architectures.

Recently, we presented a glycan sequence capable of spontaneously folding into a hairpin conformation.^28^ The design features a trisaccharide turn unit substituted with two cellulose-stacking strands. Systematic NMR analysis unequivocally confirmed its folded conformation in aqueous solution and revealed the stabilizing effect of multiple strand–strand interactions.^29^ The modular design of our glycan hairpin allows us to replace the cellulose strands with other glycan sequences, offering an ideal model system to systematically investigate glycan–glycan interactions between two single glycan chains (i.e., interstrand interactions).

Herein, we implemented different linear polysaccharides found in nature (as well as combinations of those) as hairpin strands to analyze their ability to engage in glycan–glycan interactions. Using molecular dynamics (MD) simulations, we systematically screened seven distinct hairpins with varying glycan strands. The screening revealed that some polysaccharide combinations decrease the conformational stability of our glycan hairpin while others resulted in highly rigid hairpin structures. These models were synthesized and examined in aqueous solution using nuclear magnetic resonance (NMR) and small-angle X-ray scattering (SAXS) and in the solid phase using transmission electron microscopy (TEM) and electron diffraction (ED), revealing molecular details of glycan–glycan interactions. These forces, which mimic the organization of natural polysaccharides, were leveraged to craft exceptionally rigid secondary structures and assemble supramolecular materials from short oligosaccharides.

## Results and Discussion

All hairpins discussed in this work are based on the same trisaccharide turn, consisting of a reducing Glc branching unit, substituted with a β-Glc unit at C-4 and a α-Rha unit at C-3. A nonconventional C–H···O hydrogen bond between the H-5 of L-Rha and the O-5 of Glc stabilizes the turn, forming a 10-membered ring that holds the two branches in an ideal parallel orientation.^28^ As strands, we screened oligomers representative of natural crystalline polysaccharides (Figure 1), such as cellulose (1,4-β glucose),^30^ chitin (1,4-β *N*-acetyl glucosamine),^31^ and xylan (1,4-β xylose),^32^ as well as combinations of those. By examining how different polysaccharide strands affect the conformational stability of our glycan hairpin, we create a reductionist approach to examine glycan–glycan interactions between two isolated glycan chains.

**Figure 1:**
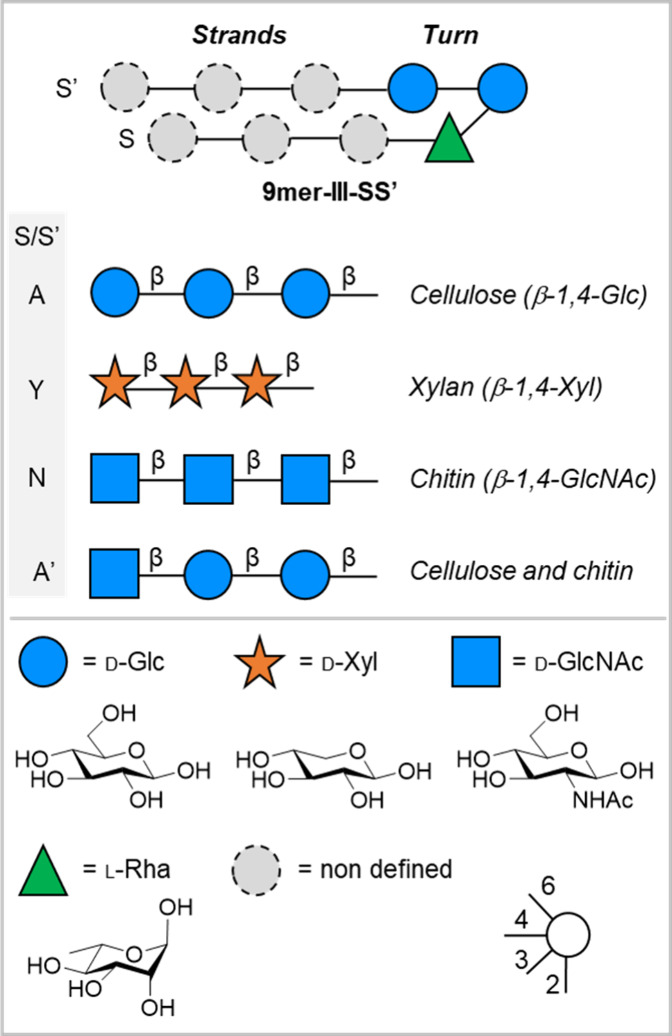
A model glycan hairpin modified with strands representative of various linear polysaccharides found in nature to investigate the effects of strand–strand interactions on glycan folding. Each strand is named as follows: cellulose is labeled as A, xylan as Y, chitin as N, and the combination of cellulose and chitin as A’.^26^ The following abbreviations are used for monosaccharides: Glc = glucose (blue circle), Xyl = xylose (orange star), GlcNAc = *N*-acetyl glucosamine (blue square), and Rha = rhamnose (green triangle). The monosaccharide residues are represented following the Symbol Nomenclature for Glycans (SNFG).^27^

Each hairpin name describes the length, type of turn unit, and modifications, providing direct information about the key features of the glycan.^33^ The strands are named as follows: **9mer-III-SS’**, where S represents the bottom strand and S’ represents the top strand. In this nomenclature, S/S’ refer to strands with different polysaccharides; cellulose is labeled as A, xylan as Y, chitin as N, and the combination of chitin and cellulose as A’ (Figure 1). Monosaccharide residues within a hairpin are labeled with letters from the reducing to the nonreducing end, prioritizing C-3 linked residues over C-4 linked residues. Proton labeling in a monosaccharide follows this format: for example, the proton at C-1 of Rha-B is named “Rha B-1” (Figure S01). NMR signals of residues at the reducing end are additionally labeled with α or β.

### Initial Screening with Molecular Dynamic Simulations

We designed seven distinct hairpin structures, in which we systematically varied the strand type. Our collection aimed to analyze three key aspects: (1) the effect of the same oligosaccharides on both strands (e.g., Y and Y); (2) the interactions between different polysaccharides (e.g., A and Y); and (3) the effect of single variations within the strands (e.g., A’ and A’).

Atomistic MD simulations were carried out for all designed glycan hairpins to analyze their conformational preference and flexibility (see Supporting Information Section 1). All the modeled structures were simulated for 500 ns, employing a modified version of the GLYCAM06 ^34^ carbohydrate force field. The systems were solvated with the TIP5P^35^ water model to avoid excessive interactions among the monomers.^36^

**9mer-III-AA** (as reference),^29^ **9mer-III-YY**, and **9mer-III-NN** feature strands based on oligomers representative of cellulose, xylan, and chitin, respectively. The radius of gyration (RoG) and root-mean-square deviation (RMSD) indicated significantly less conformational freedom for **9mer-III-NN** in comparison to **9mer-III-AA** (Figure 2a and Figure S02). On the other hand, **9mer-III-YY** showed a broader RoG distribution and fluctuations in the RMSD, suggesting its higher flexibility (Figure 2a and Figure S02). Overall, these data suggest weaker interactions between xylan strands compared to cellulose and chitin.

**Figure 2:**
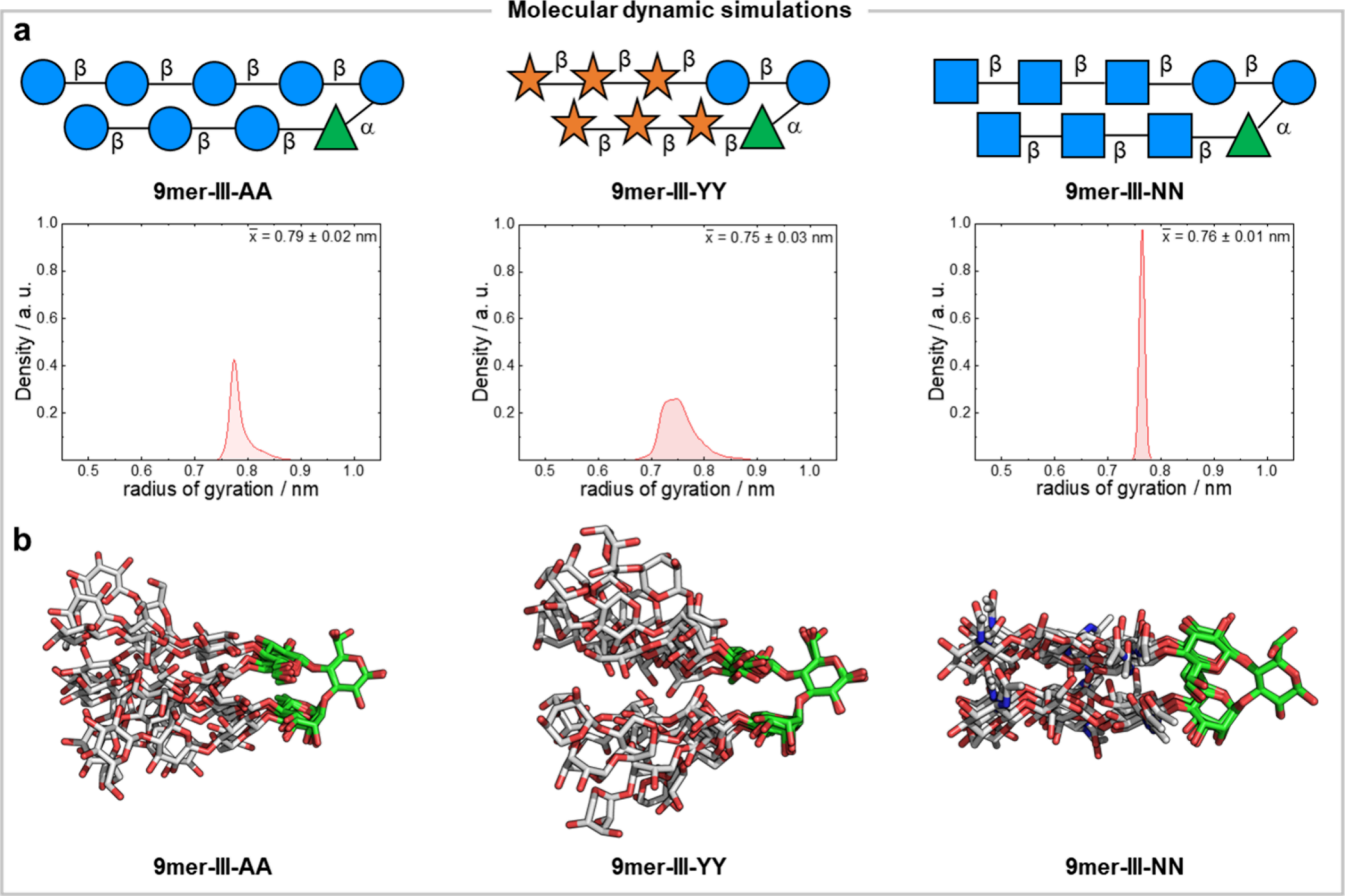
(a) The SNFG representation of **9mer-III-AA, 9mer-III-YY**, and **9mer-III-NN** and their radius of gyration (RoG) plots showing different trends in terms of overall conformation and flexibility. (b) Superimposition of four representative snapshots, each depicting one cluster from the four major clusters identified for **9mer-III-AA**, **9mer-III-YY**, and **9mer-III-NN** using the glycan analysis pipeline (GAP).

The analysis of the dihedral angles Φ (Phi) and Ψ (Psi) of the turn unit revealed the same trend for all three hairpins (Figure S03), with both glycosidic bonds adopting a major preferred conformation. When we compared the Ramachandran plots for the dihedral angles of the strands, we observed less conformational freedom in the glycosidic linkages of **9mer-III-NN** than in **9mer-III-AA** (Figures S04 and S05). **9mer-III-YY** showed a broader distribution of glycosidic dihedrals (Figure S06). The inter-residue distances confirmed the closer conformation for **9mer-III-NN** compared to **9mer-III-AA**, while **9mer-III-YY** appears fully open (Figure S07).

We utilized the glycan analysis pipeline (GAP)^37^ to extract representative 3D structures along with their frequencies in the MD simulation. GAP allowed us to determine the number of representative conformational clusters that described our molecules. For each cluster, we obtained a representative 3D structure through a kernel density estimation (KDE) analysis. This analysis revealed that **9mer-III-NN** adopts a closed hairpin conformation across all clusters. **9mer-III-AA** is predominantly closed in about 80% of the simulation time but shows some flexibility in the remaining 20%, resulting in the presence of some open conformers. **9mer-III-YY** adopts an open conformation in all clusters (Figure 2b and Supporting Information Section 1.3). A closer look at the 3D structure of **9mer-III-NN** indicated a network of hydrogen bonds between the amidic oxygens of one strand and the *N*-hydrogens on the opposite strand that may contribute to its higher conformational rigidity (Figure S08).

Next, we investigated how the interactions between different polysaccharide strands affect hairpin stability. Such hybrid systems are common in nature; for example, cellulose–xylan interactions play important roles in plant secondary cell walls, where xylan binds cellulose microfibrils via noncovalent interactions and contributes to wall integrity and mechanics.^38,39^ We studied **9mer-III-YA**, a hybrid structure carrying strands representative of xylan and cellulose (Figure S03), to verify if cellulose–xylan interactions could be observed using our model system. The slightly sharper RoG and closer interstrand distances in **9mer-III-YA** compared to **9mer-III-YY** suggested that cellulose–xylan interactions might be beneficial for the hairpin conformational stability. However, despite this minor stabilization, **9mer-III-YA** still appears quite flexible compared to **9mer-III-AA** (Figure S02). Similarly, hybrid systems based on cellulose and chitin strands, **9mer-III-AN** and **9mer-III-NA** (Figure S03), remained relatively flexible (Figure S02), oscillating between closed and open conformers, probably due to differences in strand sizes (N vs A).

As the analysis of **9mer-III-NN** highlighted the importance of the H-bond between the amido groups for conformational stabilization, we designed an additional hairpin in which we replaced the nonreducing end Glc residues in **9mer-III-AA** with GlcNAc (**9mer-III-A’A’**) (Figure S09). This variation aimed to investigate whether this interstrand H-bonding could be exploited as a “noncovalent staple” to further stabilize other hairpins. Indeed, we observed a slight stabilization, with sharper RoG for **9mer-III-A’A’** (Figure S09) when compared to **9mer-III-AA**, suggesting that a careful positioning of amido-containing monosaccharides could be a strategy to stabilize folded glycans.

Overall, this computational screening suggested that chitin–chitin interactions are particularly suited to access extremely rigid glycan hairpins, offering the possibility of extra interactions between amido groups. This rigidity arises from the orientation of the two strands, with amido groups pointing toward each other, creating a “zip-like” structure.^40^ A comparison with crystallographic models of α- and β-chitin^41^ (Figure 3 and Figure S23) indicated that the two strands of **9mer-III-NN** (in aqueous solution) are in a similar distance range and orientation to those observed in the crystal structure of natural chitins (Figure S23). This comparison underscores that **9mer-III-NN** is an ideal representation of the packing found in natural chitin, as well as a promising candidate for the creation of bioinspired glycan foldamers and assemblies. We therefore proceeded with the synthesis and structural analysis of chitin-based hairpins.

**Figure 3:**
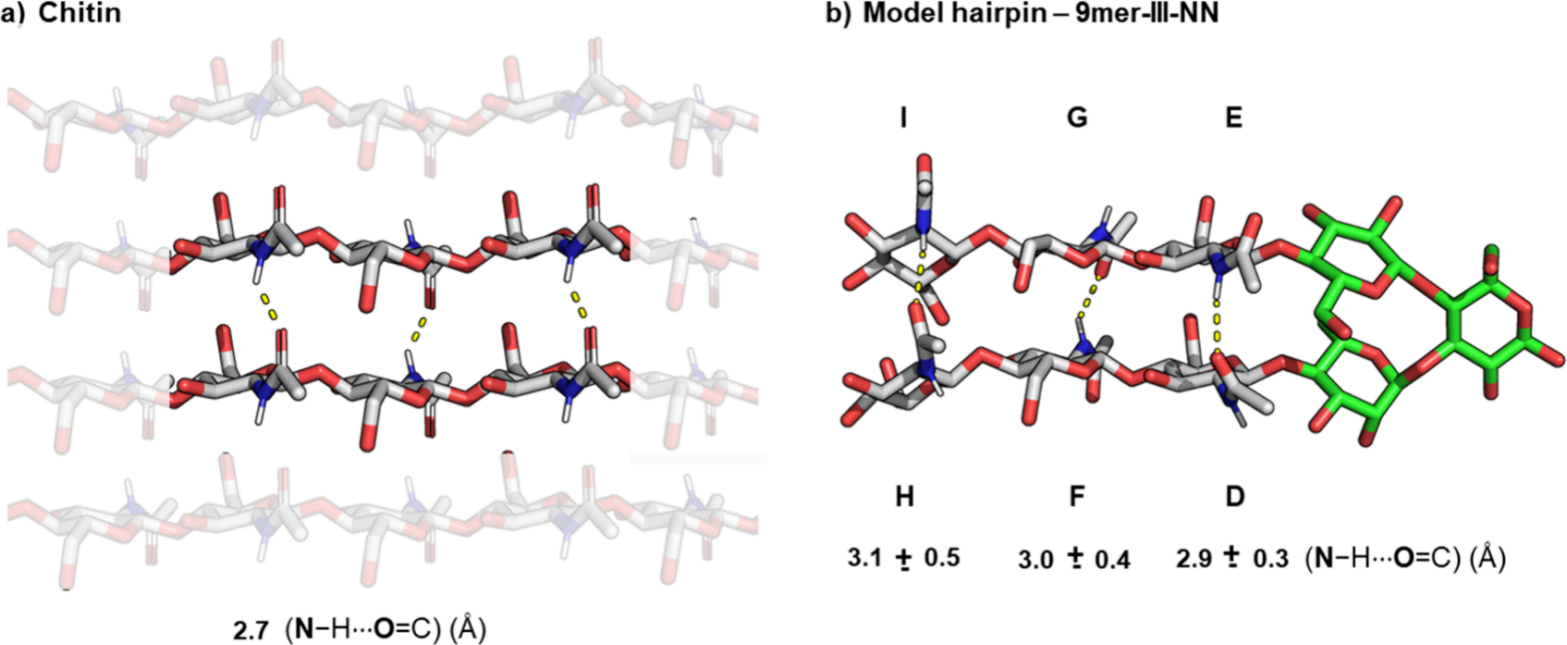
(a) Chitin model showing the interchain H-bond distance (N–H···O=C) calculated from the model of β-chitin. Further details are provided in the Supporting Information Section 1.5. (b) **9mer-III-NN** model showing interstrand H-bond average distance (N–H···O=C) calculated form the overall MD simulation.

### Synthesis and NMR Analysis

We targeted two chitin-based hairpins, **5mer-III-NN** and **9mer-III-NN** (Figure 4a). **5mer-III-NN** is a shorter version of **9mer-III-NN** carrying one GlcNAc unit at each strand, engaging in a single N–H···O=C bond (Figures S19–S22). This molecule was selected to simplify the structural characterization before moving on to the more complex **9mer-III-NN.**

**Figure 4:**
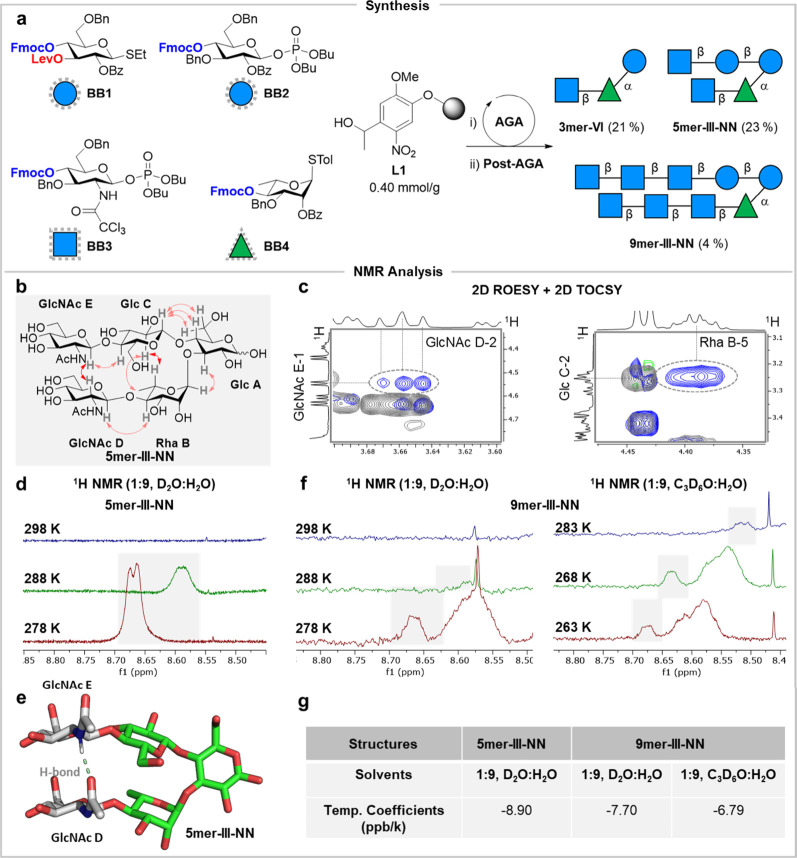
(a) The glycan hairpin model structures were prepared by AGA using protected monosaccharide BBs. Overall yields are reported in parentheses. Reaction conditions for AGA and post-AGA are reported in Supporting Information Section 2. (b) Experimental NOEs extracted from ROESY NMR experiments for **5mer-III-NN** (red arrows). (c) Excerpt of the 2D ROESY NMR of **5mer-III-NN** showing the key NOE between the residues of the turn and the strands of the hairpin (293 K, mixing time 300 ms, D_2_O, and 700 MHz; for full spectra, see Figure S29). (d) ^1^H NMR spectra of **5mer-III-NN** at different temperatures in D_2_O/H_2_O (1:9) and 800 MHz showing a sharp N–H peak belonging to the GlcNAc E residue arising at 278 K (for TOCSY analysis, see Figure S34). (e) Snapshot obtained from the MD simulation of **5mer-III-NN** showing a hairpin conformation with H-bonding indicated with the dotted line. (f) ^1^H NMR spectra of **9mer-III-NN** at different temperatures in D_2_O/H_2_O (1:9) (left) and H_2_O/C_3_D_6_O (1:9) (right) and 800 MHz showing an N–H peak appearing at lower temperatures (Figures S35–S39) (g) Temperature coefficient comparison of **5mer-III-NN** and **9mer-III-NN** (see Supporting Information Section 4.4).

Both hairpins were synthesized by AGA on a solid support (see Supporting Information Section 3.5) in an overnight run using previously reported conditions.^28,42^ Post-AGA manipulations included solid-phase methanolysis, photocleavage from the solid support, and hydrogenolysis. A single purification step afforded the target hairpin analogues in overall yields of 23% (**5mer-III-NN**) and 4% (**9mer-III-NN**). The relatively low yield for **9mer-III-NN** was ascribed to its poor solubility after global deprotection, causing precipitation already at a 1 mg/mL concentration. In the future, this issue could be attenuated by exploiting phosphates as traceless solubilizing groups.^26,43^ Additionally, we synthesized **3mer-VI** (21% overall yield), a control trisaccharide lacking the nonconventional H-bond, as reference.

Conformational analysis of **5mer-III-NN** was conducted using nuclear magnetic resonance (NMR) spectroscopy.^44,45^ The chemical shift deviation (Δδ) of Rha B-5 served as experimental marker of the turn closed conformation.^46,47^ The observed downfield shift of Rha B-5 in **5mer-III-NN** when compared to **3mer-VI** (Δδ = 0.39 ppm) confirmed the presence of the nonconventional hydrogen bond between Rha B-5 and the ring oxygen of Glc C (see Supporting Information Section 4.1 (Figure S32)). Chemical shift differences between GlcNAc E-1 (pointing to the interior of the hairpin) and GlcNAc D-1 (pointing to the exterior) indicated that hydrophobic contacts between CH-rich sugar faces further stabilized the folded state (Table S2).^33^ To confirm the spatial proximity between the key residues at both sides of the putative hairpin, ROESY experiments^45^ were employed. Key inter-residue NOEs between Glc C-2/Rha B-5 and GlcNAc E-1/GlcNAc D-2 indicated the closed conformation of the turn motif and the proximity of the two strands (Figure 4b,c and Supporting Information Section 4.1). Short inter-residue distances of 2.5 Å (Glc C-2/Rha B-5) and 2.9 Å (GlcNAc E-1/GlcNAc D-2), estimated by applying the isolated spin pair approximation to the NOE intensities, confirmed the closed conformation of **5mer-III-NN** (see Supporting Information Section 4.1.1 or Figure S33).

^1^H NMR experiments were conducted in D_2_O/H_2_O (1:9) solution across a temperature range of 298 to 278 K to validate the presence of the H-bond between the amido groups on both strands in **5mer-III-NN** (Figure 4d,e). At 298 K, no N–H peak was detected in the low-field region. However, when the temperature was lowered to 288 K, a broader peak appeared at 8.6 ppm, with its intensity increasing further upon additional cooling to 278 K (Figure 4d and Figure S34). Selective irradiation of the N–H proton (highlighted in light gray) in a TOCSY experiment confirmed that this signal corresponds to the GlcNAc E residue (highlighted in light yellow) (Figure S34). Overall, the NMR results obtained for **5mer-III-NN** agreed with the MD simulation results, showing strong inter-residue NOEs and confirming the presence of the H-bond between the N–H and the O of the amido groups of the two GlcNAc units on opposite strands.

Next, we investigated the presence of the interstrand H-bonds between the amido groups in **9mer-III-NN** (Figure 4f and see Supporting Information Section 4.3). ^1^H NMR experiments in D_2_O/H_2_O (1:9) solution over a temperature range from 298 to 278 K revealed the emergence of N–H peaks in the downfield region. At 288 K, a small signal appeared; upon further cooling to 278 K, the peak intensity increased, and additional signals appeared between 8.52 and 8.70 ppm (Figure 4f, left and Figure S35), suggesting the presence of interstrand hydrogen bonding between the amido groups. Additional NMR experiments in C_3_D_6_O/H_2_O (1:9) solution enabled measurements at lower temperatures (down to 263 K), improving the signal resolution (Figure 4f, right and Figure S36). At 263 K, one N–H signal appeared significantly deshielded (highlighted with a light gray box) compared to the other five N–H signals (Figure 4f, right and Figure S36), suggesting that this particular N–H is involved in a more stable hydrogen bond. Overlaid 2D NOESY spectra of **5mer-III-NN** and **9mer-III-NN** confirmed that the deshielded N–H proton belongs to the GlcNAc E residue (Figures S23c and S39), as predicted by MD.

Temperature coefficients^48^ for the amidic N–H protons were calculated to assess hydrogen bond stability and solvent exposure in both molecules (Figure 4g and Supporting Information Section 4.4). In C_3_D_6_O/H_2_O, the less negative coefficient for **9mer-III-NN** suggested that its N–H groups are more protected and likely engaged in a stable hydrogen bond, potentially due to reduced water competition in the mixed solvent. In contrast, a more negative coefficient for **9mer-III-NN** in D_2_O/H_2_O indicated an increased temperature sensitivity, reflecting greater exposure to bulk water and weaker or more dynamic hydrogen bonds. The coefficient calculated for **5mer-III-NN** was the most negative among the three values (−8.90 ppb/K in D_2_O/H_2_O), indicating that its N–H group is more solvent-exposed and forms less stable hydrogen bonds (Figure 4g).This result confirms the greater structural stability of the hairpin with longer strands (**9mer-III-NN**) compared to shorter strands (**5mer-III-NN**) (Figure S23c).^33^

Overall, these results confirmed the presence of interstrand H-bonds, stabilizing the hairpin conformation. Solvent composition and oligomer length influenced the strength of these hydrogen bonds. Specifically, C_3_D_6_O promoted slightly more stable interactions in **9mer-III-NN**, whereas **5mer-III-NN** exhibited greater temperature sensitivity and weaker hydrogen bonding in aqueous environments.

### SAXS Analysis

Small-angle X-ray scattering (SAXS) offers a complementary approach to NMR for glycan structural analysis,^49^ providing a direct, low-resolution shape of macromolecules.

SAXS profiles of solutions of **9mer-III-NN** in water at concentrations of 0.25% and 1.0% w/v were recorded (Figure 5 and SI Section 5). At a lower concentration (0.25% w/v), the SAXS intensity curve was characterized by a pristine tendency along the *y* axis at the low-q region (Figure 5d,e), in agreement with a monodispersed oligosaccharide solution, in which the Guinier regime was normally observed. The radius of gyration of the dispersed glycan hairpin was analyzed from this region using the Guinier law, *I*(*q*) = *I*_0_exp­(−*q* ^2^ *Rg* ^2^/3), where *I*_0_ is the zero-angle scattering at *q* = 0, yielding a RoG of 7.6 Å (see SI Section 5). This result exactly matches the value predicted by MD simulations (RoG of 7.6 Å, Figure 2a), confirming the compact conformation of **9mer-III-NN.**

**Figure 5:**
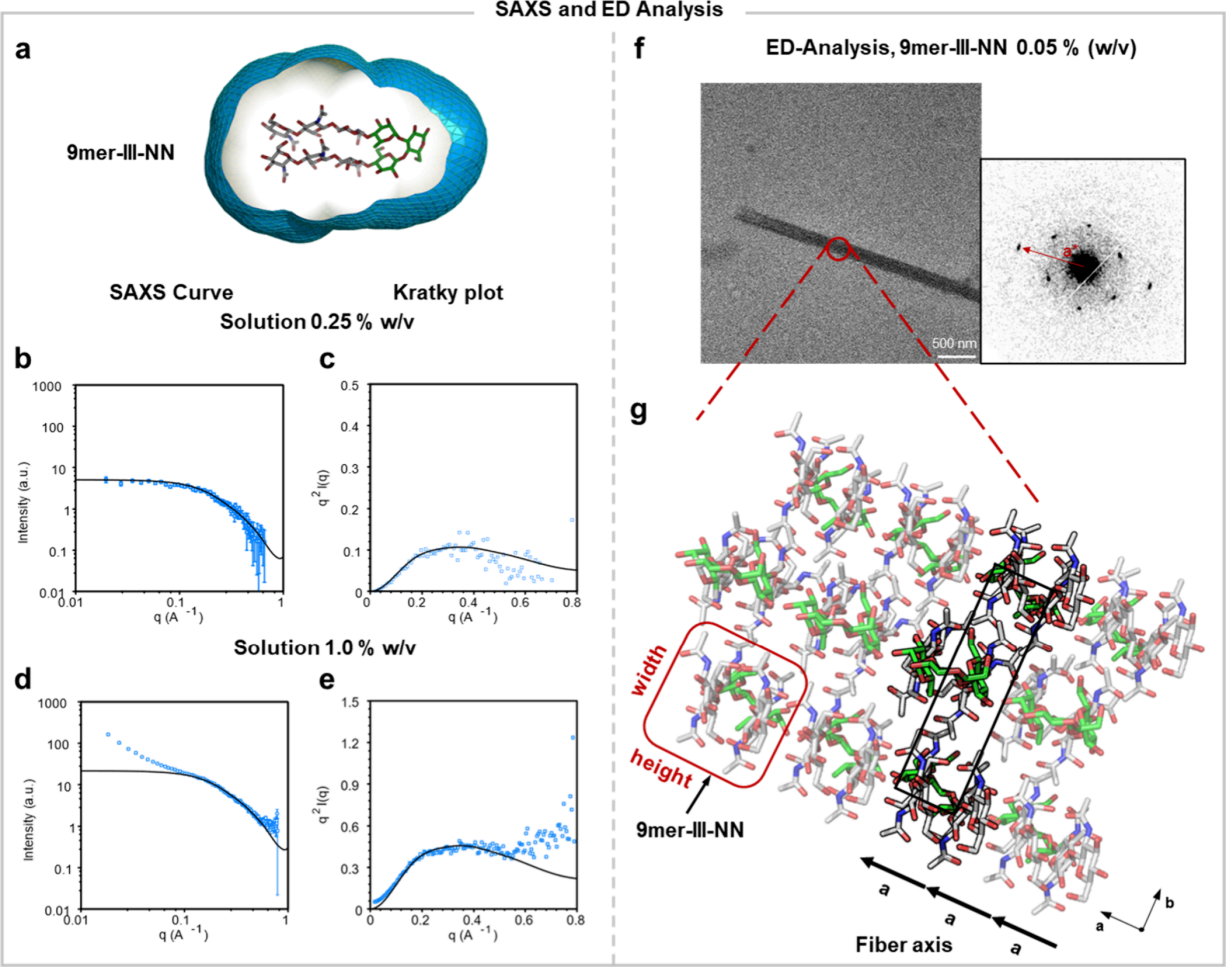
(a) Representation of the folded conformational state of **9mer-III-NN** obtained from 3 ns of MD simulations and the envelope with water molecules of the solvation layer. (b) Comparison of experimental and calculated scattering profiles at a concentration of 0.25% w/v. (c) Kratky plots at a concentration of 0.25% w/v. (d) Comparison of experimental and calculated scattering profiles at a concentration of 1.0% w/v. (e) Kratky plots at a concentration of 1.0% w/v. Experimental SAXS data are in blue with error bars from counting statistics fit, while the calculated intensity is represented with a solid line. (f) Electron micrograph (left) and electron diffraction pattern (right) of a platelet-like crystal of **9mer-III-NN**. (g) Molecular packing model of **9mer-III-NN** into a platelet-like crystal. Hairpin turns are represented in green and strands in gray, showing an antiparallel packing between adjacent molecular stacks.

The scattering curve for the higher concentration (1.0% w/v) solution exhibited two dominant features that correspond to different length scales in solution. The low-q region (*q* < 0.15 Å^–1^) showed an asymptotic growth of the scattering curve along the *y* axis, indicating the presence of large-size clusters (Figure 5b). In contrast, the high-*q* region (*q* > 0.15 Å^–1^) described the scattering of the monodisperse glycan hairpins. The presence of some aggregates in the more concentrated solution matches what was observed during the synthesis and NMR characterization of **9mer-III-NN**. Indeed, chitin is poorly soluble in water due to strong interchain hydrogen bonds between the acetyl groups.^50^

The experimental curves were compared to the simulated scattering characteristics obtained for **9mer-III-NN** following our recently developed pipeline to analyze scattering data of glycans.^51^ The predicted SAXS profile was computed from over 1000 frames taken from a 3 ns simulation using a modified version of GROMACS based on all-atom explicit-solvent MD simulations. The solvation envelope was constructed at a distance of *d* = 7 Å from the **9mer-III-NN** hairpin atoms (Figure 5a). The predicted SAXS profile of **9mer-III-NN** agrees well with all the experimental data of the 0.25% w/v solution (Figure 5d). Experimental scattering result and predicted SAXS profiles are also matching for the high-*q* regime of the 1.0% w/v solution, associated with a monodisperse glycan hairpin (Figure 5b). Calculated scattering profiles also show a clear correspondence with the experimentally determined Kratky plots of the 0.25% w/v solution (Figure 4e). Overall, SAXS analysis proved that **9mer-III-NN** adopts a compact, folded conformation, as predicted by MD simulation.

### TEM and ED Analysis

While the presence of aggregates could be detrimental to the synthesis of such compounds, it also suggests that our hairpin could offer an interesting building block to assemble supramolecular materials. Thus, we explored the aggregation tendency of the hairpin.

We crystallized **9mer-III-NN** following a hot vapor diffusion-induced crystallization protocol (see SI Section 6). TEM revealed the formation of micrometer-long platelet-like structures (Figure 5f, left). An ED pattern was obtained with the incident electron beam perpendicular to the fiber plane of the crystal (Figure 5f, right). The base-plane diffraction patterns showed well-defined diffraction spots, indicating the long-range ordering of **9mer-III-NN** in the platelet plane. Two dimensions were obtained from three-dimensional ED analysis: *a* = 5.25 Å and *b* = 19.9 Å. However, the third axis could not be determined due to the apparent disorder.

The *a* axis was oriented around the fiber axis of the crystal; its length was consistent with that of the height of a single **9mer-III-NN** in the folded conformation (∼4.8 Å between ring centroids of chitin strands). This suggested that the molecules assemble into stacks along the fiber axis, driven by the intermolecular hydrogen bonding between the amidic oxygens and the N-hydrogens, as well as the hydrophobic interactions between the C–H rich faces of GlcNAc, similarly to the natural chitin allomorphs (Figure 3a and Figure S23). The molecular stacks further assemble along the transversal direction (i.e., *b* axis). The *b* axis is roughly twice as long as the width of the folded **9mer-III-NN**. This crystallographic feature is similar to that of α-chitin^52^ and thus implies antiparallel packing of the **9mer**-**III-NN** molecules in the crystal, with molecular directionality alternating between adjacent molecular stacks. With this information, we propose a tentative molecular packing model (Figure 5g); the full crystallographic determination may require a higher-resolution diffraction data set based on larger and more pristine crystallites.

We compared the crystallites obtained from **9mer**-**III-NN** with those obtained from linear chitin sequences (Figure S42).^53^ First, much longer linear sequences (at least six GlcNAc units) were necessary to promote supramolecular aggregation, while **9mer-III-NN** has only three GlcNAc units in the strands. Second, while both samples preserved the α-chitin-like organization, the crystals obtained from linear oligomers were much smaller in particle dimensions than those obtained from **9mer-III-NN**. Together, these results suggested that preorganization is beneficial for regular assembly.

## Conclusions

Glycan–glycan interactions play significant roles in cell communication^54^ and structural support.^55,56^ We demonstrated that these forces could be leveraged to tune the rigidity, stability, and higher-order organization of much shorter glycan oligomers. By designing glycan hairpins that incorporate oligomers representative of natural polysaccharide as strands, we developed a modular platform to qualitatively compare their strengths. MD simulations indicated that hairpins built with chitin strands adopt an extremely rigid conformation, stabilized by intramolecular H-bonds between the amide side chains on opposite strands. In contrast, cellulose and xylan strands, as well as combination of those, result in more flexible compounds.

Our computational screening identified **9mer-III-NN** as soluble model of crystalline chitin as well as an interesting target for the development of glycan foldamers with highly rigid secondary structures. NMR and SAXS experiments on the synthetic compound unequivocally confirmed the MD prediction, validating a folded conformation sustained by N–H···O H-bonds. These results also suggest that *N*-acetylated monosaccharides could be strategically incorporated into glycan sequences to stabilize secondary structures, with implication in glycomimetic^57^ and glycan catalyst design.^58^

SAXS analysis at 1.0% w/v revealed supramolecular aggregation consistent with intermolecular interactions between the chitin chains. Crystallization of **9mer-III-NN** and subsequent TEM analysis revealed the formation of micrometer-long platelet-like structures. ED analysis allowed for the construction of a molecular packing model in which hairpin molecules stack along the fiber axis, driven by intermolecular interactions between chitin chains. Overall, these findings highlight chitin oligomers and, more generally, *N*-acetylated monosaccharides as useful building blocks for designing rigid glycan-based architectures.

## Experimental Section

### MD Simulations

All simulations employed the modified GLYCAM06_OSMO,r14_.^59^ Initial conformations for single-hairpin systems were constructed using the Glycam Carbohydrate builder in combination with *tleap* (https://glycam.org/), and the resulting topologies were converted via the *acpype* Python script. All simulations were conducted in an explicit solvent using the TIP5P water model.^60^ The simulation time for the single-molecule experiments was 500 ns. Covalent bonds involving hydrogen atoms were constrained using the LINCS algorithm, enabling a 2 fs integration time step. A cutoff of 1.4 nm was applied to nonbonded interactions, while long-range electrostatics were calculated using the particle mesh Ewald (PME) method.^61^ Following energy minimization with the steepest descent algorithm, systems were equilibrated for 50 ns at 300 K in the canonical (NVT) ensemble followed by an additional 50 ns equilibration at 300 K and 1 bar in the isothermal–isobaric (NPT) ensemble. All molecular dynamics simulations were performed using Gromacs 5.1.2.^62^ Temperature was maintained using a Nosé–Hoover thermostat^63^ at 303 K, and pressure was controlled at 1 bar using a Parrinello–Rahman barostat.^64^ Trajectory analysis and visualization were carried out in OriginPro 2021b. Representative three-dimensional structures were extracted using the Glycan Analysis Pipeline (GAP).^65^ More details on MD simulations are provided in Supporting Information Section 1.

### Synthesis

The oligosaccharides were synthesized using a home-built automated synthesizer designed at the Max Planck Institute of Colloids and Interface.^66^ Detailed procedures for BBs synthesis, AGA modules, and post-AGA transformations are provided in Supporting Information Sections 2 and 3.

### NMR Analysis

^1^H, ^13^C, HSQC, 1D and 2D TOCSY, 2D ROESY, and 2D NOESY NMR spectra were acquired on Bruker Biospin AVANCE700 (700 MHz) and Bruker AVANCE III 800 (800 MHz) spectrometers. Samples were prepared by dissolving lyophilized material in D_2_O at concentrations ≈ 1–4 mM. Proton resonances of the oligosaccharides were assigned using a combination of ^1^H, 2D COSY, HSQC, and 1D and 2D TOCSY experiments. Selective 1D TOCSY (HOHAHA, pulse program: seldigpzs) spectra were recorded with mixing times of d9 = 40, 80, 120, 160, and 200 ms to enable complete resonance assignments. 2D TOCSY spectra (pulse program: mlevphpp) were acquired with a mixing time of d9 = 150 ms. Selective 1D t-ROESY (pulse program: selrogp.2) and 2D t-ROESY (pulse program: reosyph.2) experiments were recorded with a mixing time of p15 = 300 ms. 2D NOESY spectra were obtained with a mixing time of d8 = 400 and 600 ms. The full NMR characterization of all glycans is provided in Supporting Information Section 4.

### SAXS Analysis

X-ray scattering experiments were conducted at the BM26 beamline of the European Synchrotron Radiation Facility (ESRF). Samples of **9mer-III-NN** at concentrations of 0.25 and 1.0% wt were prepared at 25 **°**C, sealed in glass capillaries, and mounted on a motorized sample changer. Measurements were carried out using monochromatic X-rays of 12 keV (λ = 1.033 Å). Scattering intensities were recorded using a two-dimensional pixel detector (Pilatus1M, Dectris). The data processing was performed using the pyFAI software,^67^ and subsequent analysis, including intensity profiles and Rg determination, was conducted using the Gnuplot software.

Explicit-solvent SAXS calculations were carried out using a modified version^68,69^ of GROMACS 2022.2 (GROMACS-SWAXS).^70^ Further details on these calculation can be found in a previous publication.^71^ In this modified GROMACS code, the solvent molecules in the solvation layer were included in the SAXS intensity calculations, unlike the conventional method that considers only solute molecules. This explicit solvent approach yields more realistic simulated intensities since the density fluctuations of water molecules surrounding the solute contribute significantly to the overall SAXS intensities. A spatial envelope was constructed around the hairpin at a distance of 0.7 nm. The water subtraction was performed using >1000 simulation frames obtained from pure-water simulation box. The atomic form factors were approximated by using the Cromer–Mann expression:

$$f_{j}(q)=\sum_{k=1}^{4}c+a_{k}e^{-b_{k}{\left(\frac{q}{4\pi }\right)}^{2}}$$

where the values *a*_*k*_, *b*_*k*_, and *c* correspond to the Cromer–Mann parameters. Orientational averaging was conducted using 500 *q*-vectors for each magnitude of *q*, and the solvent electron density was adjusted to the experimental value of 334 e/nm^3^. Additional details on the SAXS analysis are provided in Supporting Information Section 5.

### TEM Analysis

Transmission electron microscopy (TEM) analyses were carried out on a JEOL JEM F200 (Jeol, Japan) (S)­TEM equipped with a field emission gun and a TVIPS TemCam-F126 (2k × 2k) camera. The microscope was operated at 80 kV, and a condenser aperture with a diameter of 200 μm was used. For specimen preparation, 2 μL of the sample suspension in solvent was drop-casted on glow-discharged carbon-coated copper grids (Micro to Nano BV, Netherlands). Additional experimental details are provided in Supporting Information Section 6.

### ED Analysis

Drops (2 μL) of solvent suspensions of samples was deposited on a glow-discharged carbon-coated copper grid. TEM and ED experiments were performed using a JEM-2100Plus and JEM F200 cryo transmission electron microscopes (JEOL Ltd., Japan) operated at an accelerating voltage of 200 kV. A Merlin 2D hybrid pixel detector (Quantum Detectors) with a pixel size of 55 × 55 μm^2^ was used to acquire a series of electron diffraction patterns as the sample continuously rotated within the microscope. The tilt-series ED data were processed using the *PETS2* ^72^ software.

## Supplementary Material



## Data Availability

The authors declare that all data supporting the findings of this study are available within the article and in the Supporting Information files. Raw data for NMR analysis, SAXS analysis, and MD simulations can be downloaded from 10.17617/3.YHOCVV, Edmond. Data are also available from the corresponding author upon request.

## Notes

All experimental procedures of synthesis and conformational analysis; characterization data, including HPLC, HR-MS, NMR, SAXS, TEM, and ED (PDF)

## ABBREVIATIONS

AGA: automated glycan assembly
BB: building block
COSY: correlation spectroscopy
D-Glc: d-glucose
D-GlcNAc: d-*N*-acetyl glucosamine
D-Xyl: l-xylose
ED: electron diffraction
HPLC: high-performance liquid chromatography
HRMS: high-resolution mass spectrometry
HSQC: heteronuclear single quantum coherence
KDE: kernel density estimation
L-Rha: l-rhamnose
MD: molecular dynamics
NMR: nuclear magnetic resonance
NOE: nuclear Overhauser effect
NOESY: nuclear Overhauser effect spectroscopy
RoG: radius of gyration
RMSD: root-mean-square deviation
SAXS: small-angle X-ray scattering
SNFG: Symbol Nomenclature for Glycans
TEM: transmission electron microscopy
TOCSY: total correlation spectroscopy
