# Supplementary material for: Leveraging Glycan–Glycan Interactions to Tune the Conformation of Glycan Hairpins and Build Rigid 3D Architectures
Source: J Am Chem Soc. 2026 Apr 21;148(17):18433–43. doi: 10.1021/jacs.6c04539 (PMC13154203; doi:10.1021/jacs.6c04539)
Supplement: Supplementary file 1 [file ja6c04539_si_001.pdf]

Supporting Information

Leveraging glycan-glycan interactions to tune the conformation of glycan hairpins and build rigid 3D architectures

Nishu Yadav,[1,2] Yadiel Vázquez-Mena,[1,7] Ana Poveda,[3] Dominik Weh,[1,2] Jesús Jiménez-Barbero,[3,4,5,6] Yu Ogawa,[7,8] and Martina Delbianco[1]*

[1]Department of Biomolecular Systems, Max Planck Institute of Colloids and Interfaces, Am Mühlenberg 1, 14476, Potsdam, Germany

[2]Department of Chemistry and Biochemistry, Freie Universität Berlin, Arnimallee 22, 14195, Berlin, Germany

[3]CICbioGUNE, Basque Research and Technology Alliance, 48160, Derio, Spain

[4]Ikerbasque, Basque Foundation for Science, 48009, Bilbao, Spain

[5]Department of Inorganic & Organic Chemistry, Faculty of Science and Technology, University of the Basque Country, EHU-UPV, 48940, Leioa, Spain

[6]Centro de Investigación Biomedica En Red de Enfermedades Respiratorias, 28029, Madrid, Spain

[7]Univ. Grenoble Alpes, CNRS, CERMAV, 38000, Grenoble, France

[8]Department of Sustainable and Bioinspired Materials, Max Planck Institute of Colloids and Interfaces, Am Mühlenberg 1, 14476, Potsdam, Germany

Table of contents

1 **Molecular dynamics simulations** **4**
1.1 General materials and methods for Molecular dynamics simulations 4
1.2 MD analysis of 9mers with different strands 5
1.3 Clustering analysis 13
1.3.1 9mer-III-AA 13
1.3.2 9mer-III-YY 14
1.3.3 9mer-III-NN 15
1.3.4 9mer-III-A'A' 16
1.4 Comparison of shorter strand hairpin 17
1.5 Chitin models and hairpin model 19
2 **Synthesis and NMR analysis** **20**
2.1 General materials and methods for synthesis and NMR 20
2.2 Building blocks 21
3 **Automated glycan assembly** **22**
3.1 General materials and methods 22
3.2 Preparation of stock solutions 22
3.3 Modules for automated synthesis 22
3.3.1 Module A: Resin preparation 22
3.3.2 Module B: Acidic wash with TMSOTf solution (20 min) 22
3.3.3 Module C1: Thioglycoside glycosylation (35 min-55 min) 23
3.4 Post-AGA manipulations 25
3.4.1 Module F: On-resin methanolysis 25
3.4.2 Module G1: Cleavage from solid support 25
3.4.3 Module G2: Micro-cleavage from solid support 26
3.4.4 Module H: Hydrogenolysis 26
3.4.5 Module I: Purification 26
3.5 Oligosaccharides synthesis 27
3.5.1 Synthesis of 3mer-VI 28
3.5.2 Synthesis of 5mer-III-NN 32
3.5.3 Synthesis of 9mer-III-NN 36
4 **NMR analysis** **43**
4.1 NMR characterization of 5mer-III-NN 43
4.1.1 STEP-t-ROESY inter-residue distance estimation 49
4.2 H-bond study in 5mer-III-NN 51
4.3 H-bond study of 9mer-III-NN 52
4.4 Temperature coefficient comparison for amide protons in 5mer-III-NN & 9mer-III-NN 57
4.5 Proton and carbon chemical shift values of 5mer-III-NN 58

5 SAXS Analysis of 9mer-III-NN **60**
5.1 General materials and methods 60
5.2 SAXS calculation 60
5.3 Experimental Rg calculation 61
6 Triggered assembly of 9mer-III-NN **62**
6.1 Crystallization method 62
6.2 Transmission electron microscopy imaging 62
6.3 ED analysis 63
7 References **64**

1 Molecular dynamics simulations

1.1 General materials and methods for Molecular dynamics simulations

All simulations employed the modified GLYCAM06$_{OSMO,r14}$.[1] Initial conformations for single hairpin systems were constructed using the Glycam Carbohydrate builder in combination with *tleap* (https://glycam.org/), and the resulting topologies were converted via the *acpype* Python script. All simulations were conducted in explicit solvent using the TIP5P water model.[2] The simulation time for the single molecule experiments was 500 ns. Covalent bonds involving hydrogen atoms were constrained using the LINCS algorithm, enabling a 2fs integration time step. A cutoff of 1.4 nm was applied to nonbonded interactions, while long-range electrostatics were calculated using the particle mesh Ewald (PME) method..[3] Following energy minimization with the steepest descent algorithm, systems were equilibrated for 50 ns at 300 K in the canonical (NVT) ensemble, followed by an additional 50 ns equilibration at 300 K and 1 bar in the isothermal–isobaric (NPT) ensemble. All molecular dynamics simulations were performed using Gromacs 5.1.2.[4] Temperature was maintained using a Nosé–Hoover thermostatt[5] at 303 K, and pressure was controlled at 1 bar using a Parrinello–Rahman barostat.[6] Trajectory analysis and visualization were carried out in OriginPro 2021b. Representative three-dimensional structures were extracted using the Glycan Analysis Pipeline (GAP).[7] All hairpins with the free reducing end were modeled as β-anomers.

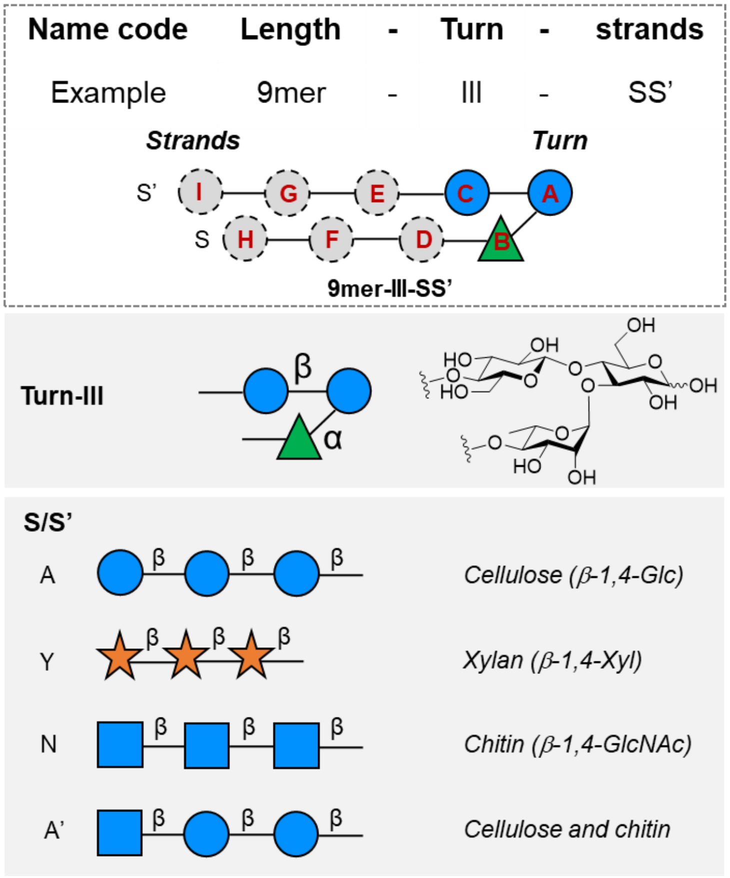

Figure S01 Nomenclature for the structures studied in this work and labeling of the residues with letters (highlighted brown) from reducing to non-reducing.

1.2 MD analysis of 9mers with different strands

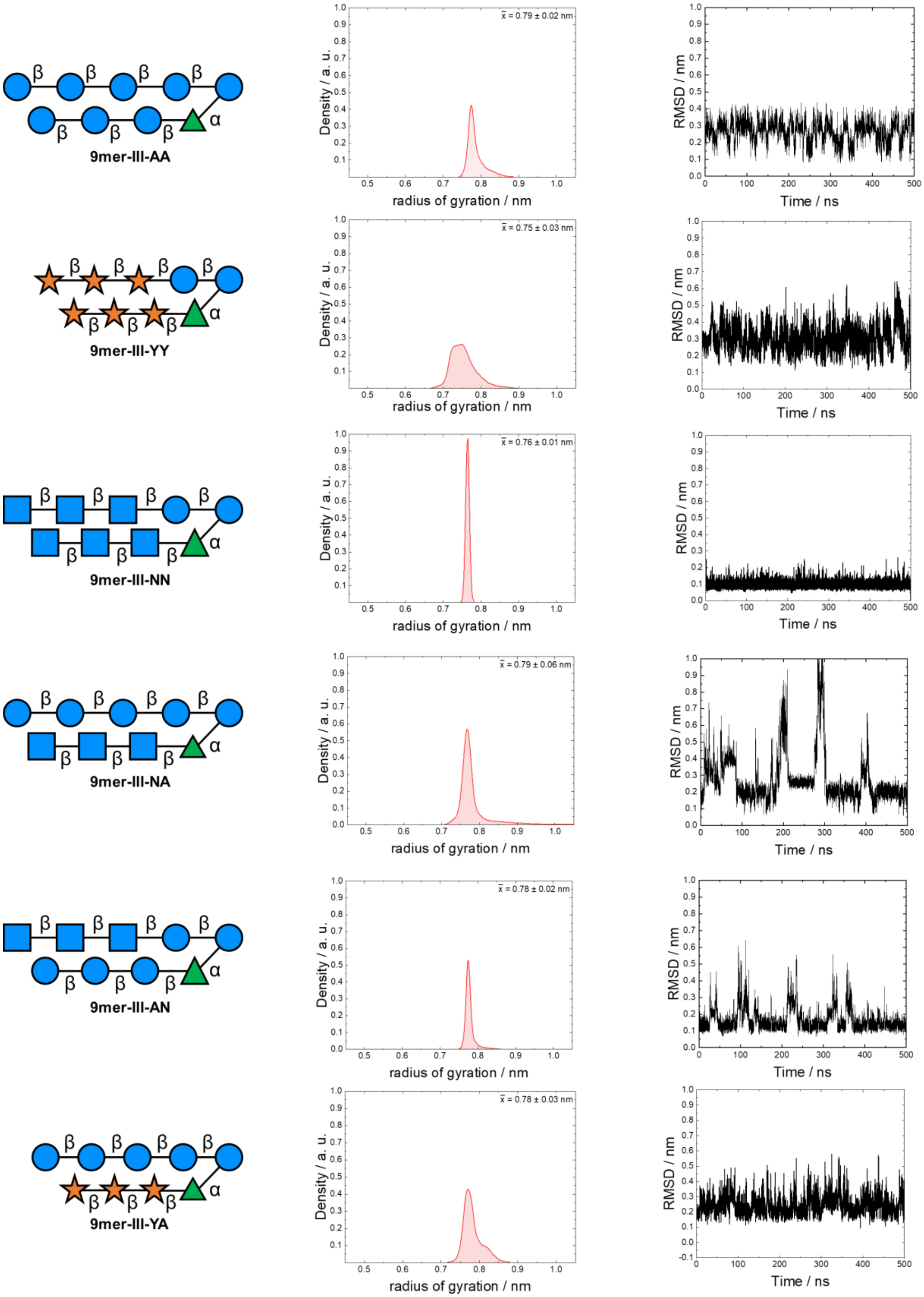

Figure S02 Radius of gyration and root-mean-square deviation (RMSD) analysis of 9mers with different strands.

$$Definition\ \psi = C_1 - O_n - C_n - C_{n-1}$$

$$Definition\ \phi = O_5 - C_1 - O_n - C_n$$

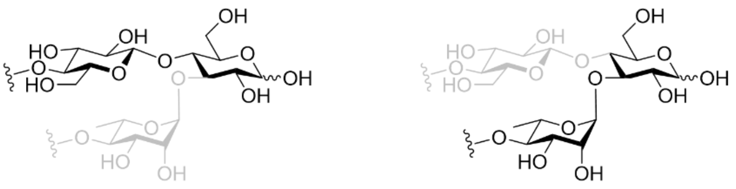

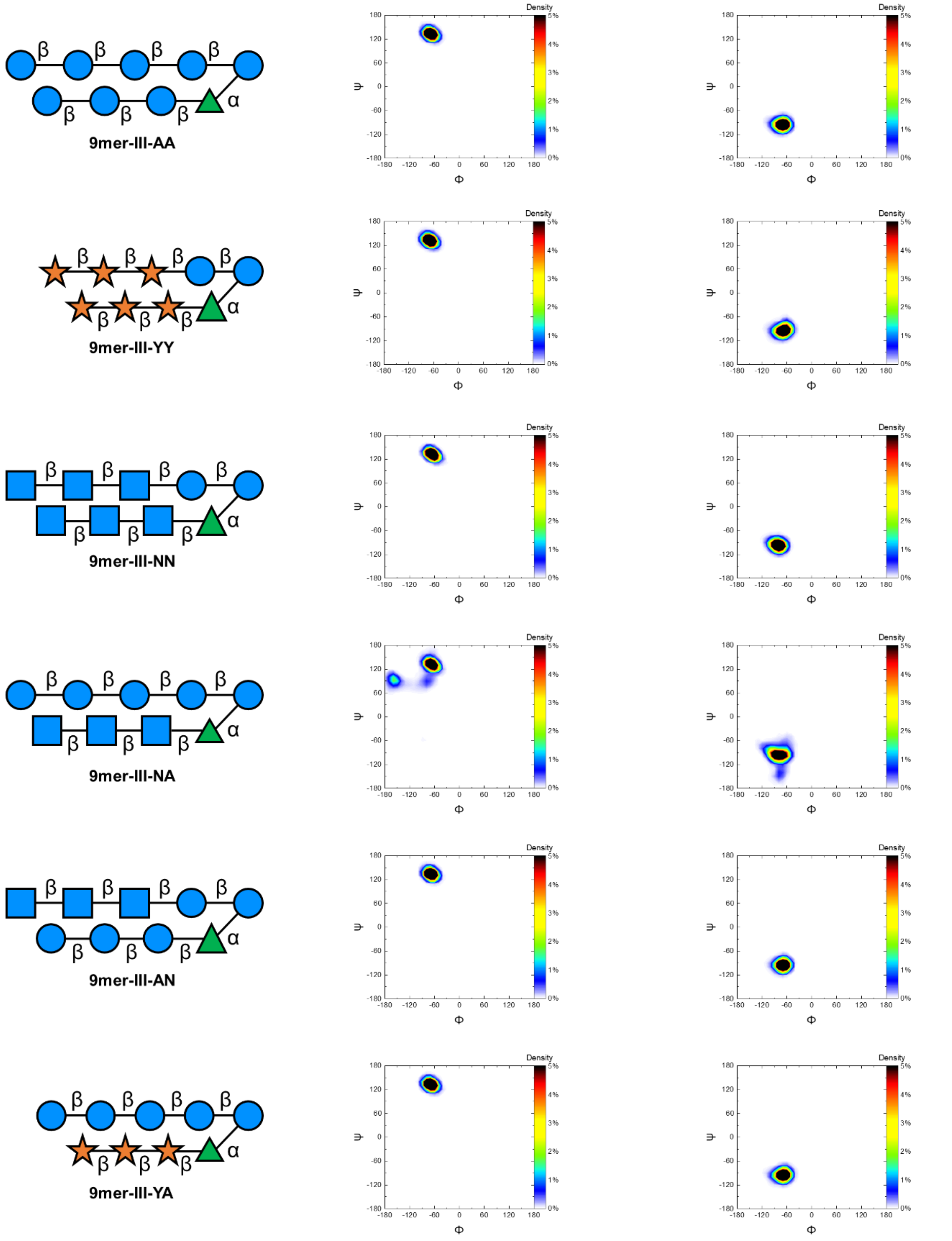

Figure S03 Ramachandran plots of the turn units of 9mers with different strands.

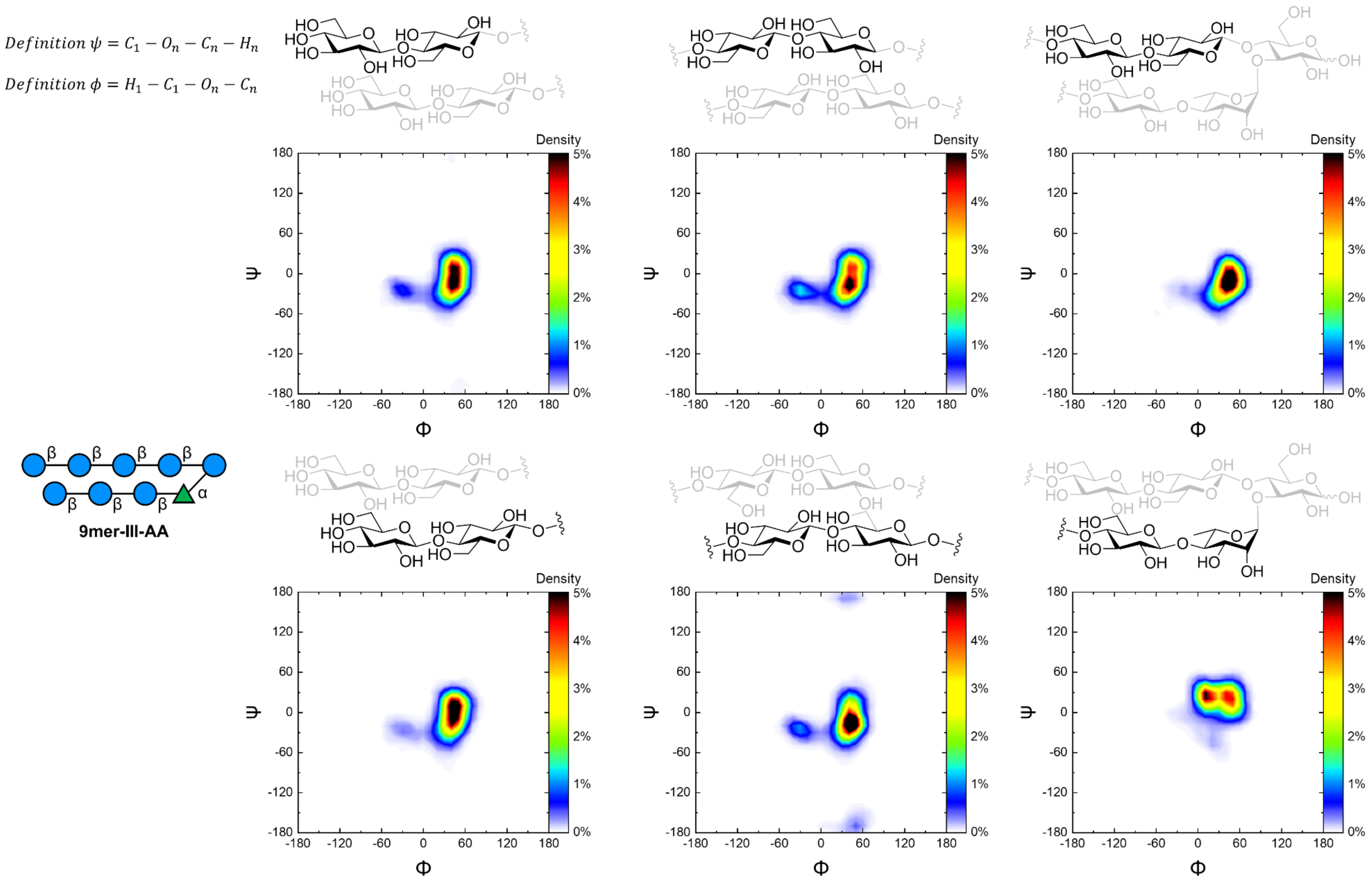

Figure S04 Ramachandran plots of the top and bottom strands of **9mer-III-AA**.

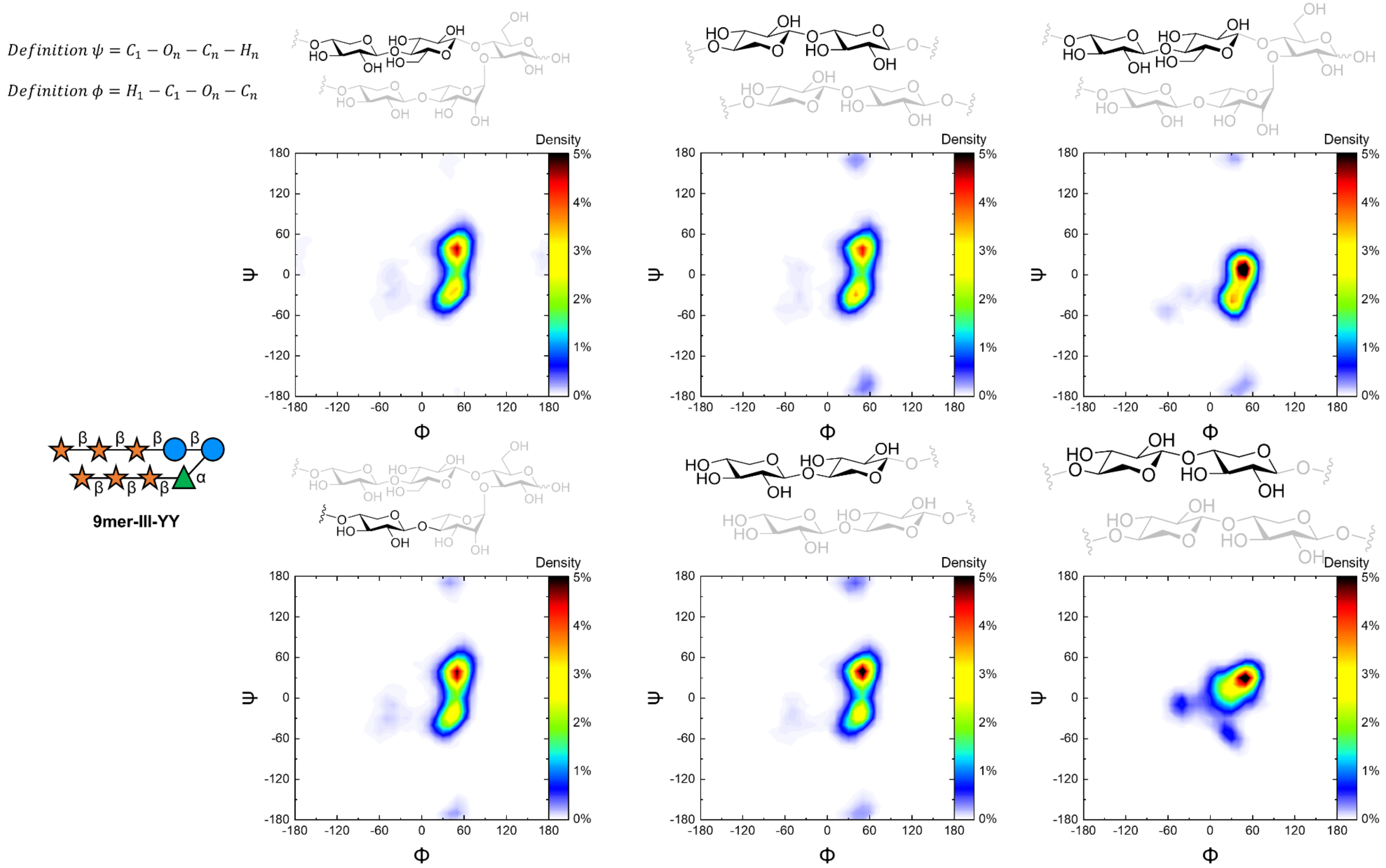

Figure S05 Ramachandran plots of the top and bottom strands of **9mer-III-YY**.

$$Definition\ \psi = C_1 - O_n - C_n - H_n$$

$$Definition\ \phi = H_1 - C_1 - O_n - C_n$$

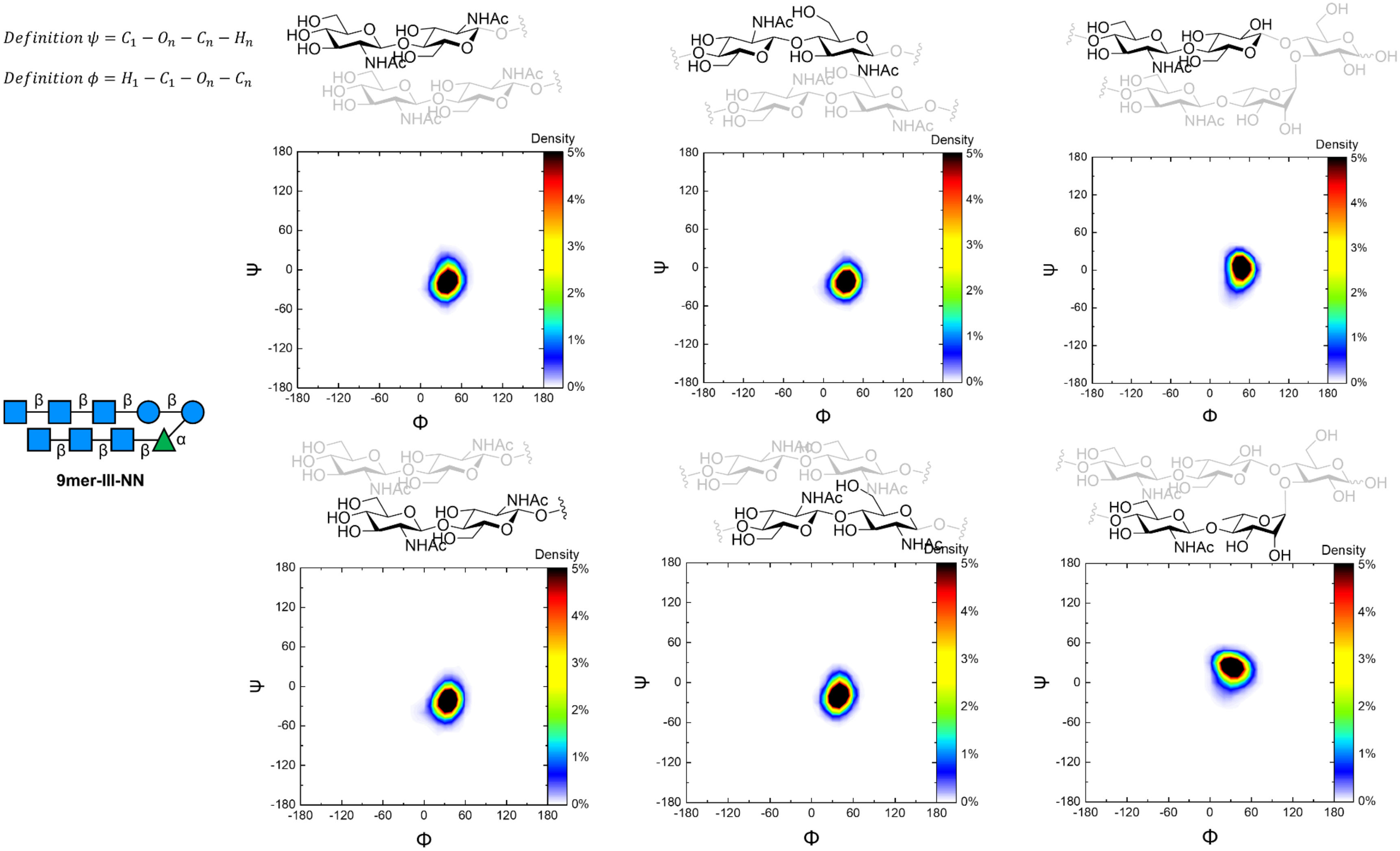

Figure S06 Ramachandran plots of the top and bottom strands of **9mer-III-NN**.

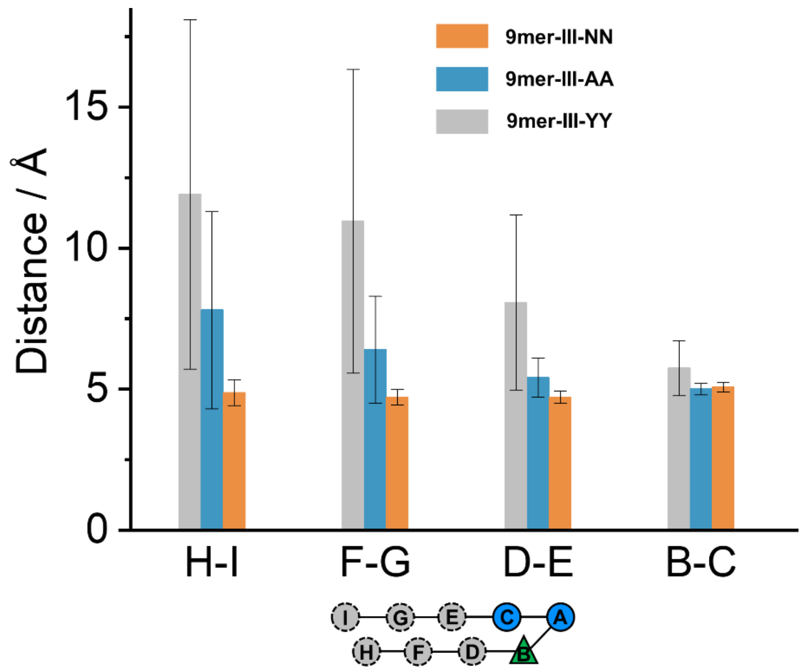

Figure S07 Inter-residue distances comparison of **9mer-III-AA**, **9mer-III-NN** and **9mer-III-YY**.

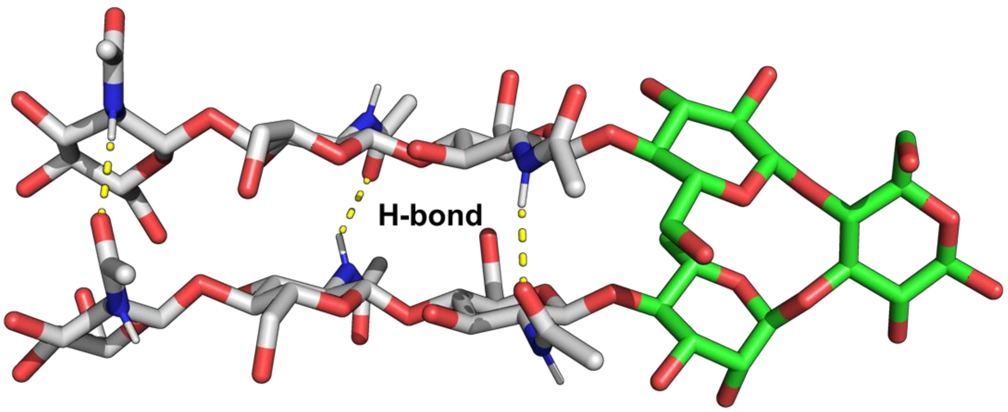

Figure S08 Representative snapshot of **9mer-III-NN** with highlighted (yellow dash line) H-bond between amide oxygen of one strand and hydrogen (N-H) of another strand.

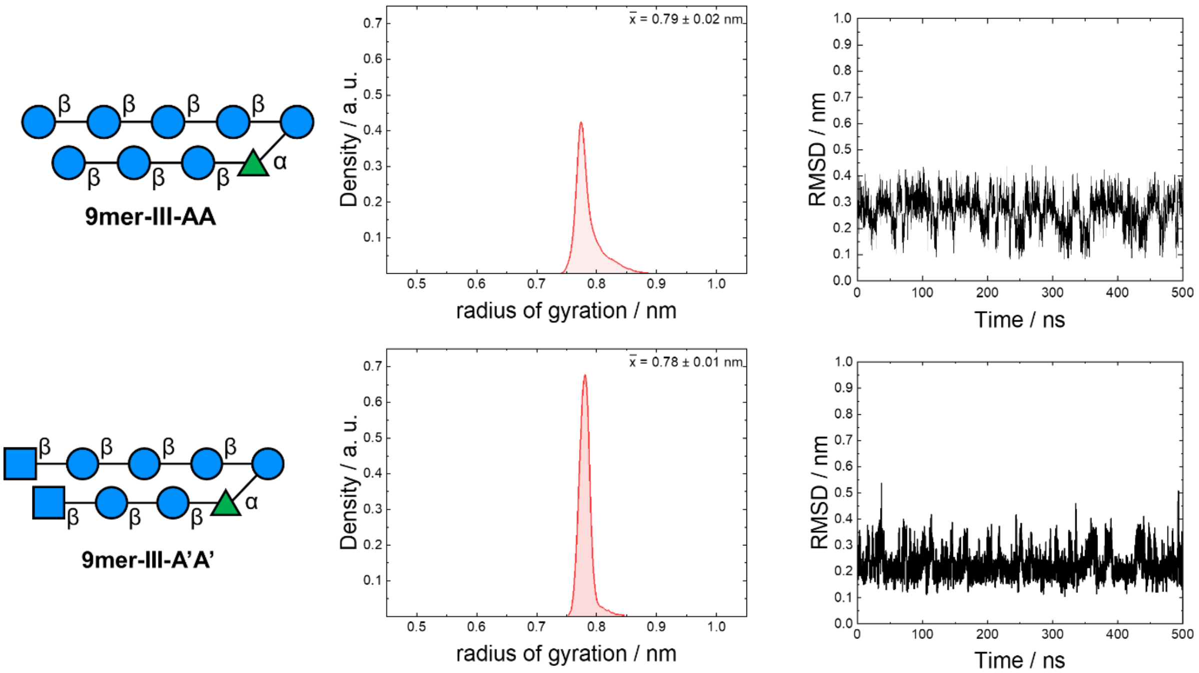

Figure S09 Radius of gyration and root-mean-square deviation (RMSD) analysis of **9mer-III-AA** and **9mer-III-A'A'**.

$Definition\ \psi = C_1 - O_n - C_n - H_n$

$Definition\ \phi = H_1 - C_1 - O_n - C_n$

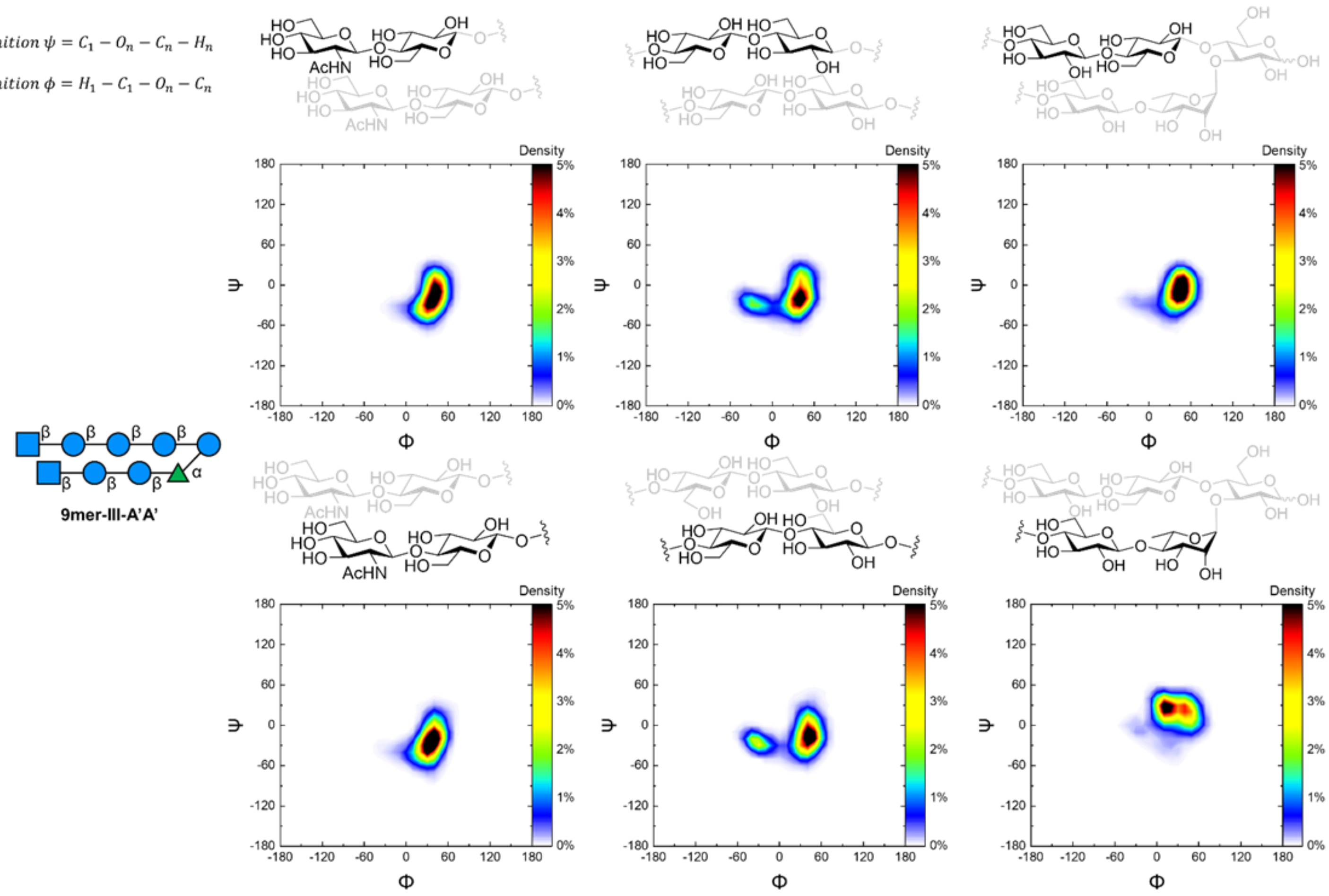

Figure S10 Ramachandran plots of the top and bottom strands of **9mer-III-A'A'**.

1.3 Clustering analysis

1.3.1 9mer-III-AA

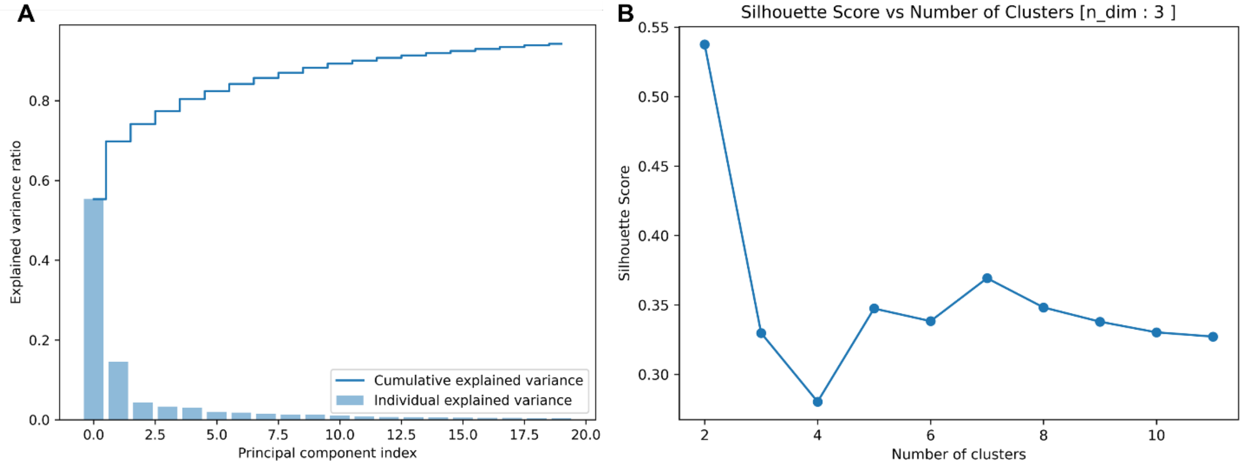

Figures S11 a) Cumulative and individual explained variance by principal components. **b)** Silhouette score analysis for determining the optimal numbers of clusters, with the peak at 7 clusters suggesting it as the optimal count for the given dataset.

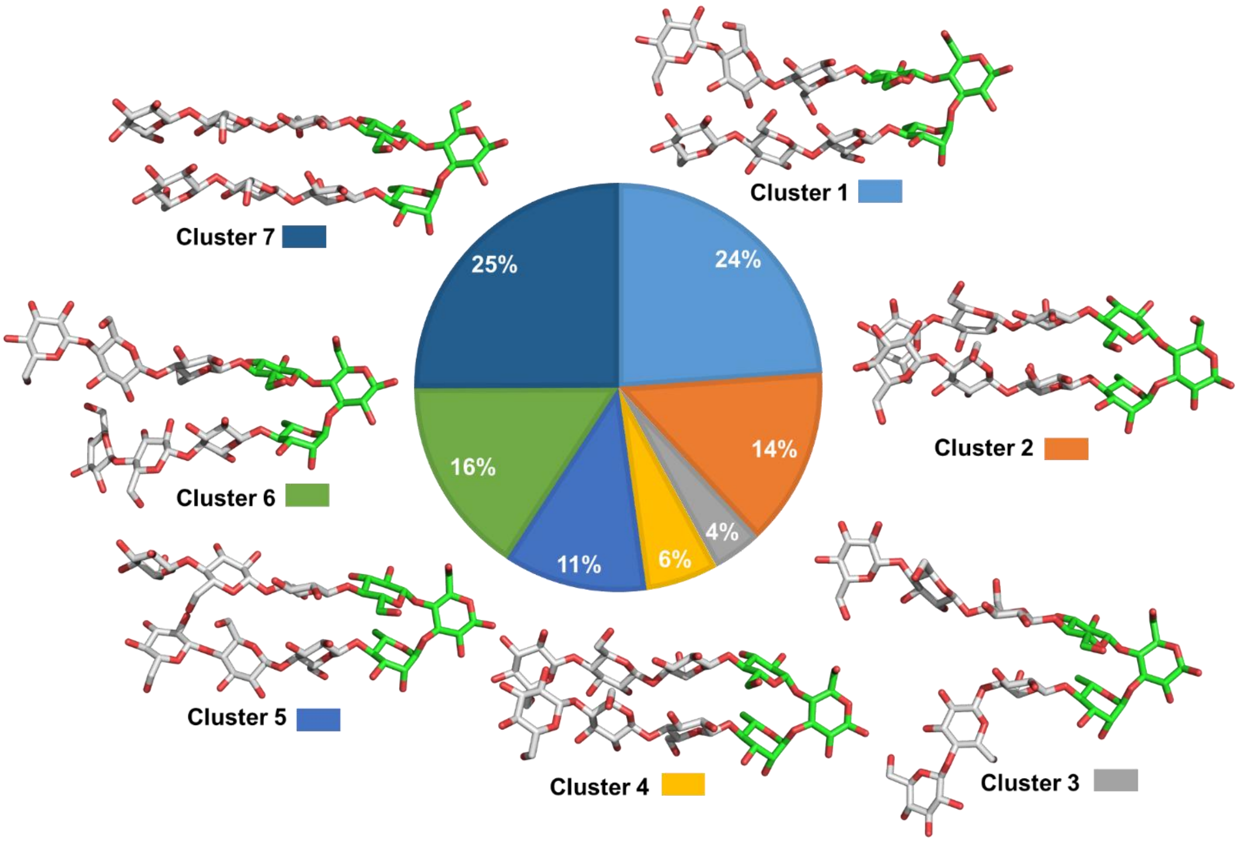

Figure S12 Pie chart showing the cluster distributions together with representative 3D structures for each cluster selected on the basis of KDE (kernel density estimation) maxima.

1.3.2 9mer-III-YY

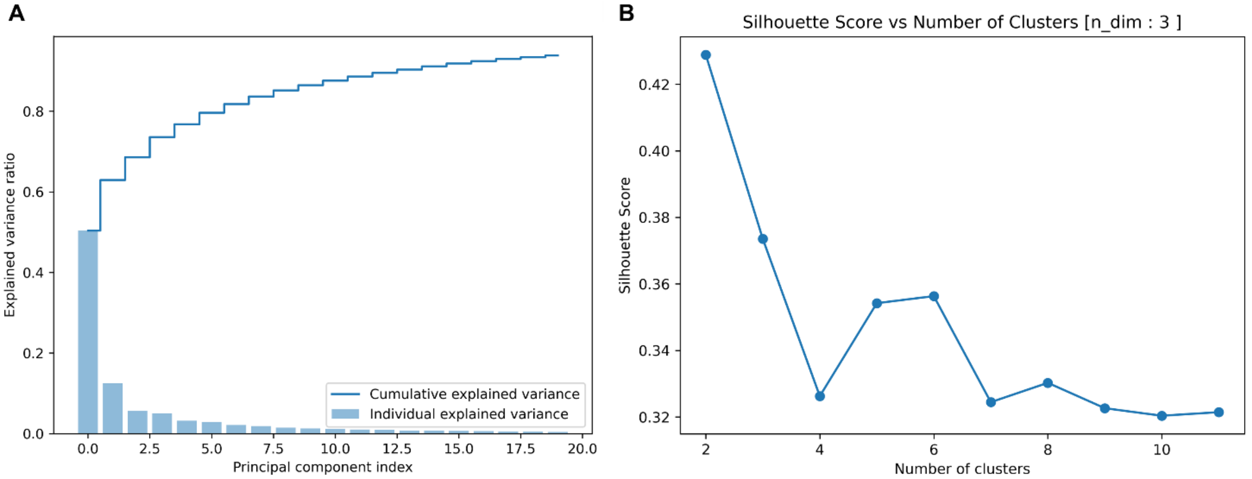

Figures S13 a) Cumulative and individual explained variance by principal components. **b)** Silhouette score analysis for determining the optimal numbers of clusters, with the peak at 6 clusters suggesting it as the optimal count for the given dataset.

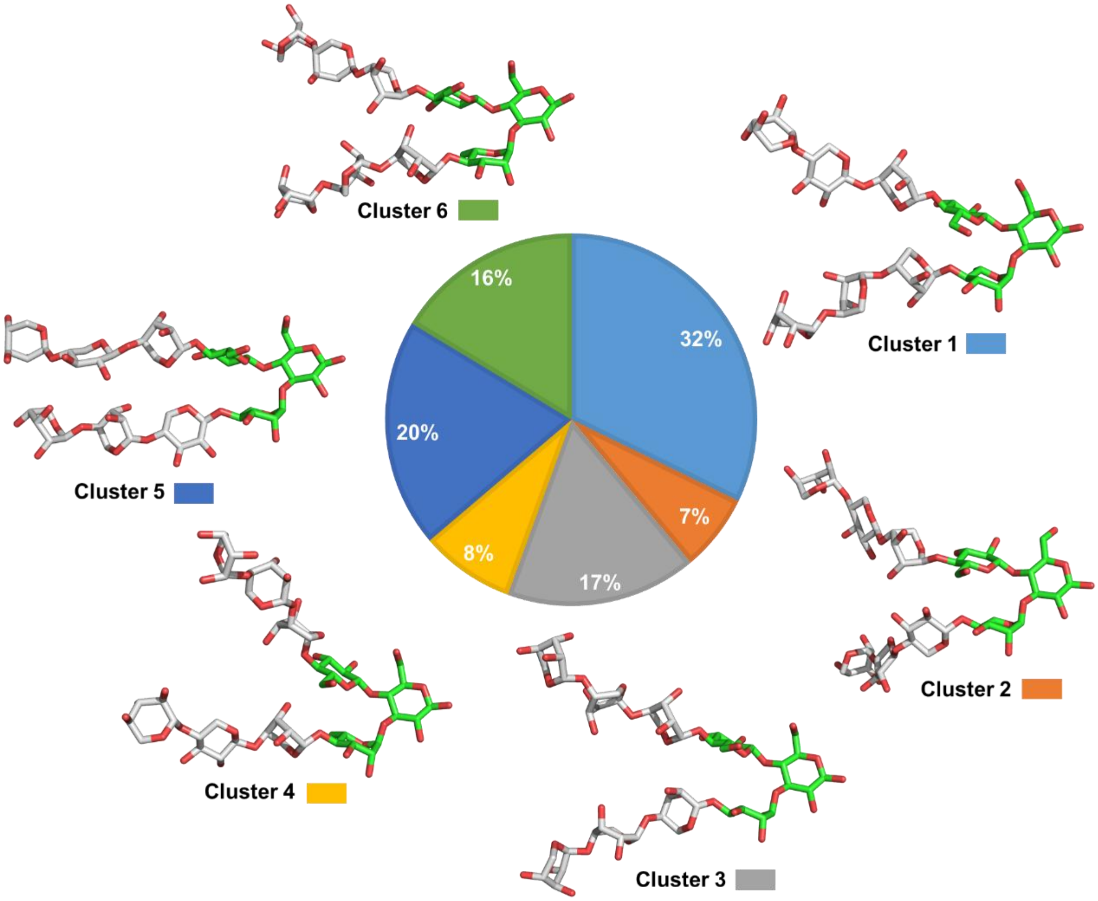

Figure S14 Pie chart showing the cluster distributions together with representative 3D structures for each cluster selected on the basis of KDE (kernel density estimation) maxima.

1.3.3 9mer-III-NN

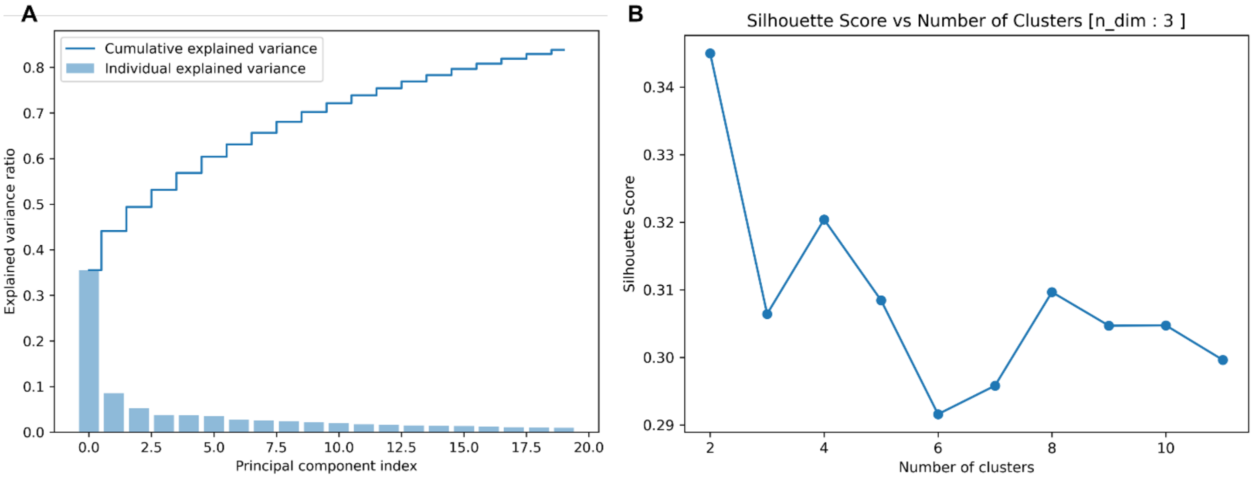

Figures S15 a) Cumulative and individual explained variance by principal components. **b)** Silhouette score analysis for determining the optimal numbers of clusters, with the peak at 4 clusters suggesting it as the optimal count for the given dataset.

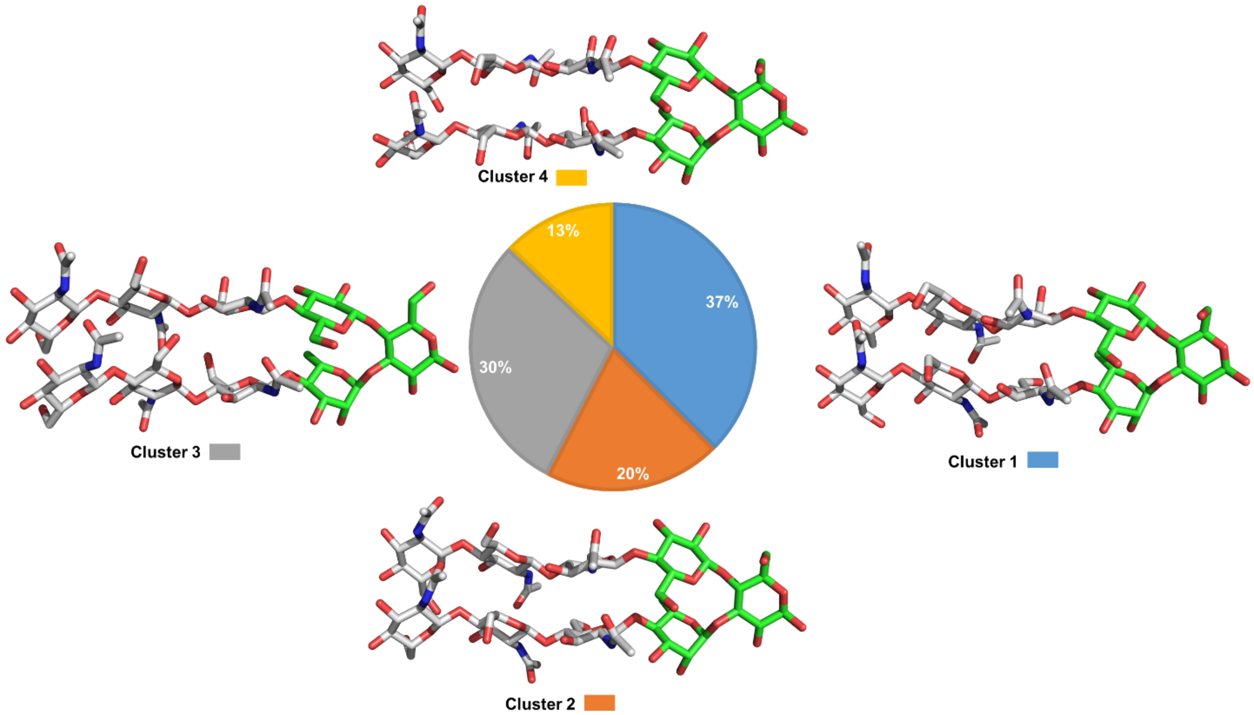

Figure S16 Pie chart showing the cluster distributions together with representative 3D structures for each cluster are selected on the basis of KDE (kernel density estimation) maxima.

1.3.4 9mer-III-A'A'

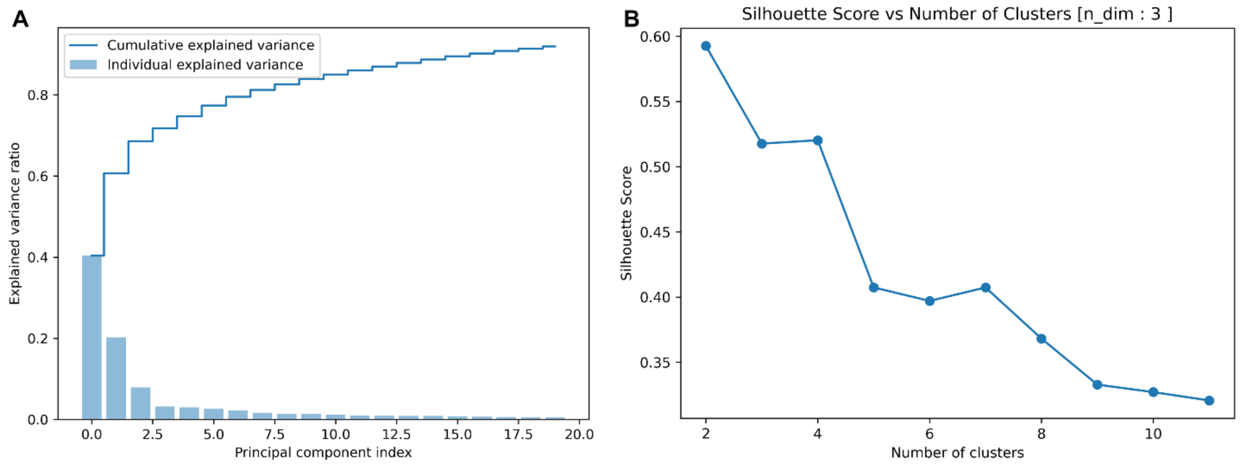

Figures S17 a) Cumulative and individual explained variance by principal components. **b)** Silhouette score analysis for determining the optimal numbers of clusters, with the peak at 4 clusters suggesting it as the optimal count for the given dataset.

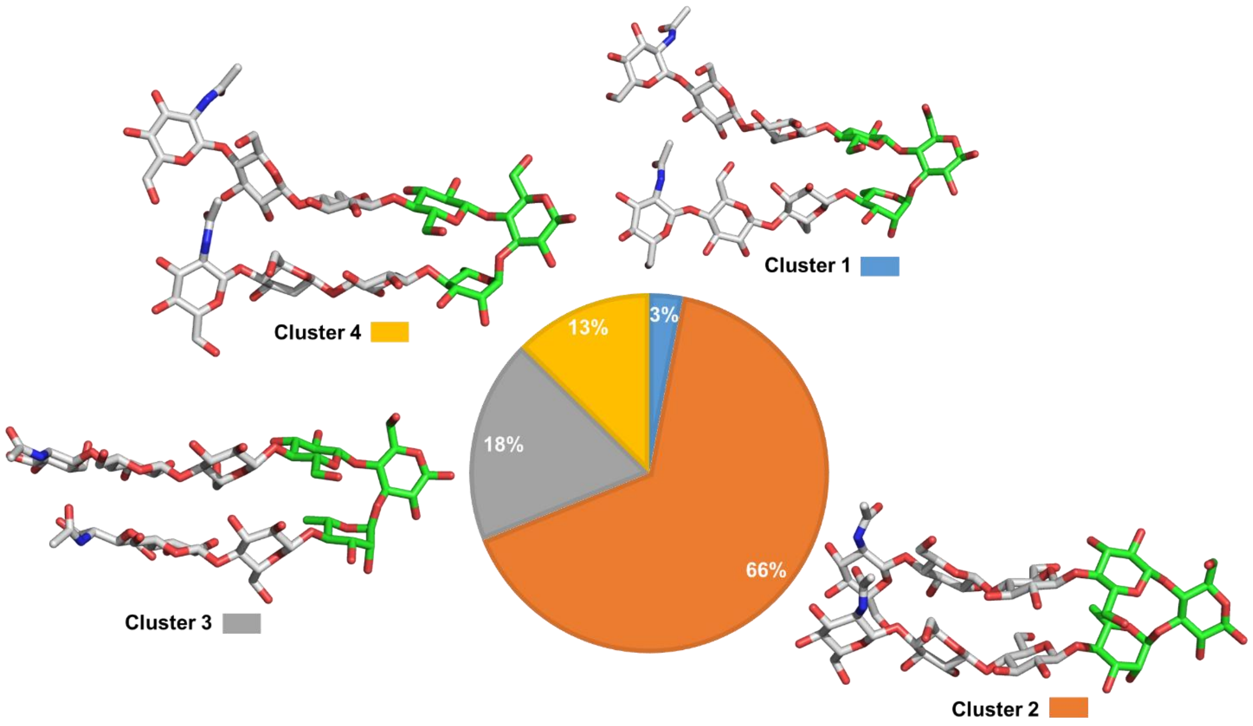

Figure S18 Pie chart showing the cluster distributions together with representative 3D structures for each cluster are selected on the basis of KDE (kernel density estimation) maxima.

1.4 Comparison of shorter strand hairpin

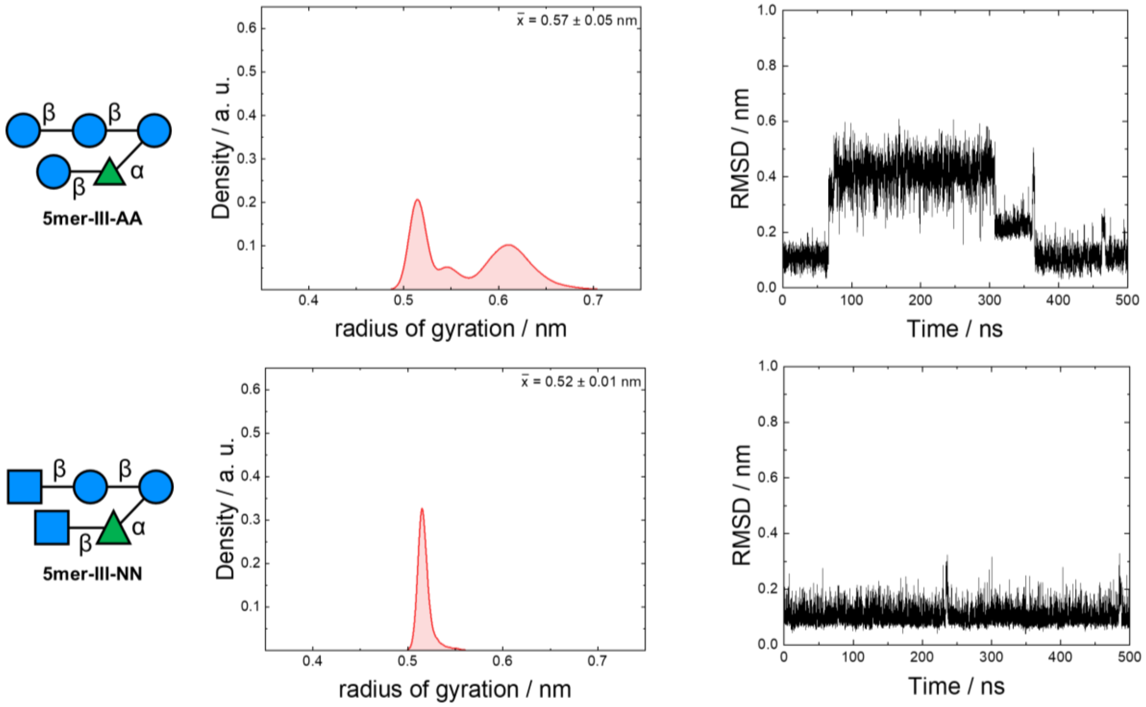

Figure S19 Radius of gyration and root-mean-square deviation (RMSD) analysis comparison of **5mer-III-AA** and **5mer-III-NN**.

$$Definition\ \psi = C_1 - O_n - C_n - C_{n-1}$$

$$Definition\ \phi = O_5 - C_1 - O_n - C_n$$

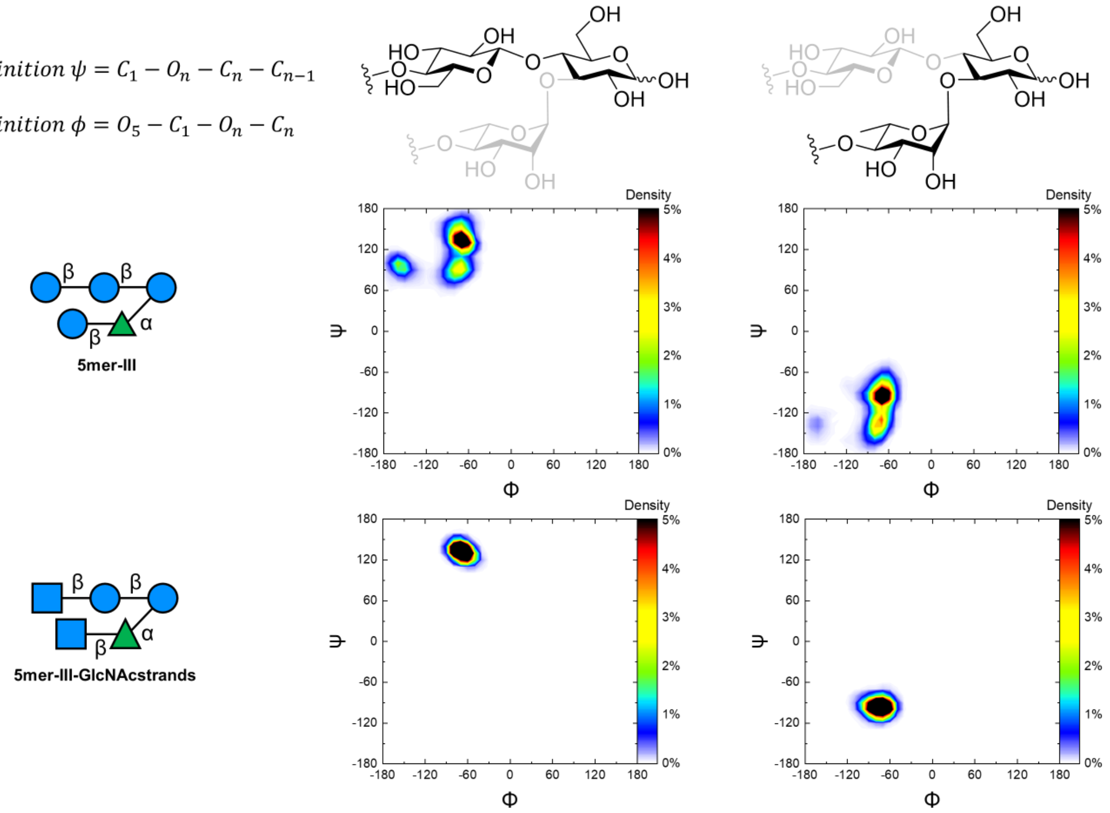

Figure S20 Ramachandran plots of the turn units of **5mer-III-AA** and **5mer-III-NN**.

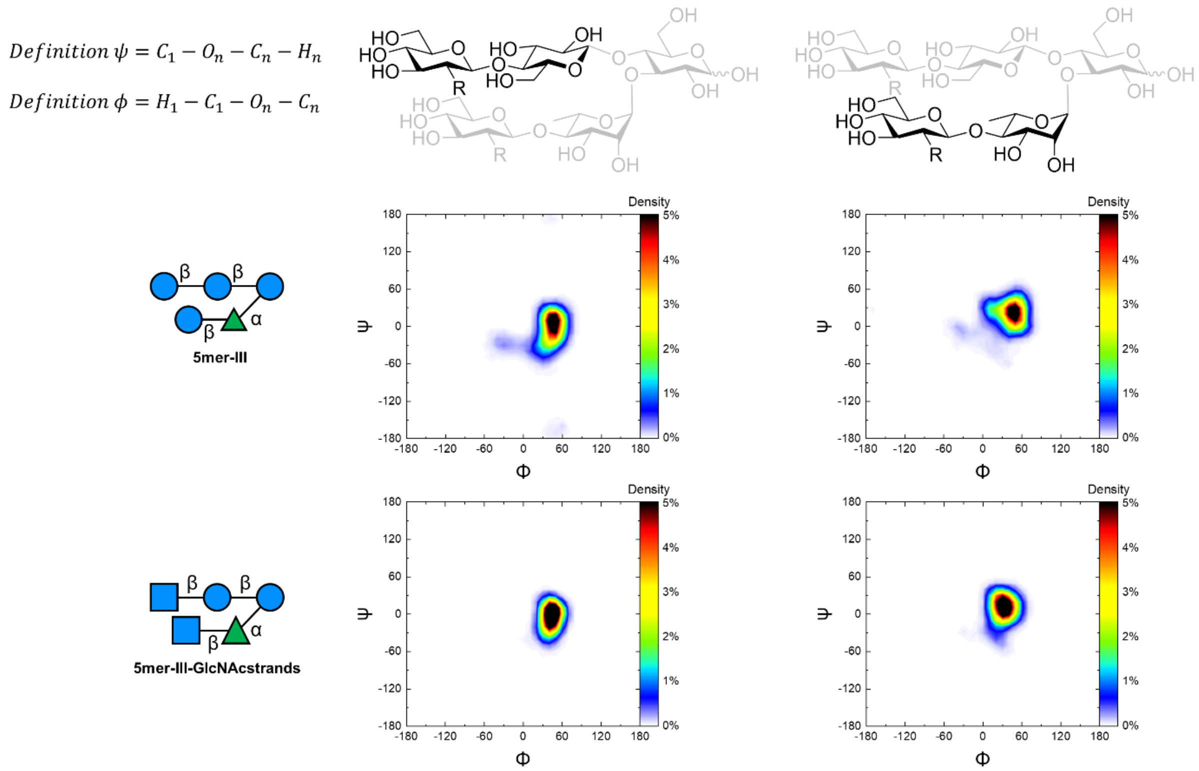

Figure S21 Ramachandran plots of the strands of **5mer-III-AA** and **5mer-III-NN**.

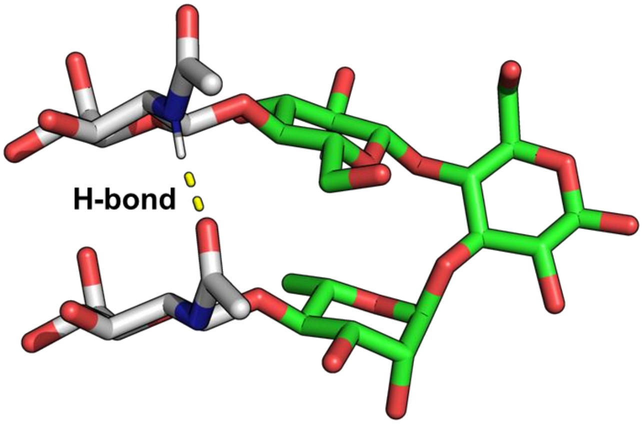

Figure S22 Representative snapshot of **5mer-III-NN** with highlighted (pale yellow dash line) H-bond between amide oxygen of one strand and hydrogen (N-H) of the other strand.

1.5 Chitin models and hairpin model

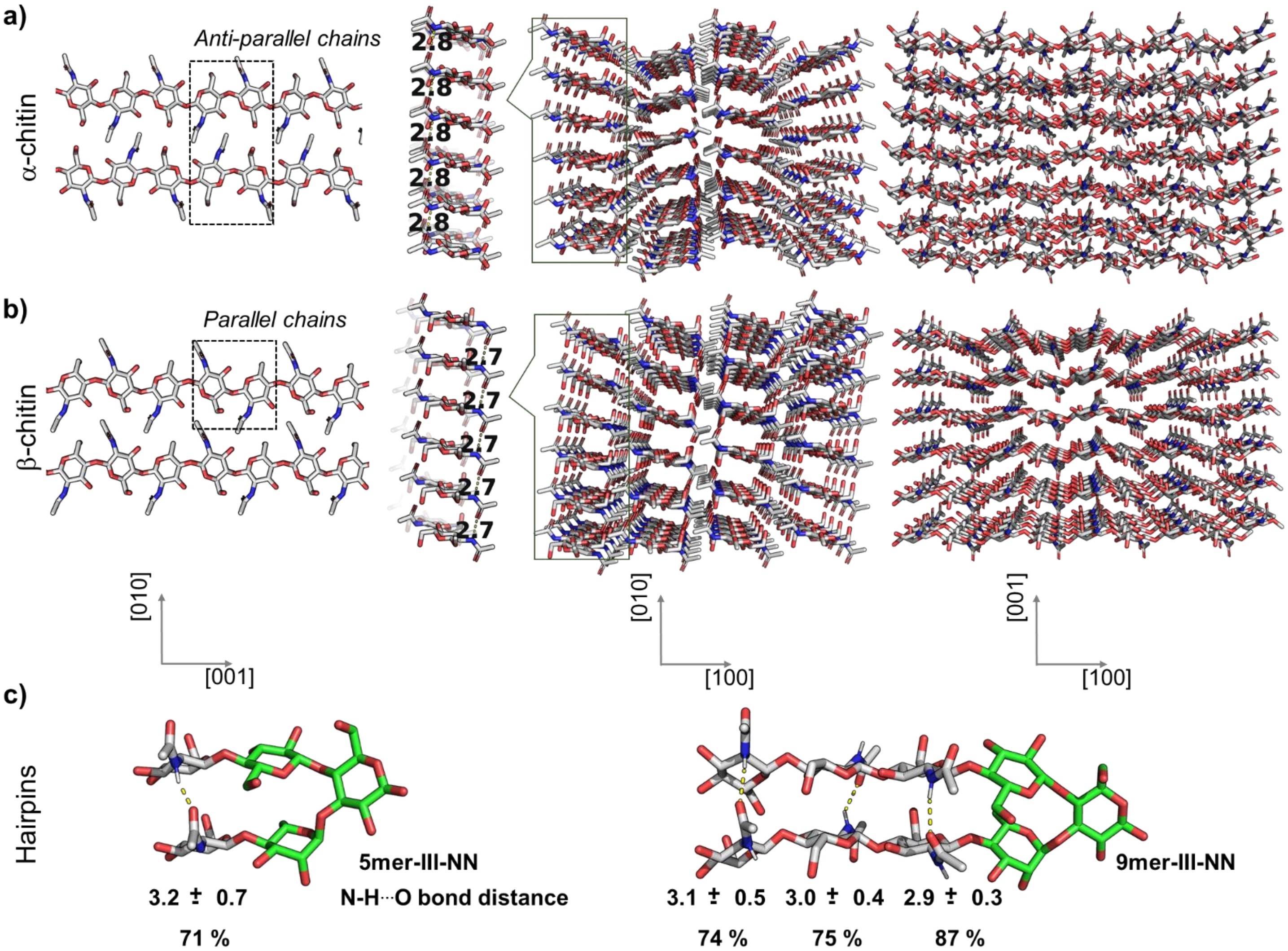

Figure S23 Atomic models of chitin polymorphs and glycan hairpins. **a)** α-chitin **b)** β-chitin **c)** Parallel glycan hairpins. The unit cells are represented by the dashed black boxes. The interchain H-bond distance (**N**−H···**O**=C) for α- and β-chitin are calculated from the model generated based on the crystallographic data obtained by experiments[8, 9] and for the hairpin models (**5mer-III-NN** and **9mer-III-NN**) average interchain H-bond (**N**−H···**O**=C) distances are calculated from the overall MD simulation.

2 Synthesis and NMR analysis

2.1 General materials and methods for synthesis and NMR

All chemicals used were reagent grade and used as supplied unless otherwise noted. The automated syntheses were performed on a home-built synthesizer developed at the Max Planck Institute of Colloids and Interfaces.[10] Analysis and purification by reverse phase HPLC were performed by using an Agilent 1200 series. Products were lyophilized using a Christ Alpha 2-4 LD plus freeze dryer. High resolution mass spectra were obtained using a 6210 ESI-TOF mass spectrometer (Agilent) and a MALDI-TOF autoflexTM (Bruker). ^{1}H, ^{13}C, HSQC, 1D and 2D TOCSY, 1D and 2D ROESY NMR spectra were recorded on a Bruker Biospin AVANCE700 (700 MHz) and Bruker AVANCE III 800 (800 MHz) spectrometer. Samples were prepared by dissolving lyophilized samples in D_2O (concentration ≈ 1 - 4 mM). Spectra were recorded using the solvent as the internal standard in ^{1}H NMR (D_2O: 4.79 ppm ^{1}H). ^{1}H NMR spectra for all compounds were recorded without ^{13}C decoupling. Weak intensity ^{13}C resonances were derived from the respective HSQC cross peaks. ^{1}H NMR integrals of the resonances corresponding to residues at the reducing end are reported as non-integer numbers and the sum of the integrals of α and β anomers is set to 1. Proton resonances of the oligosaccharides were assigned using a combination of ^{1}H, 2D COSY, HSQC, 1D and 2D TOCSY. Selective 1D TOCSY (HOHAHA, pulse program: seldigpzs) spectra were recorded using different mixing times to assign all the resonances (d9 = 40, 80, 120, 160, and 200 ms). 2D TOCSY (pulse program: mlevphpp) spectra were recorded using mixing time (d9 = 150 ms). Selective 1D t-ROESY (pulse program: selrogp.2) and 2D t-ROESY (pulse program: reosyph.2) spectra were recorded using mixing time (p15 = 300 ms). 2D NOESY spectra were recorded using mixing time (d8 = 400, and 600 ms). Monosaccharide were named as follows: D-glucose (Glc), D-*N*-acetyl glucosamine (GlcNAc), L-rhamnose (Rha) and D-Xylose (Xyl). Labelling of protons in a monosaccharide is done as follows: e.g. proton attached to C-1 of Rha at residue B is named "Rha B-1". Resonances of residues at the reducing end are additionally labelled with α or β.

2.2 Building blocks

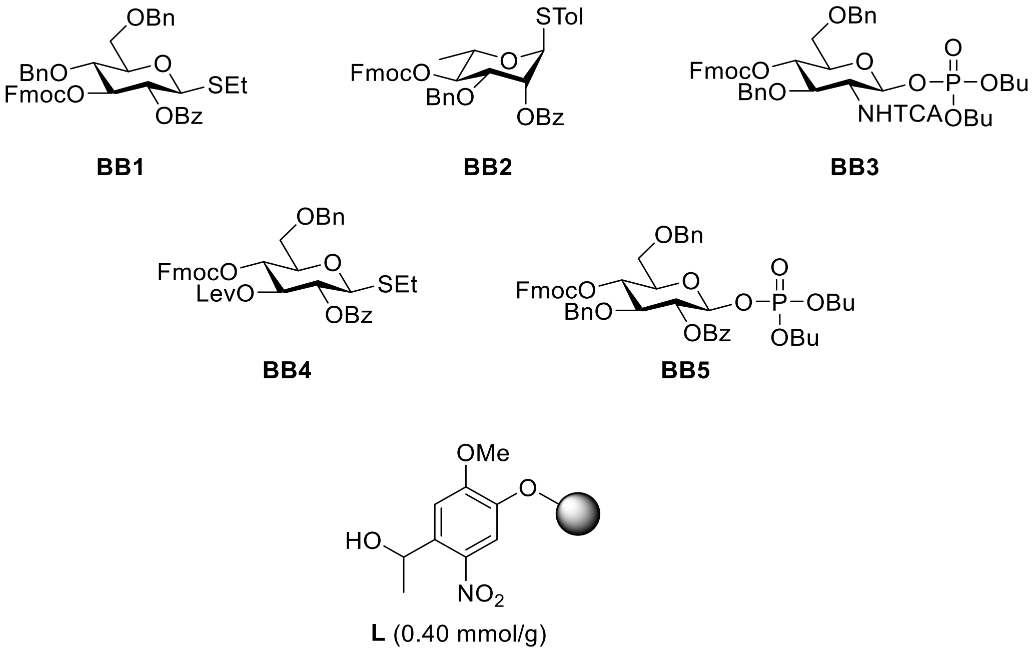

Figure S24 BBs and solid supports used in this work. Loading of **L** is reported in parenthesis.

BB1 was purchased from GlycoUniverse (Germany). **BB2, BB3, BB4** and **BB5** were synthesized according to previously reported procedures.[11] Merrifield resin equipped with photocleavable linkers **L** (loading 0.40 mmol/g) was prepared according to previously reported procedures.[12]

3 Automated glycan assembly

3.1 General materials and methods

The automated syntheses were performed on a home-built synthesizer developed at the Max Planck Institute of Colloids and Interfaces.[10] All solvents used were HPLC-grade. The solvents used for the building blocks, activator, TMSOTf and capping solutions were taken from an anhydrous solvent system (J.C. Meyer). The building blocks were co-evaporated three times with toluene and dried for 1 h on high vacuum before use. Oven-heated, argon-flushed flasks were used to prepare all moisture-sensitive solutions. Activator, capping, deprotection, acidic wash and building block solutions were freshly prepared and kept under argon during the automation run. All yields of products obtained by AGA were calculated on the basis of resin loading. Resin loading was determined following previously established procedures.[13]

3.2 Preparation of stock solutions

- **Building block solution**: Between 0.06 and 0.10 mmol of building block (depending on the BB, see Module C1 and C2) was dissolved in DCM (1 mL).
- **NIS/TfOH activator solution**: 1.35 g (6.0 mmol) of recrystallized NIS was dissolved in 40 mL of a 2:1 v/v mixture of anhydrous DCM and anhydrous dioxane. Then triflic acid (55 µL, 0.6 mmol) was added. The solution is kept at 0 °C (ice bath) for the duration of the automation run.
- **Fmoc deprotection solution**: A solution of $20\%_{v/v}$ piperidine in DMF was prepared.
- **Lev deprotection solution**: Hydrazine acetate (550 mg, 5.97 mmol) was dissolved in pyridine/AcOH/H_2O (40mL, v/v, 32:8:2) and sonicated for 10 min.
- **TMSOTf solution**: TMSOTf (0.45 mL, 2.49 mmol) was added to DCM (40 mL).
- **Capping solution**: A solution of $10\%_{v/v}$ acetic anhydride and $2\%_{v/v}$ methanesulfunic acid in DCM was prepared.

3.3 Modules for automated synthesis

3.3.1 Module A: Resin preparation

All automated syntheses were performed on 0.0125 mmol scale. Resin (**L**) is placed in the reaction vessel and swollen in DCM for 20 min at room temperature prior to the synthesis. During this time, all reagent lines needed for the synthesis are washed and primed. After the swelling, the resin is washed with DMF, THF, and DCM (three times each with 2 mL for 25 s).

3.3.2 Module B: Acidic wash with TMSOTf solution (20 min)

The resin is swollen in 2 mL DCM and the temperature of the reaction vessel adjusted to -20 °C. Upon reaching the low temperature, TMSOTf solution (1 mL) is added dropwise to the reaction vessel. After bubbling for 3 min, the acidic solution is drained and the resin washed with 2 mL DCM for 25 s.

Action	Cycles	Solution	Amount	T (°C)	Incubation time
**Cooling**	-	-	-	-20	(15 min)*
**Deliver**	1	DCM	2 mL	-20	-
**Deliver**	1	TMSOTf solution	1 mL	-20	3 min
**Wash**	1	DCM	2 mL	-20	25 sec

*Time required to reach the desired temperature.

3.3.3 Module C1: Thioglycoside glycosylation (35 min-55 min)

The building block solution (0.10 mmol of BB in 1 mL of DCM per glycosylation) was delivered to the reaction vessel. After the set temperature was reached, the reaction was started by dropwise addition of the NIS/TfOH activator solution (1.0 mL, excess). The glycosylation conditions (T_1, T_2, t_1, and t_2) are building block dependent and are reported in a table below. After completion of the reaction, the solution was drained and the resin was washed with DCM, DCM:dioxane (1:2, 3 mL for 20 s) and DCM (two times, each with 2 mL for 25 s). The temperature of the reaction vessel was increased to 25 °C for the next module. In case of a double cycle (C1*, *Double cycle), module C1 was repeated twice.

Action	Cycles	Solution	Amount	T (°C)	Incubation time
**Cooling**	-	-	-	T_1	-
**Deliver**	1	BB solution	1 mL	T_1	-
**Deliver**	1	NIS/TfOH activator solution	1 mL	T_1	-
**Reaction time (BB dependent)**	1	-	-	T_1 to T_2	t_1 t_2
**Wash**	1	DCM	2 mL	T_2	5 sec
**Wash**	1	DCM : Dioxane (1:2)	2 mL	T_2	20 sec
**Heating**	-	-	-	25	-
**Wash**	2	DCM	2 mL	> 0	25 sec

BB	Equiv.	t_1 (min)	T_1 (°C)	t_2 (min)	T_2 (°C)
**BB1**	6.5	5	-20	20	0
**BB2**	6.5	5	-20	20	0
**BB4**	6.5	5	-20	20	0

3.3.4 Module C2: Glycosyl phosphate glycosylation (45 min)

The building block solution (0.06 mmol of BB in 1 mL of DCM per glycosylation) is delivered to the reaction vessel. After the set temperature is reached, the reaction is started by dropwise addition of the TMSOTf solution (1.0 mL, stoichiometric). After completion of the reaction, the solution is drained and the resin washed with

DCM (six times, each with 2 mL for 25 s). The temperature of the reaction vessel is increased to 25 °C for the next module. In case of a double cycle (C2*, *Double cycle), module C2 was repeated twice.

Action	Cycles	Solution	Amount	T (°C)	Incubation time
**Cooling**	-	-	-	-30	-
**Deliver**	1	BB solution	1 mL	-30	-
**Deliver**	1	TMSOTf solution	1 mL	-30	-
**Reaction time (BB dependent)**	1	-	-	-30 to -10	5 min 40 min
**Wash**	1	DCM	2 mL	-10	5 sec
**Heating**	-	-	-	25	-
**Wash**	6	DCM	2 mL	> 0	25 sec

BB	Equiv.	t1 (min)	T1 (°C)	t2 (min)	T2 (°C)
**BB3**	5	5	-30	40	-10
**BB5**	5	5	-30	40	-10

3.3.5 Module D: Capping (30 min)

The resin is washed with DMF (two times with 2 mL for 25 s) and the temperature of the reaction vessel adjusted to 25 °C. A pyridine solution (2 mL, 10%$_{v/v}$ in DMF) is delivered into the reaction vessel. After 1 min, the reaction solution is drained and the resin washed with DCM (three times with 3 mL for 25 s). Capping solution (4 mL) is delivered into the reaction vessel. After 20 min, the reaction solution is drained and the resin washed with DCM (three times with 3 mL for 25 s).

Action	Cycles	Solution	Amount	T (°C)	Incubation time
**Heating**	-	-	-	25	(5 min)*
**Wash**	2	DMF	2 mL	25	25 sec
**Deliver**	1	10% Pyridine in DMF	2 mL	25	1 min
**Wash**	3	DCM	2 mL	25	25 sec
**Deliver**	1	Capping Solution	4 mL	25	20 min
**Wash**	3	DCM	2 mL	25	25 sec

*Time required to reach the desired temperature.

3.3.6 Module E1: Fmoc deprotection (9 min)

The resin is washed with DMF (three times with 2 mL for 25 s) and the temperature of the reaction vessel adjusted to 25 °C. Fmoc deprotection solution (2mL) is delivered to the reaction vessel and kept under Ar bubbling. After 5 min, the reaction solution is drained and the resin washed with DMF (three times with 3 mL

for 25 s) and DCM (five times each with 2 mL for 25 s). The temperature of the reaction vessel is decreased to -20 °C for the next module.

Action	Cycles	Solution	Amount	T (°C)	Incubation time
**Wash**	3	DMF	2 mL	25	25 sec
**Deliver**	1	Fmoc depr. solution	2 mL	25	5 min
**Wash**	1	DMF	2 mL		
**Cooling**	-	-	-	-20	-
**Wash**	3	DMF	2 mL	< 25	25 sec
**Wash**	5	DCM	2 mL	< 25	25 sec

3.3.7 Module E2: Lev deprotection (90 min)

The resin is washed with DCM (three times with 2 mL for 25 s). DCM (1.3 mL) is delivered to the reaction vessel and the temperature of the reaction vessel is adjusted to 25 °C. Lev deprotection solution (2mL) is delivered to the reaction vessel, kept under pulsed Ar bubbling for 30 min. This procedure is repeated twice. The reaction solution is drained and the resin washed with DMF (three times with 3 mL for 25 s) and DCM (five times each with 2 mL for 25 s).

Action	Cycles	Solution	Amount	T (°C)	Incubation time
**Wash**	3	DMF	2 mL	25	25 sec
**Deliver**	2	Lev depr. solution	2 mL	25	30 min
**Wash**	1	DMF	2 mL	-	-
**Cooling**	-	-	-	-20	-
**Wash**	3	DMF	2 mL	< 25	25 sec
**Wash**	5	DCM	2 mL	< 25	25 sec

3.4 Post-AGA manipulations

3.4.1 Module F: On-resin methanolysis

The resin is suspended in THF (4 mL). MeONa in MeOH (0.5 M, 0.4 mL) is added and the suspension is gently shaken at room temperature. After micro-cleavage (see *Module G2*) indicates the complete removal of benzoyl groups, the resin is repeatedly washed with MeOH (3 x 2 mL) and DCM (3 x 2 mL).

3.4.2 Module G1: Cleavage from solid support

The oligosaccharides are cleaved from the solid support using a continuous-flow photoreactor as described previously.[14]

3.4.3 Module G2: Micro-cleavage from solid support

Trace amount of resin (around 20 beads) is dispersed in DCM (0.1 mL) and irradiated with a UV lamp (6 W, 356 nm) for 10 minutes. ACN (10 μL) is then added to the resin and the resulting solution analyzed by MALDI.

3.4.4 Module H: Hydrogenolysis

The crude compound obtained from *Module G1* is dissolved in 2 mL of EtOAc:*t*BuOH:H_2O (2:1:1). $Pd(OH)_2/C$ (10-20%$_w$, moistened with water) is added and the reaction stirred in a pressurized reactor under H_2 pressure (4 bar). The reaction progress is monitored to avoid undesired side products formation (*i.e.* degradation of reducing end).[15] Upon completion, the reaction is filtered (PTFE 0.45 μm 25 mm syringe filter, Fisher scientific) and washed with EtOAc, H_2O, and ACN (4 mL each). The filtrates are concentrated *in vacuo*.

3.4.5 Module I: Purification

The final compounds are analyzed using analytical reversed phase HPLC (Agilent 1200 Series, Method A1). The purification of the crudes is conducted using reversed phase HPLC (Agilent 1200 Series, Method A2).

- **Method A1**: (Hypercarb column, ThermoFisher scientific, 150 x 4.6 mm, 3 μm) flow rate of 0.7 mL/min with H_2O (0.1% formic acid) and ACN as eluents [isocratic (5 min), linear gradient to 60% ACN (30 min), linear gradient to 100% ACN (5 min), isocratic 100% ACN (5 min)].

- **Method A2 (Prep)**: (Hypercarb column, ThermoFisher scientific, 150 x 10 mm, 5 μm), flow rate of 3 mL /min with H_2O (0.1% formic acid) and ACN as eluents [isocratic (5 min), linear gradient to 60% ACN (30 min), linear gradient to 100% ACN (5 min), isocratic 100% ACN (5 min)].

- **Method A3 (Prep)**: (Hypercarb column, ThermoFisher scientific, 150 x 10 mm, 5 μm), flow rate of 3 mL /min with H_2O (0.1% formic acid) and ACN as eluents [isocratic (5 min), linear gradient to 25% ACN (60 min), linear gradient to 100% ACN (5 min), isocratic 100% ACN (5 min)].

Following final purification, all deprotected products are lyophilized on a Christ Alpha 2-4 LD plus freeze dryer prior to characterization.

3.5 Oligosaccharides synthesis

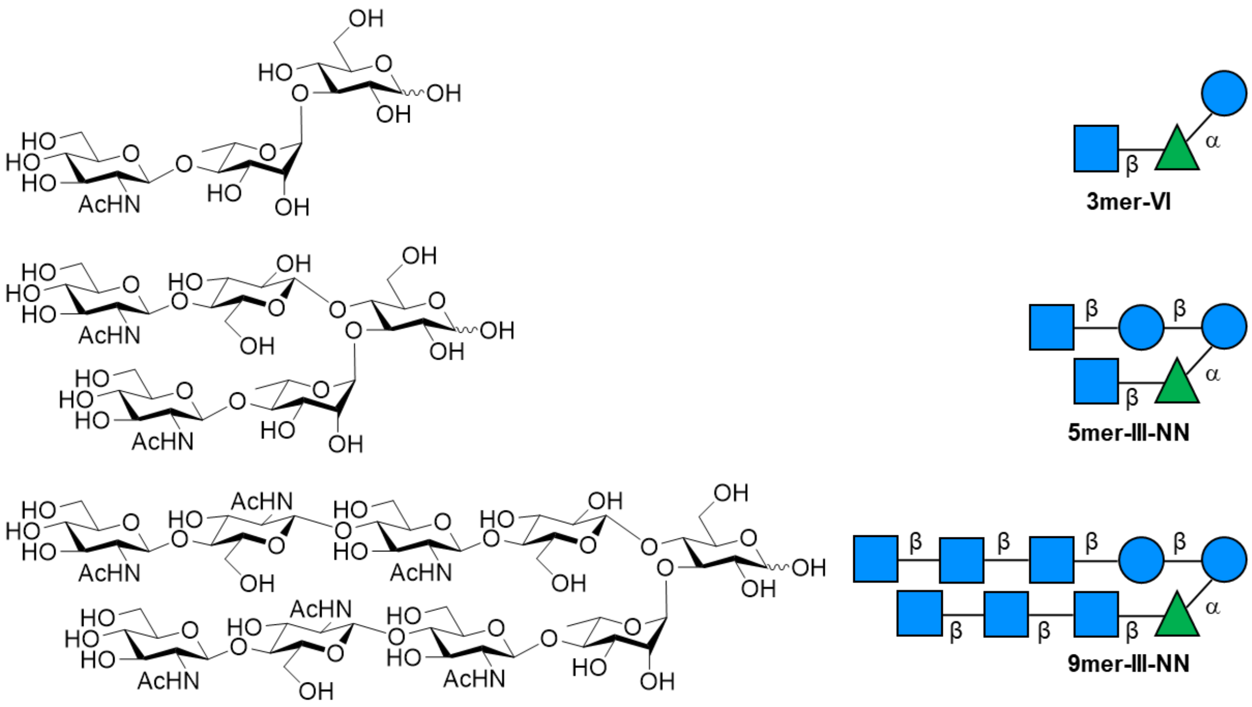

Figure S25 Oligosaccharides synthesized by AGA in this work.

3.5.1 Synthesis of 3mer-VI

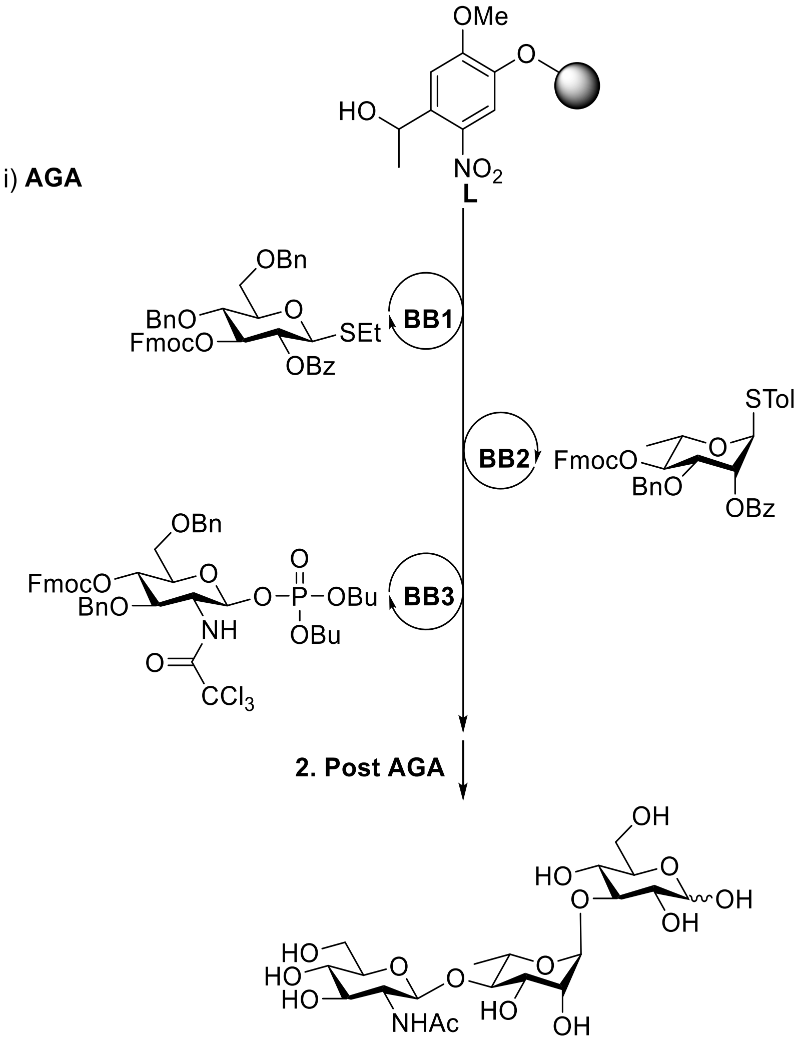

3mer-VI

Step	BB	Modules	Notes
AGA	-	**A**	**L** swelling
	**BB1**	**B**, **C1**, **D**, **E1**	**C1**: (**BB1**, -20 °C for 5 min, 0 °C for 20 min)
	**BB2**	**B**, **C1**, **D**, **E1**	**C1**: (**BB2**, -20 °C for 5 min, 0 °C for 20 min)
	**BB3**	(**B**, **C2**, **D**, **E1**)	**C2**: (**BB3**, -30 °C for 5 min, -10 °C for 40 min)
Post-AGA	-	**F**, **G1**, **H**, **I**	**F**: (3.5 h)<br>**H**: (8 h)<br>**I**: (Method A2: 16.6, 17.0 min)

Automated synthesis, global deprotection, and purification afforded **3mer-VI** as a white solid (1.4 mg, 21% overall yield).

^{1}H NMR (700 MHz, D_2O) δ 5.15 (d, *J* = 3.7 Hz, 0.4H, H-1α Glc), 5.06 (d, *J* = 1.9 Hz, 0.6H, H-1β Rha), 5.03 (d, *J* = 1.9 Hz, 0.4H, H-1α Rha), 4.72 (d, *J* = 8.5 Hz, 1H, H-1 Glc), 4.60 (d, *J* = 8.1 Hz, 0.6H, H-1β Glc), 4.03 – 3.97 (m, 1H), 3.94 (dd, *J* = 3.4, 1.8 Hz, 1H), 3.89 – 3.77 (m, 4H), 3.73 – 3.70 (m, 2H), 3.68 – 3.63 (m, 2H), 3.58 – 3.49 (m, 3H), 3.43 – 3.36 (m, 4H), 3.29 (dd, *J* = 9.4, 8.1 Hz, 1H), 2.00 (s, 2H), 1.25 (dd, *J* = 6.3, 3.4 Hz, 3H, CH_3-6 Rha). ^{13}C NMR (176 MHz, D_2O) δ 174.72 (C=O GlcNAc), 101.70 (C-1 Glc), 100.81 (C-1α Rha), 100.74 (C-1β Rha), 95.55 (C-1β Glc), 92.08 (C-1α Glc), 82.22, 80.27, 79.75, 75.82, 75.55, 74.60, 73.81, 71.86, 71.41, 70.40, 70.32, 69.69, 67.90, 67.07 (C-5 Rha), 60.62, 60.43, 55.81, 22.15, 16.79 (C-6 Rha). ESI-HRMS m/z 552.1918 $[M+Na]^+$ ($C_{20}H_{35}NNaO_{15}$ requires 552.1909).

RP-HPLC of 3mer-VI (ELSD trace, Method A1, t_R = 15.9, 16.4 min)

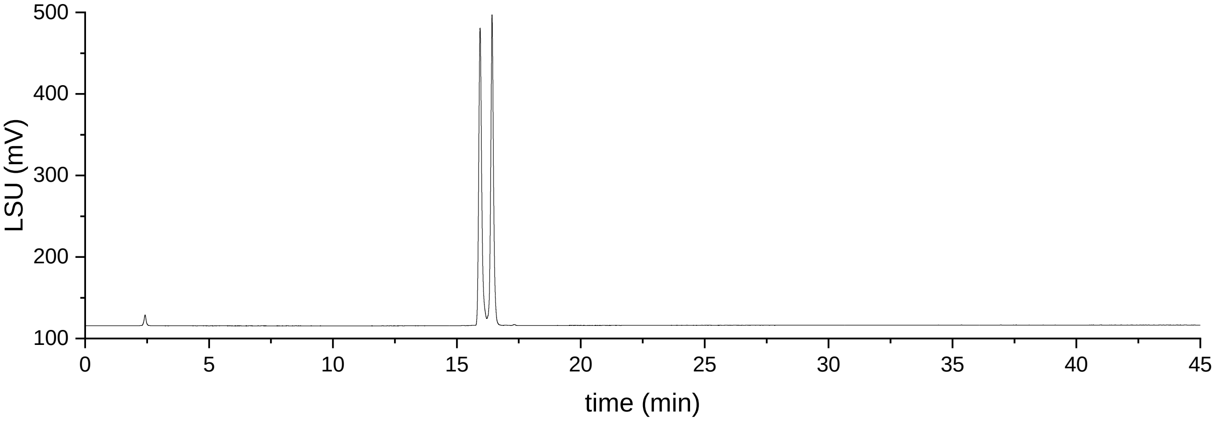

^{1}H NMR of 3mer-VI (700 MHz, D_2O)

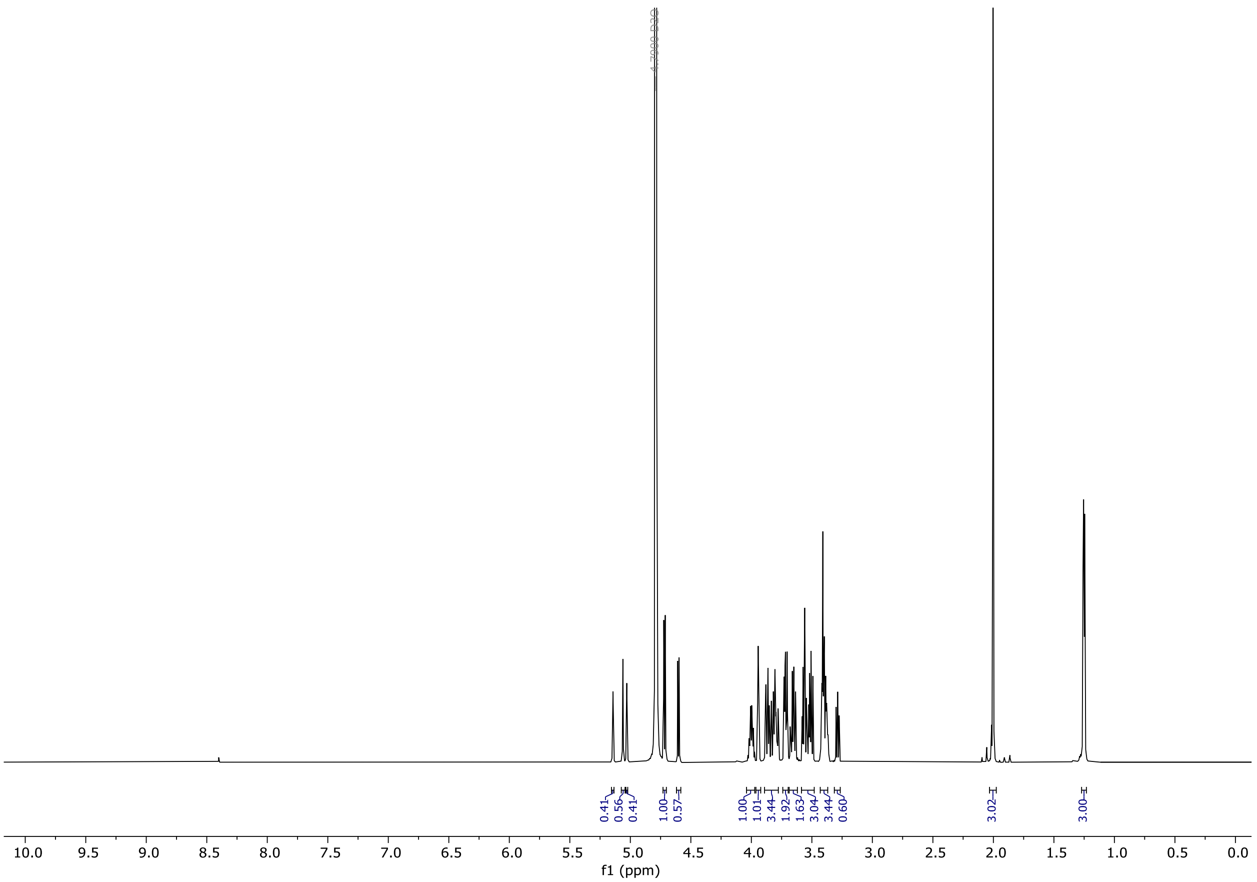

^{13}C NMR of 3mer-VI (176 MHz, D_2O)

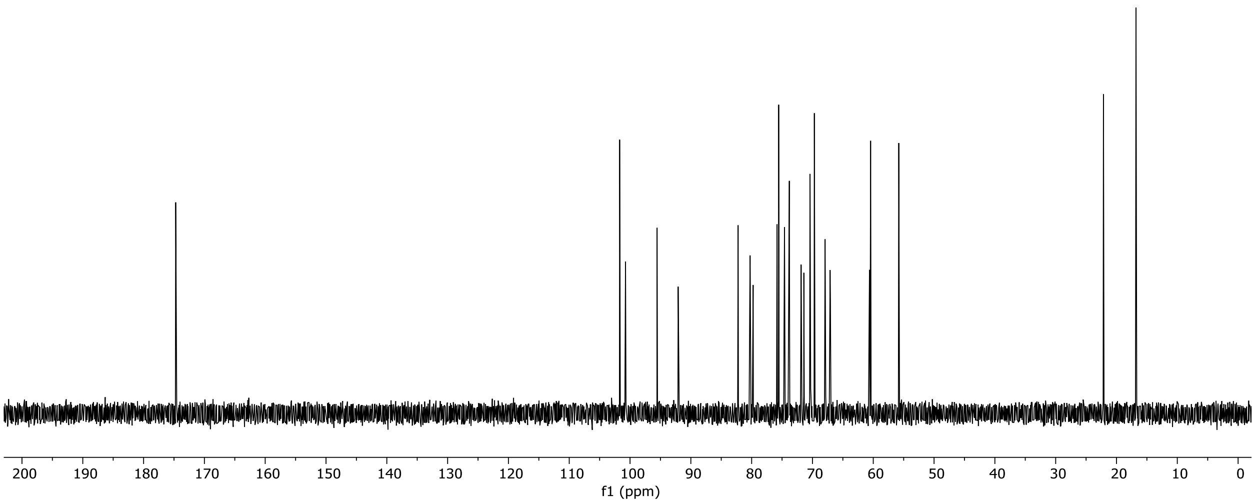

COSY NMR of 3mer-VI (D_2O)

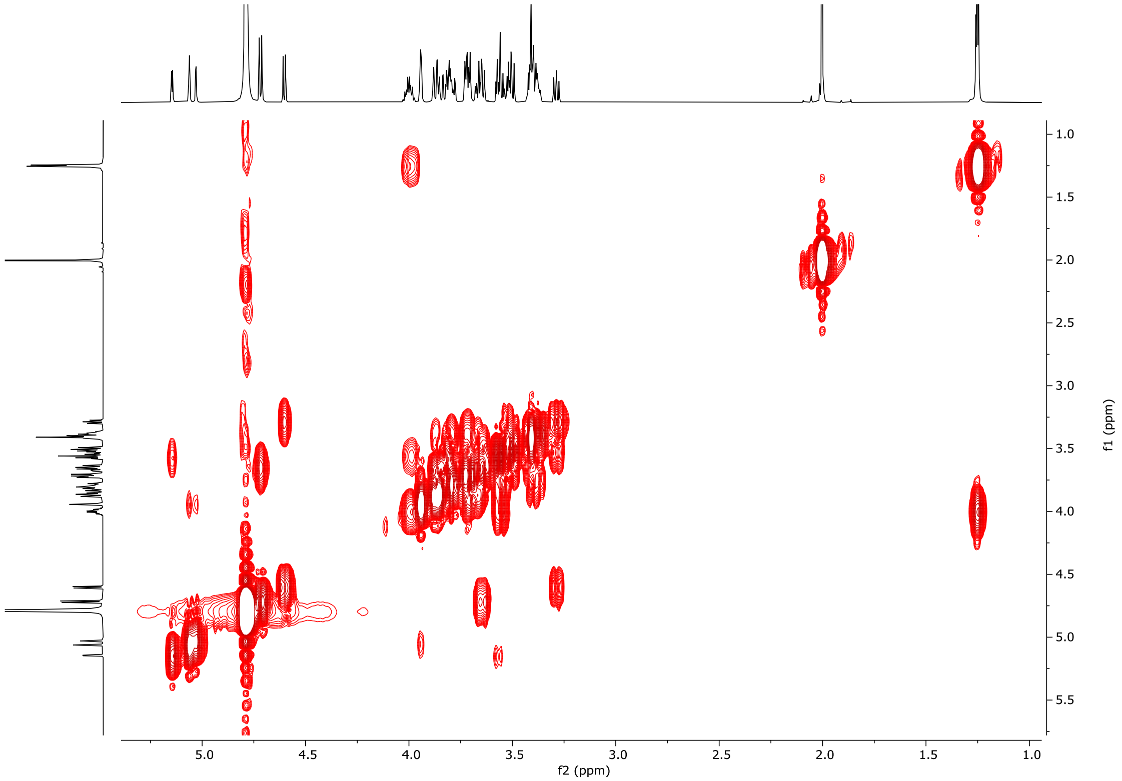

HSQC NMR of 3mer-VI (D_2O)

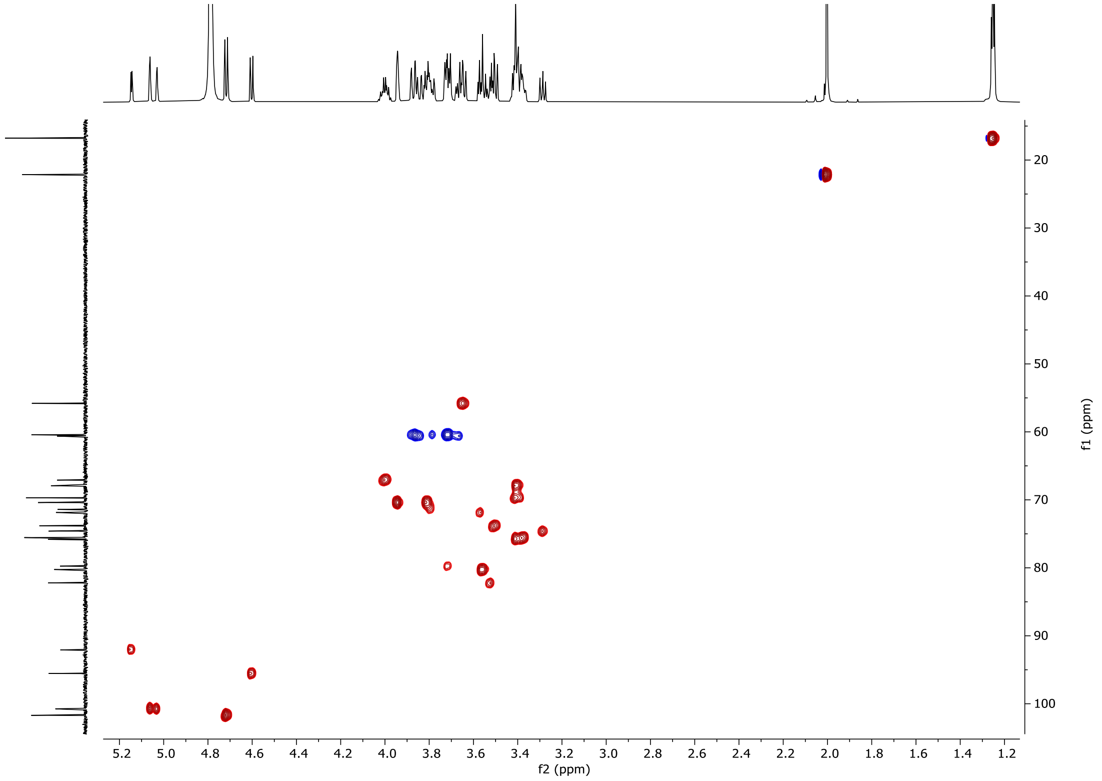

3.5.2 Synthesis of 5mer-III-NN

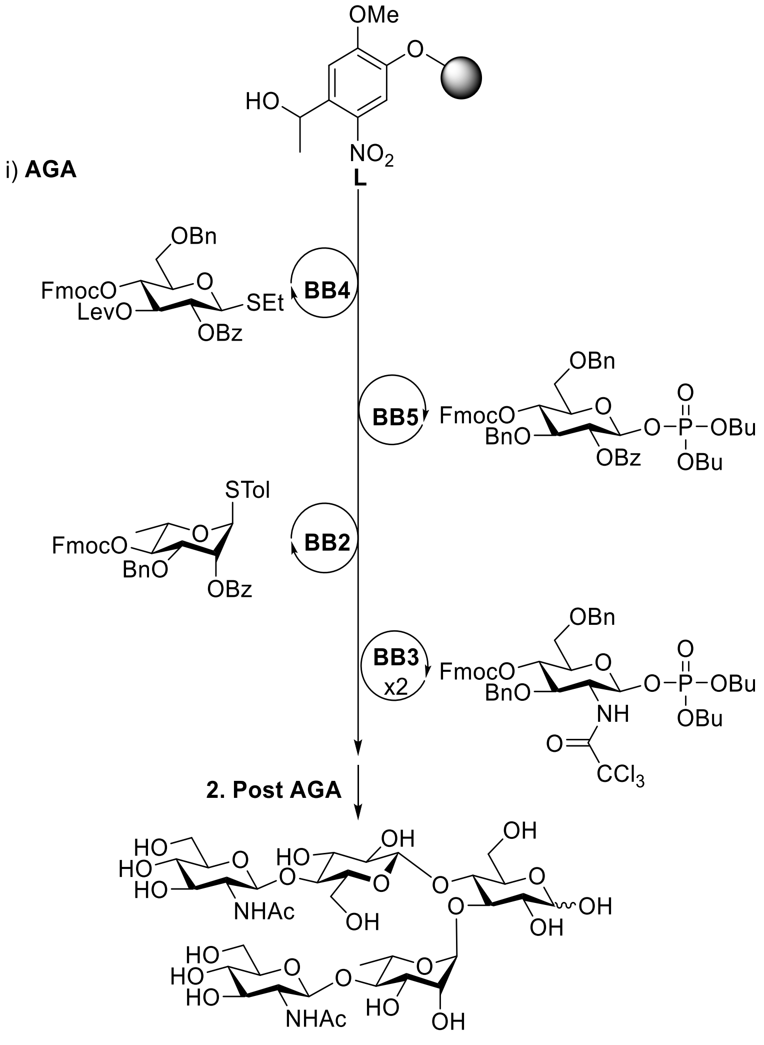

Step	BB	Modules	Notes
AGA	-	**A**	**L** swelling
	**BB4**	**B**, **C1**, **D**, **E1**	**C1**: (**BB4**, -20 °C for 5 min, 0 °C for 20 min)
	**BB5**	**B**, **C2***, **D** **E2**	**C2***: (**BB5**, -30 °C for 5 min, -10 °C for 40 min) *Double cycle
	**BB2**	**B**, **C1**, **D**, **E1**	**C1**: (**BB2**, -20 °C for 5 min, 0 °C for 20 min)
	(**BB3**)x2	(**B**, **C2***)x2 **D**, **E1**	**C2***: (**BB3**, -30 °C for 5 min, -10 °C for 40 min) *Double cycle
Post-AGA	-	**F, G1**, **H**, **I**	**F**: (5 h) **H**: (16 h) **I**: (Method A2: 20.0, 20.4 min)

Automated synthesis, global deprotection, and purification afforded **5mer-III-NN** as a white solid (2.6 mg, 23% overall yield).

^{1}H NMR (700 MHz, D_2O) δ 5.14 (d, *J* = 4.0 Hz, 1H, H-1α Glc, H-1β Rha), 5.09 (d, *J* = 1.7 Hz, 0.4 H, H-1α Rha), 4.63 (d, *J* = 8.5 Hz, 1H, H-1 GlcNAc), 4.60 (d, *J* = 8.0 Hz, 0.6H, H-1β Glc), 4.54 (d, *J* = 8.4 Hz, 1H, H-1 GlcNAc), 4.44 (d, *J* = 7.9 Hz, 1H, H-1 Glc), 4.39 (td, *J* = 9.6, 6.2 Hz, 1H, H-5 Rha), 3.97 (dd, *J* = 3.6, 1.7 Hz, 1H), 3.89 – 3.86 (m, 3.4H), 3.83 – 3.80 (m, 2H), 3.77 – 3.68 (m, 6H), 3.66 (dd, *J* = 10.2, 8.5 Hz, 1H), 3.61 – 3.54 (m, 3H), 3.54 – 3.45 (m, 5H), 3.44 – 3.37 (m, 3H), 3.30 (dd, *J* = 9.5, 7.9 Hz, 0.6H), 3.27 – 3.22 (m, 2H), 2.05 (d, *J* = 10.2 Hz, 6H), 1.28 (dd, *J* = 6.3, 3.8 Hz, 3H, C-6 Rha). ^{13}C NMR (176 MHz, D_2O) δ 174.51 (C=O GlcNAc), 174.06 (C=O GlcNAc), 102.63 (C-1 GlcNAc), 101.41 (C-1 GlcNAc), 101.03 (C-1α Rha), 100.97 (C-1β Rha), 100.79 (C-1 Glc), 95.71 (C-1 β Glc), 91.95 (C-1 α Glc), 81.98, 81.90, 80.13, 79.22, 76.86, 75.80, 75.72, 75.33, 75.20, 75.13, 74.85, 74.82, 74.00, 73.29, 73.01, 72.40, 72.27, 70.81, 70.22, 70.18, 70.14, 69.68, 69.48, 67.05 61.41, 60.58, 60.31, 59.45, 59.36, 56.07, 55.42, 22.38 (CH_3 GlcNAc), 22.11 (CH_3 GlcNAc), 17.00 (C-6 Rha). ESI-HRMS m/z 917.3251 $[M+Na]^+$ ($C_{34}H_{58}N_2NaO_{25}$ requires 917.3226).

RP-HPLC of 5mer-III-NN (ELSD trace, Method A1, t_R = 19.3, 19.7 min)

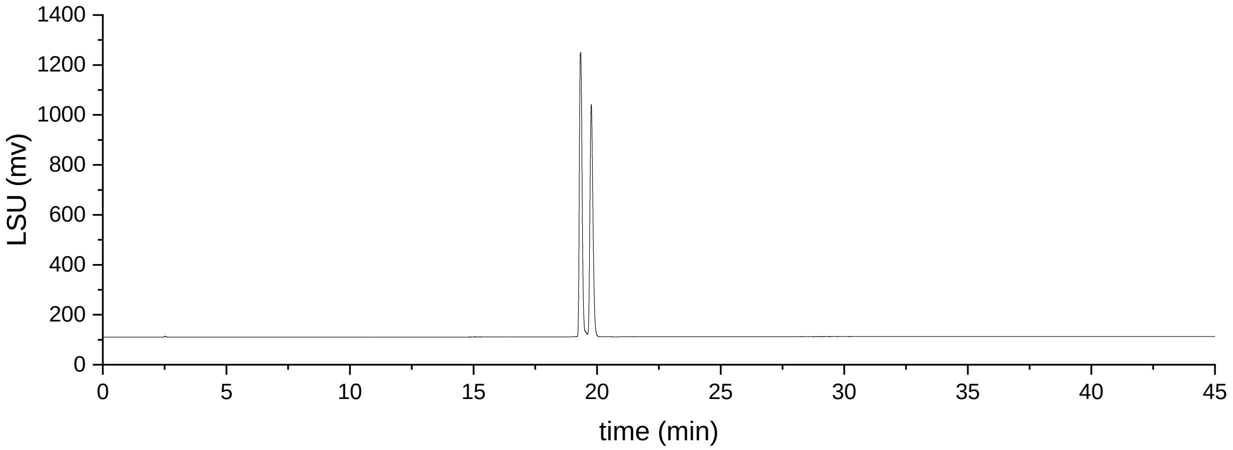

^{1}H NMR of 5mer-III-NN (700 MHz, D_2O)

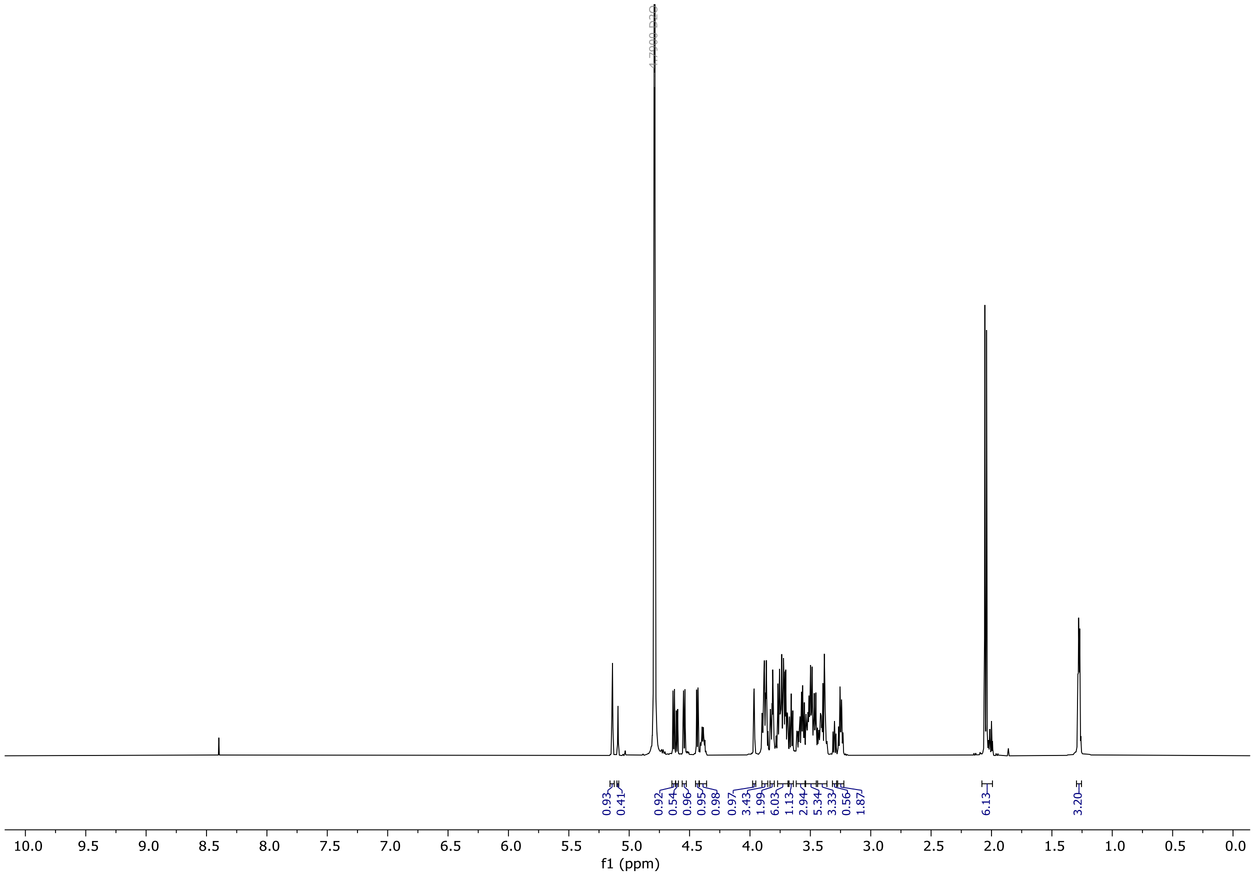

^{13}C NMR of 5mer-III-NN (176 MHz, D_2O)

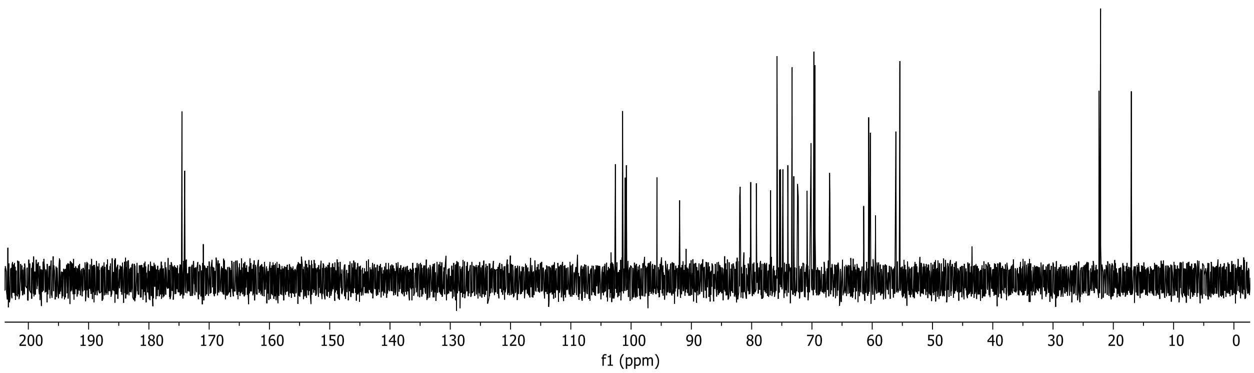

COSY NMR of 5mer-III-NN (D_2O)

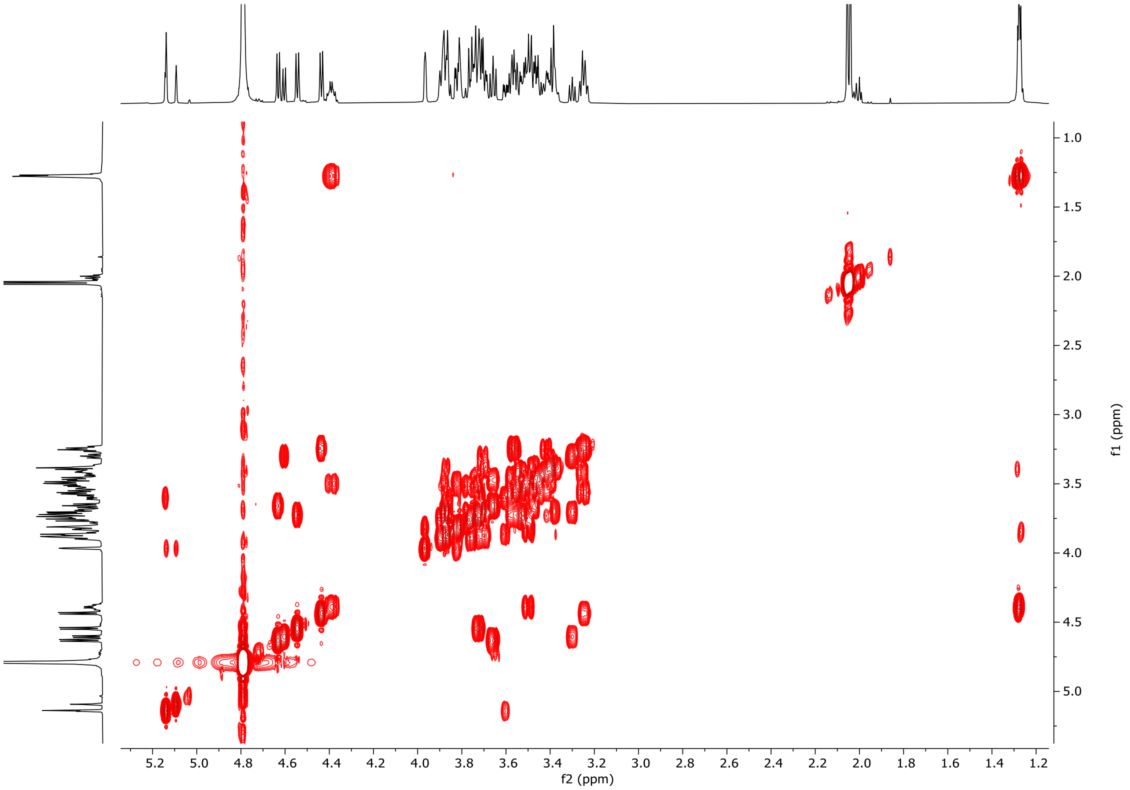

HSQC NMR of 5mer-III-NN (D_2O)

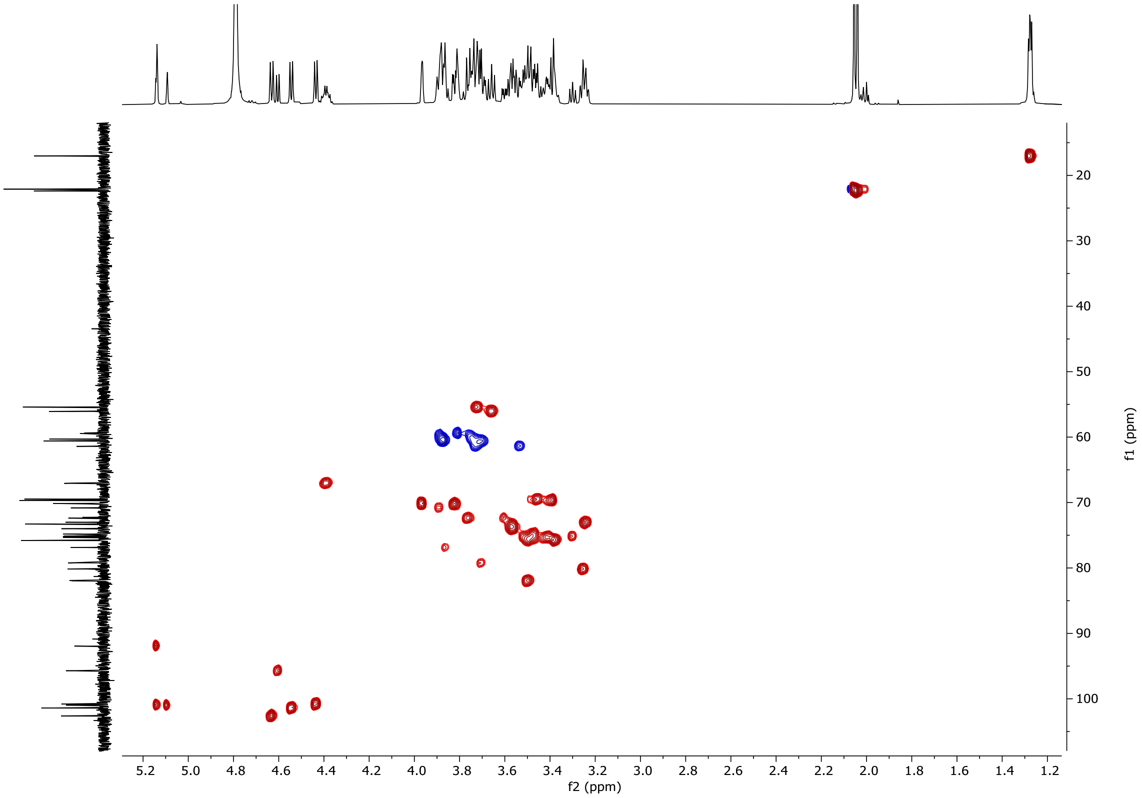

3.5.3 Synthesis of 9mer-III-NN

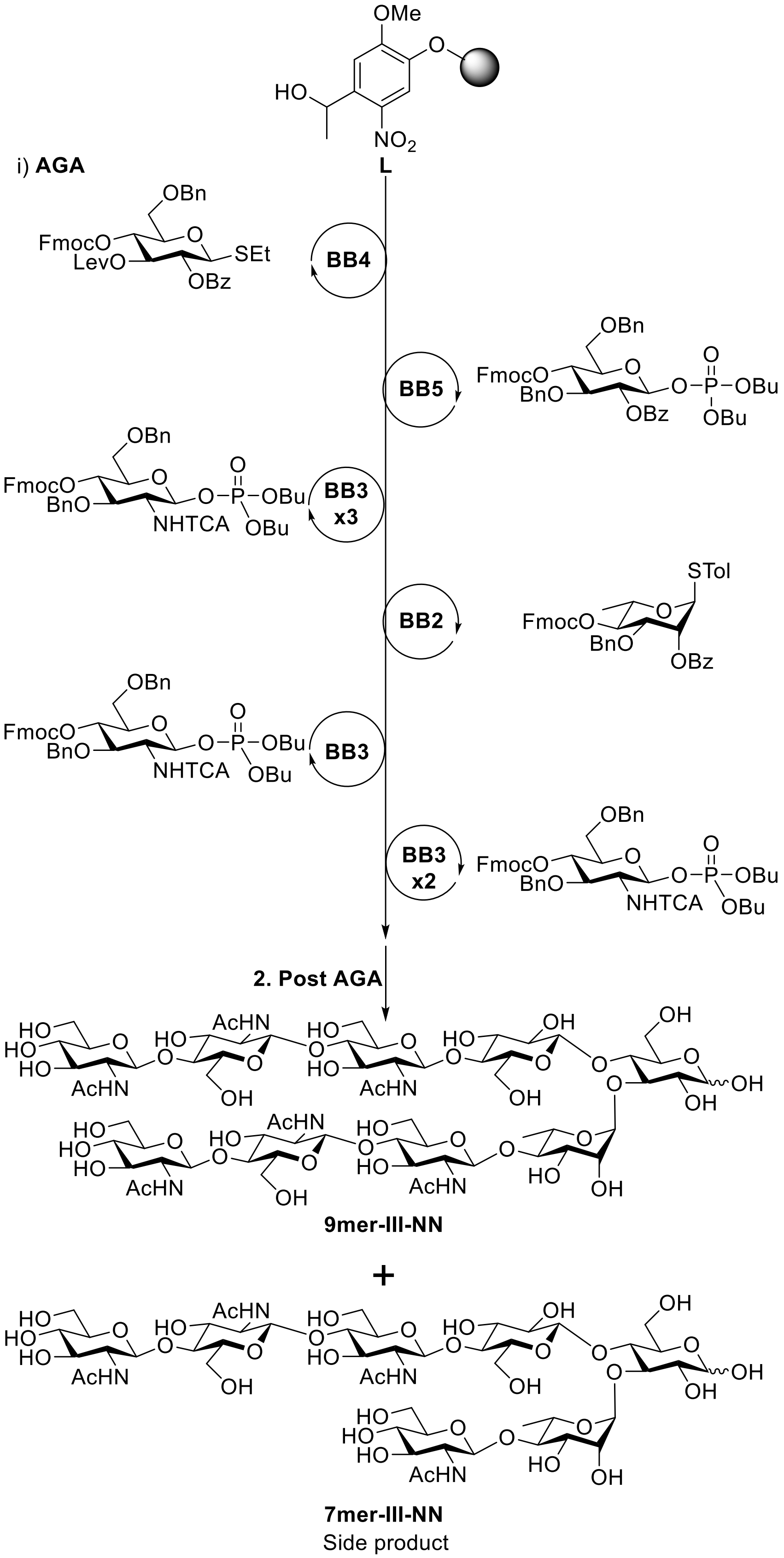

7mer-III-NN
Side product

Step	BB	Modules	Notes
AGA	-	**A**	**L** swelling
	**BB4**	**B**, **C1**, **D**, **E1**	**C1**: (**BB4**, -20 °C for 5 min, 0 °C for 20 min)
	**BB5**	**B**, **C2***, **D**, **E1**	**C2***: (**BB5**, -30 °C for 5 min, -10 °C for 40 min) *Double cycle
	(**BB3**)x3	(**B**, **C2**, **D**, **E1**)x3 **D**, **E2**	**C2**: (**BB3**, -30 °C for 5 min, -10 °C for 40 min)
	**BB2**	**B**, **C1**, **D**, **E1**	**C1**: (**BB4**, -20 °C for 5 min, 0 °C for 20 min)
	**BB3**	(**B**, **C2***, **D**, **E1**)	**C2***: (**BB3**, -30 °C for 5 min, -10 °C for 40 min) *Double cycle
	(**BB3**)x2	(**B**, **C2**, **D**, **E1**)x2	**C2**: (**BB3**, -30 °C for 5 min, -10 °C for 40 min)
Post-AGA	-	**F, G1**, **H**, **I**	**F**: (5 h) **H**: (24 h) **I**: (Method A3: 53.5, 54.1 min)

Automated synthesis, global deprotection, and purification afforded **9mer-III-NN** as a white solid (0.8 mg 4% overall yield). The side product **7mer-III-NN** (1.9 mg, 12 % overall yield), resulting from the incomplete glycosylation between GlcNAc (solid-bound) and GlcNAc (**BB3**) on the bottom strand, was also isolated.

^{1}H NMR (700 MHz, D_2O) δ 5.08 (d, *J* = 3.8 Hz, 0.4H, H-1α Glc), 5.06 (s, 0.6H, H-1β Rha), 5.02 (s, 0.4H, H-1α Rha), 4.57 (d, *J* = 8.6 Hz, 1H, H-1 GlcNAc), 4.54 (d, *J* = 7.9 Hz, 0.6H, H-1β Glc), 4.48 (t, *J* = 7.8 Hz, 5H, H-1 GlcNAc), 4.37 (d, *J* = 7.9 Hz, 1H, H-1 Glc), 4.28 (t, *J* = 8.0 Hz, 1H, H-5 Rha), 3.90 (s, 1H), 3.81 (d, *J* = 12.6 Hz, 3.4H), 3.75 (t, *J* = 10.1 Hz, 7H), 3.70 – 3.59 (m, 14H), 3.54 (tq, *J* = 9.5, 5.2 Hz, 9H), 3.46 (td, *J* = 15.9, 7.7 Hz, 8H), 3.37 (dq, *J* = 18.1, 7.8 Hz, 6.6H), 3.20 (dt, *J* = 31.4, 8.6 Hz, 2H), 2.00 – 1.91 (m, 18H), 1.19 (t, *J* = 4.9 Hz, 3H). ^{13}C NMR was not recorded due to low solubility of **9mer-III-NN** in D_2O. ESI-HRMS m/z 854.3292 $[M+2H]^{2+}$ ($C_{66}H_{110}N_6O_{45}$ requires 854.3330).

RP-HPLC of 9mer-III-NN (ELSD trace, Method A1, t_R = 21.9, 22.1 min)

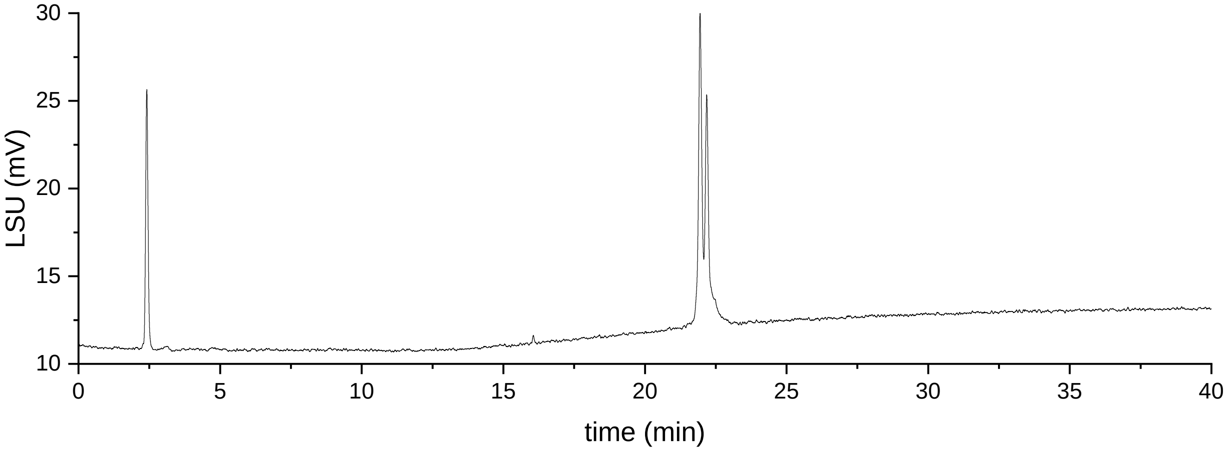

^{1}H NMR of 9mer-III-NN (700 MHz, D_2O)

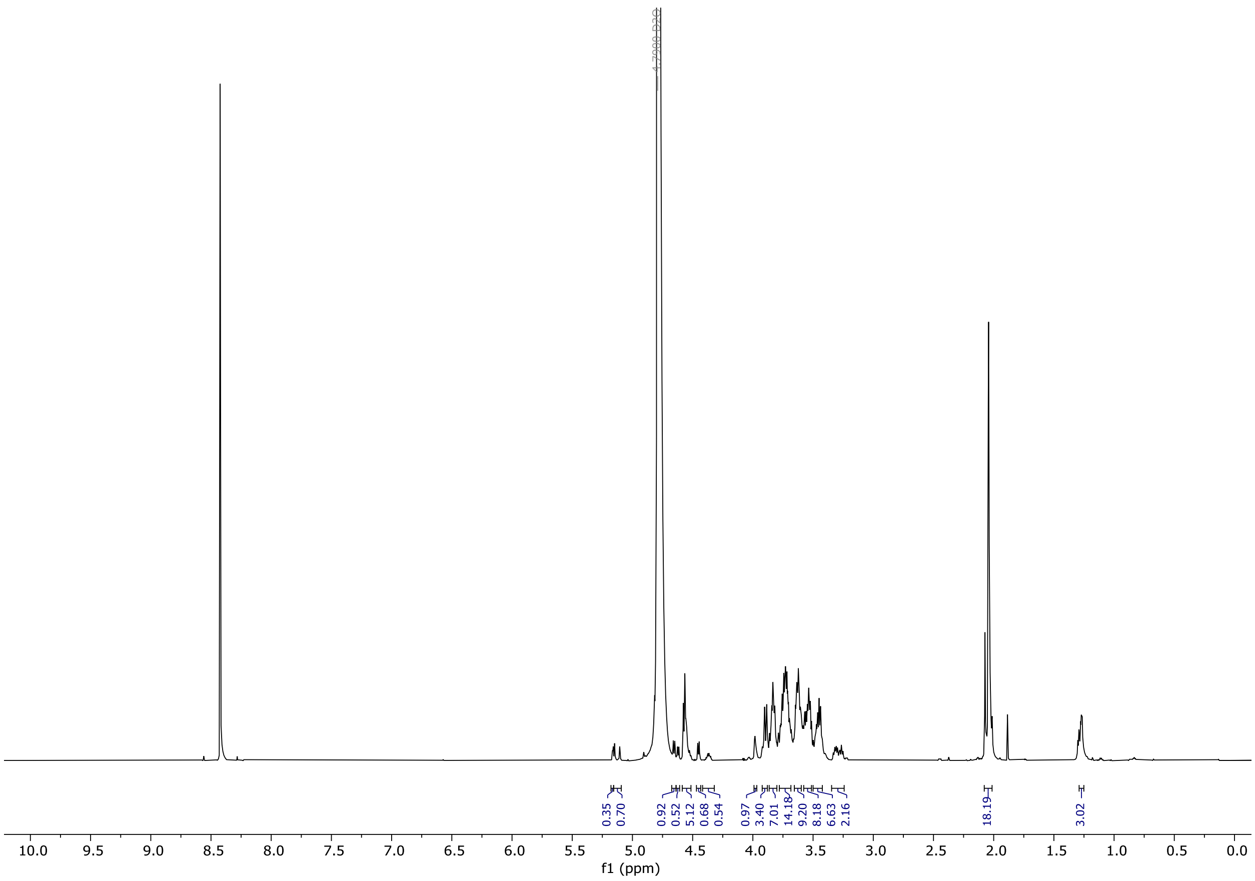

COSY NMR of 9mer-III-NN (D_2O)

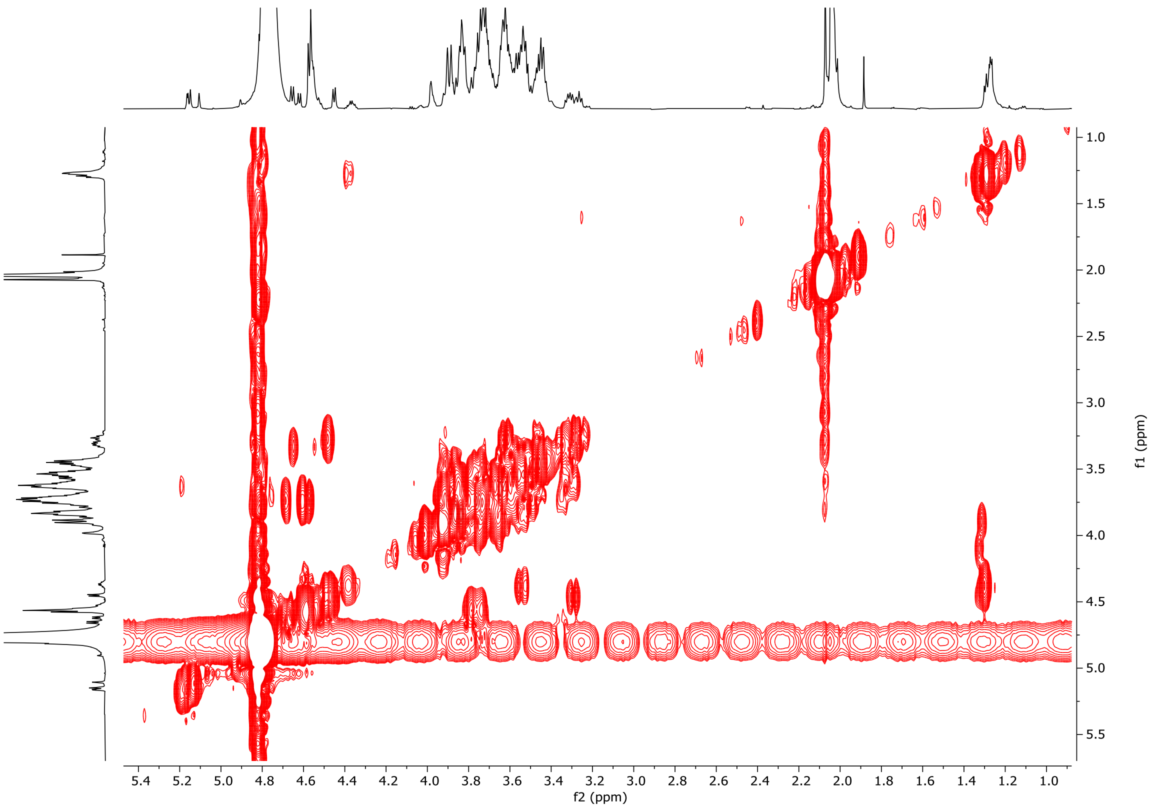

HSQC NMR of 9mer-III-NN (D_2O)

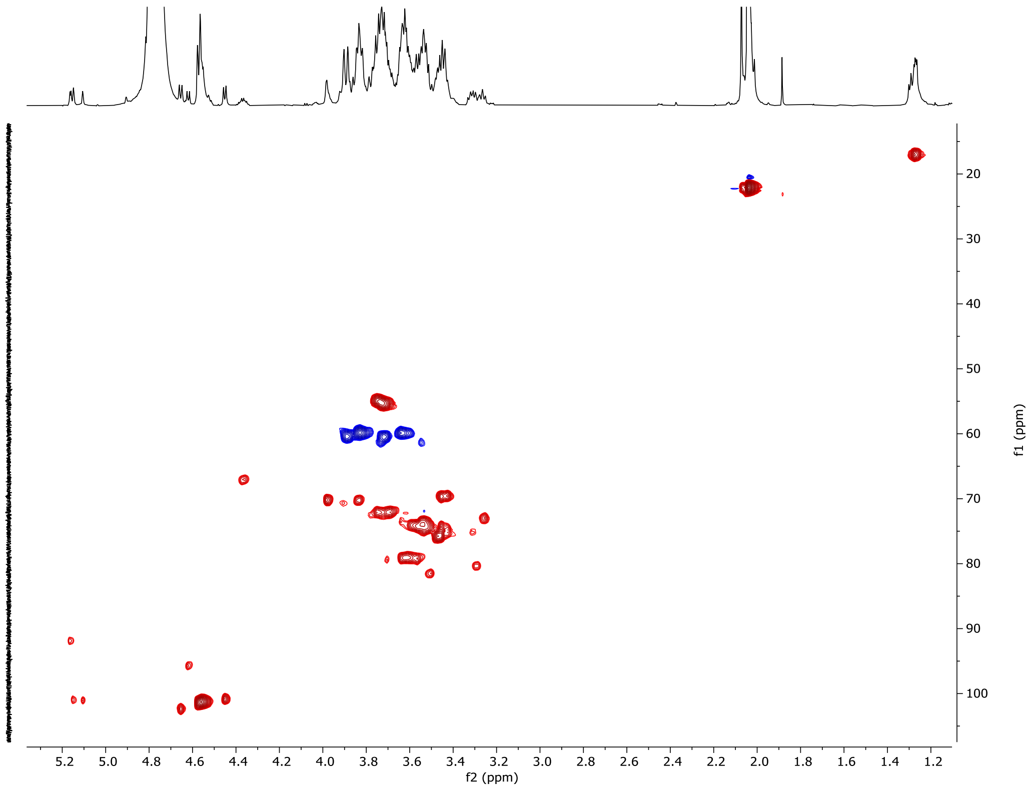

Characterization of 7mer-III-NN

^{1}H NMR (700 MHz, D_2O) δ 5.16 (t, *J* = 2.7 Hz, 1H, H-1α Glc, H-1β Rha), 5.12 (d, *J* = 1.7 Hz, 0.4H, H-1α Rha), 4.67 – 4.65 (m, 1H, H-1 GlcNAc), 4.63 (d, *J* = 8.0 Hz, 0.6H, H-1β Glc), 4.56 (dd, *J* = 10.5, 8.5 Hz, 3H, H-1 GlcNAc), 4.45 (d, *J* = 7.9 Hz, 1H, H-1 Glc), 4.41 (tq, *J* = 9.5, 6.2 Hz, 1H, H-5 Rha), 3.99 (dd, *J* = 3.5, 1.7 Hz, 1H), 3.93 – 3.87 (m, 3.4H), 3.83 (ddd, *J* = 13.8, 10.6, 2.6 Hz, 4H), 3.79 – 3.68 (m, 10H), 3.68 – 3.60 (m, 6H), 3.60 – 3.50 (m, 7H), 3.50 – 3.42 (m, 4H), 3.41 – 3.38 (m, 1.4H), 3.34 – 3.31 (m, 0.6H), 3.31 – 3.23 (m, 1.6H), 2.08 – 2.02 (m, 12H), 1.29 (dd, *J* = 6.3, 3.7 Hz, 3H, C-6 Rha). ^{13}C NMR (176 MHz, D_2O) δ 174.57 (C=O GlcNAc),174.55 (C=O GlcNAc), 174.50 (C=O GlcNAc), 174.09 (C=O GlcNAc), 102.61(C-1 GlcNAc), 101.39 (C-1 GlcNAc), 101.30 (C-1 GlcNAc), 101.20 (C-1 GlcNAc), 101.05 (C-1α Rha), 100.82 (C-1β Rha), 100.83 (C-1 Glc), 95.73 (C-1 β Glc), 91.97 (C-1 α Glc), 81.87, 80.18, 79.27, 78.99, 78.84, 76.91, 75.83, 75.78, 75.34, 75.24, 75.15, 74.84, 74.44, 74.41, 73.98, 73.36, 73.03, 72.12, 71.97, 70.85, 70.18, 69.80, 69.61, 67.04, 60.67, 60.45, 59.89, 59.79, 59.50, 56.04, 55.50, 54.95, 54.90, 22.42 (CH_3 GlcNAc), 22.15 (CH_3 GlcNAc), 22.08 (CH_3 GlcNAc), 22.03 (CH_3 GlcNAc), 17.02 (C-6 Rha). ESI-HRMS m/z 1301.503 $[M+Na]^+$ ($C_{50}H_{85}N_4O_{35}$ requires 1301.499).

^{1}H NMR of 7mer-III-NN (700 MHz, D_2O)

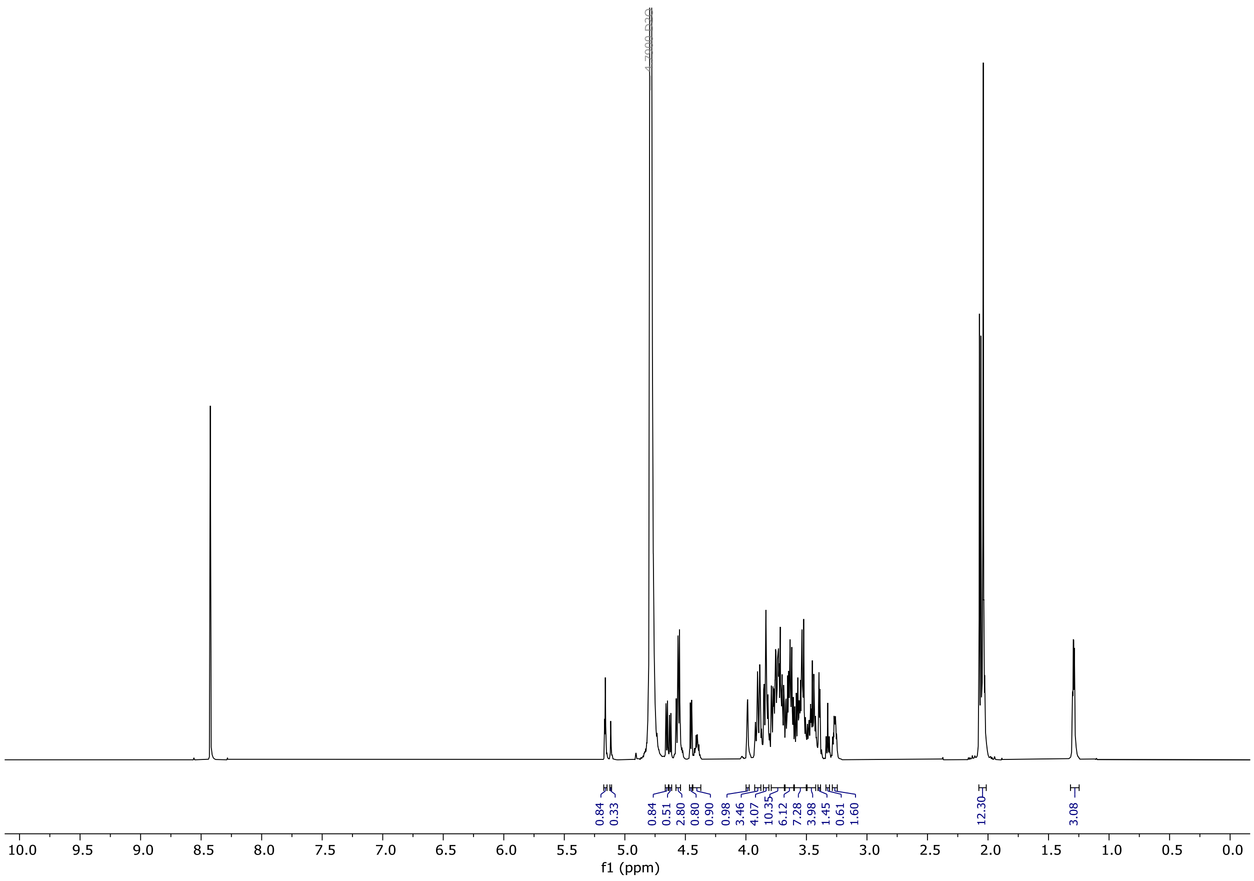

^{13}C NMR of 7mer-III-NN (700 MHz, D_2O)

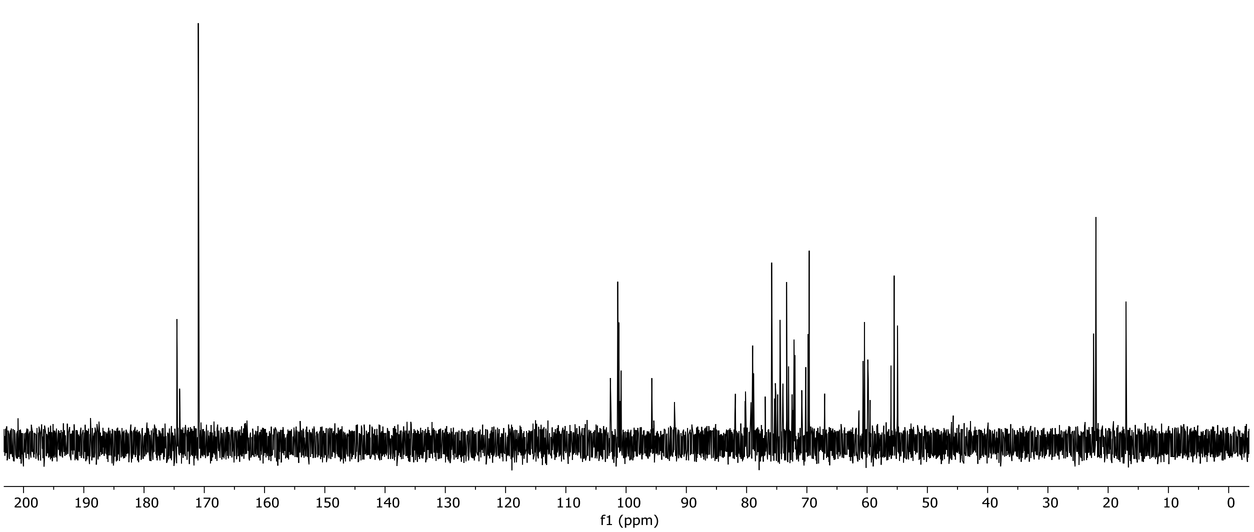

COSY NMR of 7mer-III-NN (D_2O)

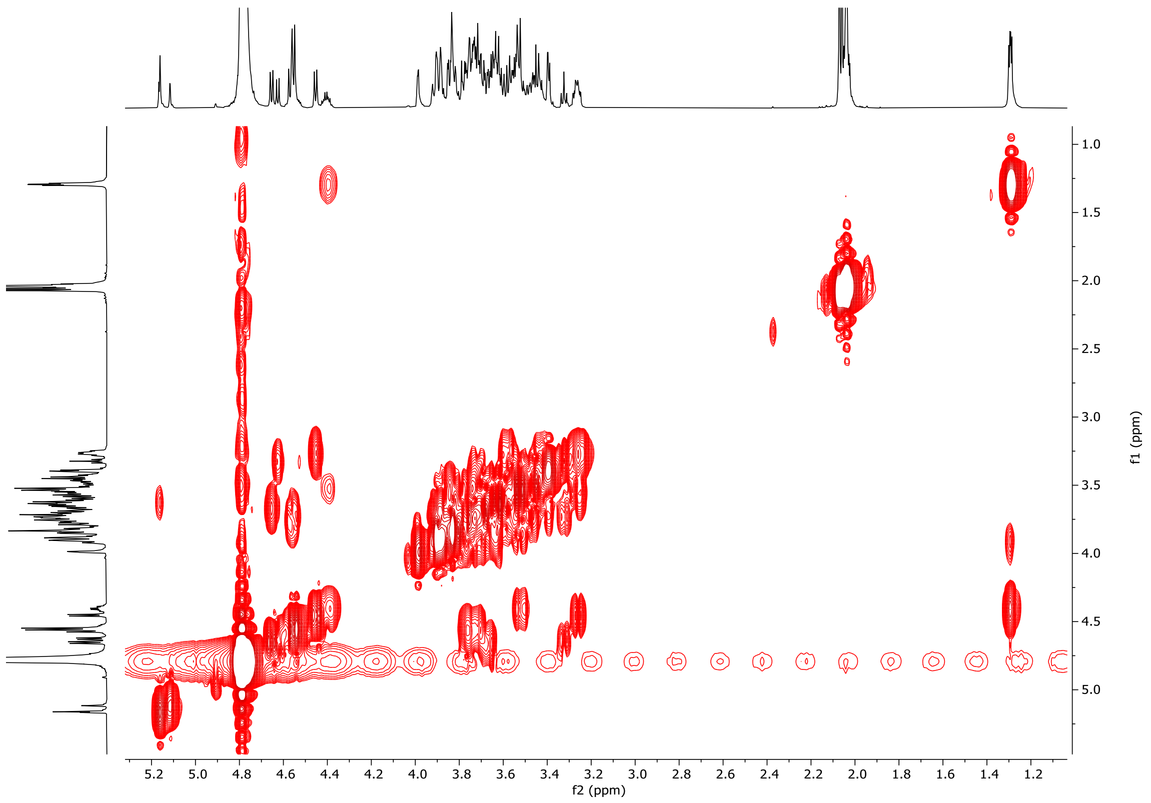

HSQC NMR of 7mer-III-NN (D_2O)

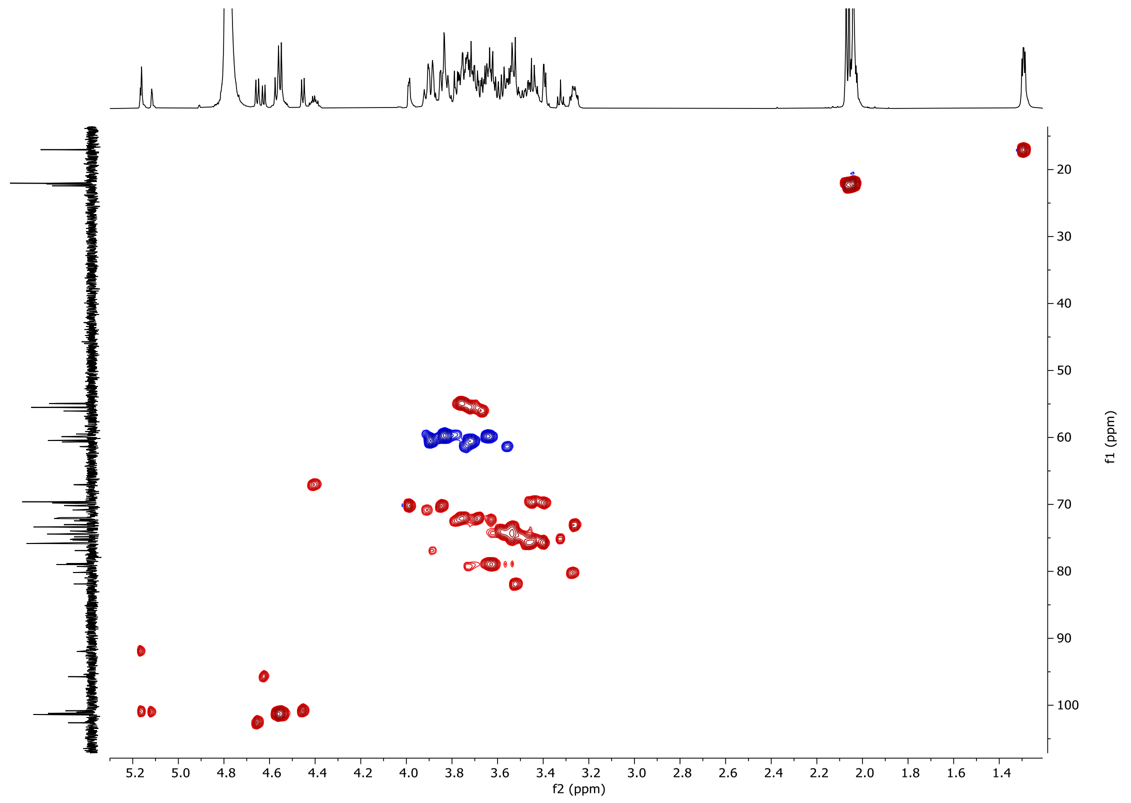

4 NMR analysis

4.1 NMR characterization of 5mer-III-NN

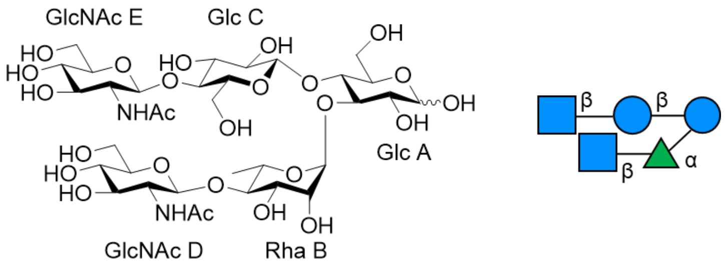

5mer-III-NN

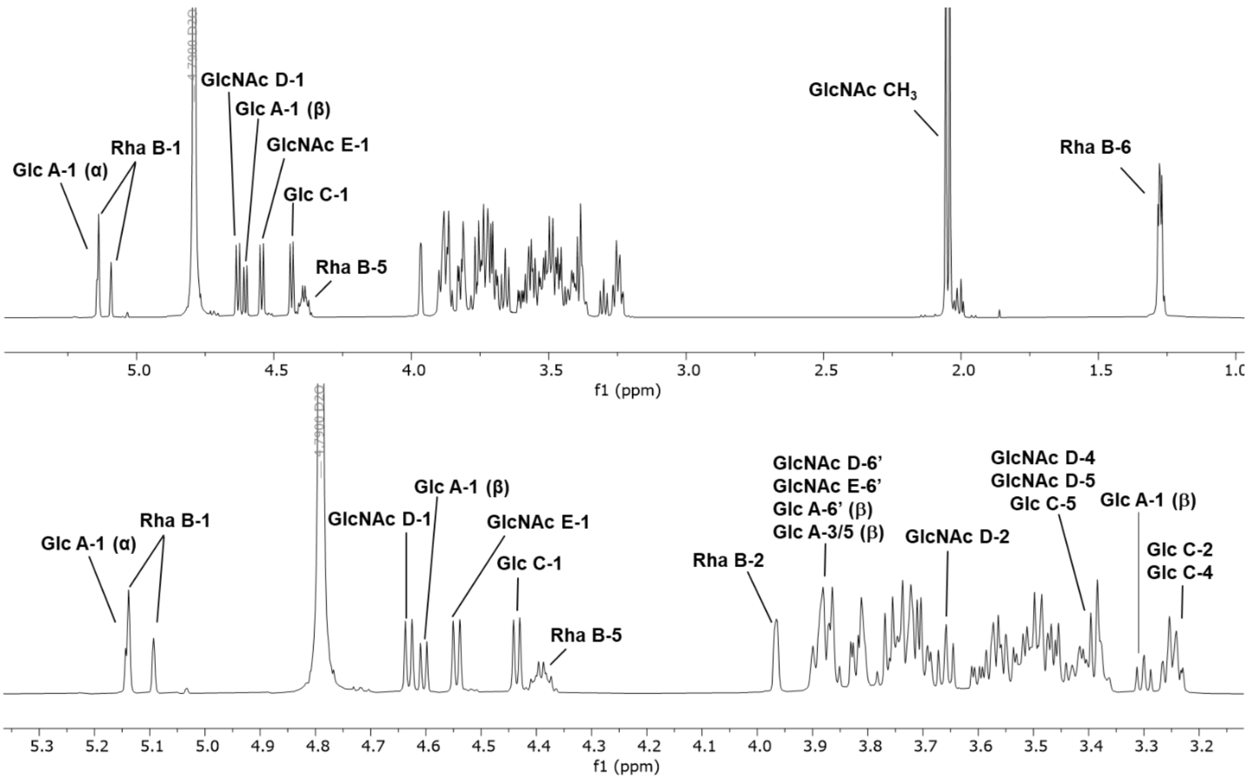

Figure S26 ^{1}H NMR (700 MHz, D_2O) of **5mer-III-NN** with assignments.

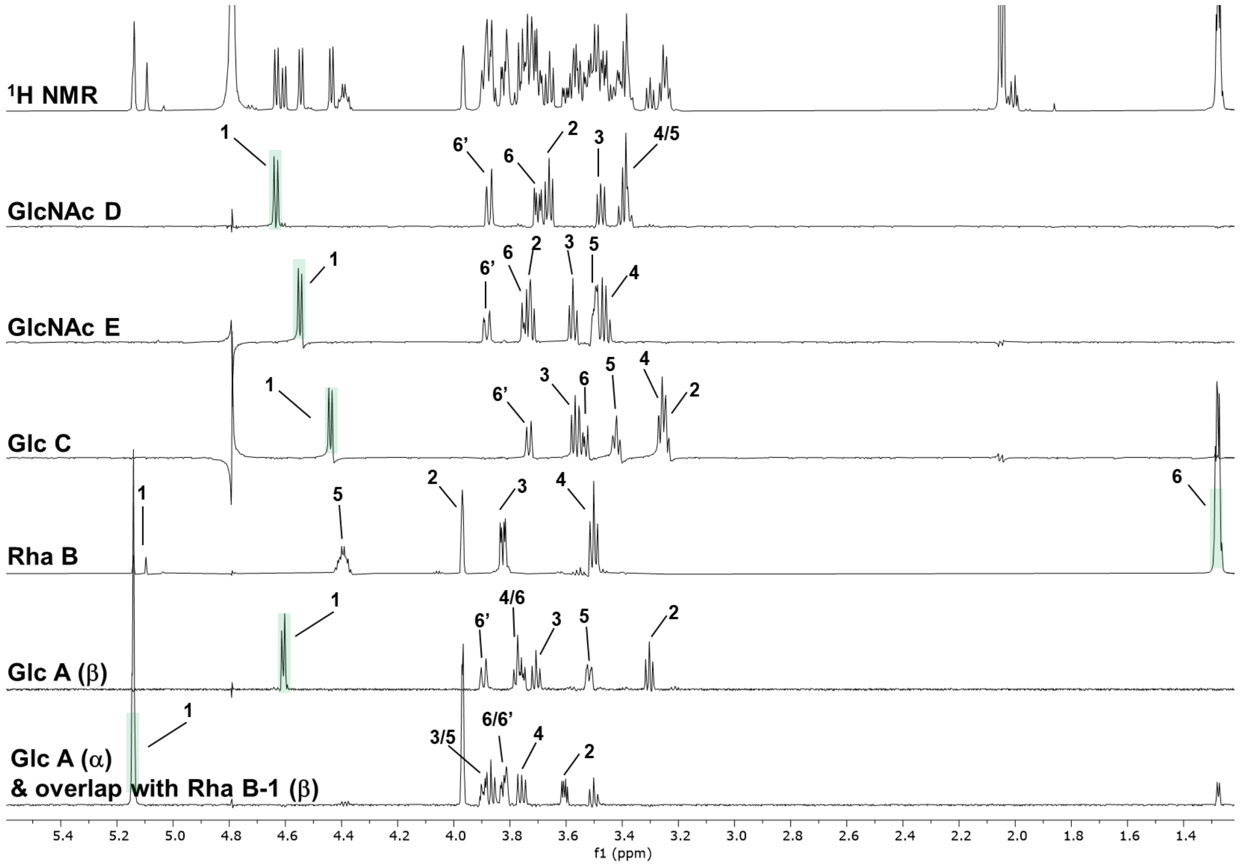

Figure S27 1D TOCSY (700 MHz, d9 200 ms, D_2O) of **5mer-III-NN** with assignments. Resonances chosen for selective excitation are highlighted in green.

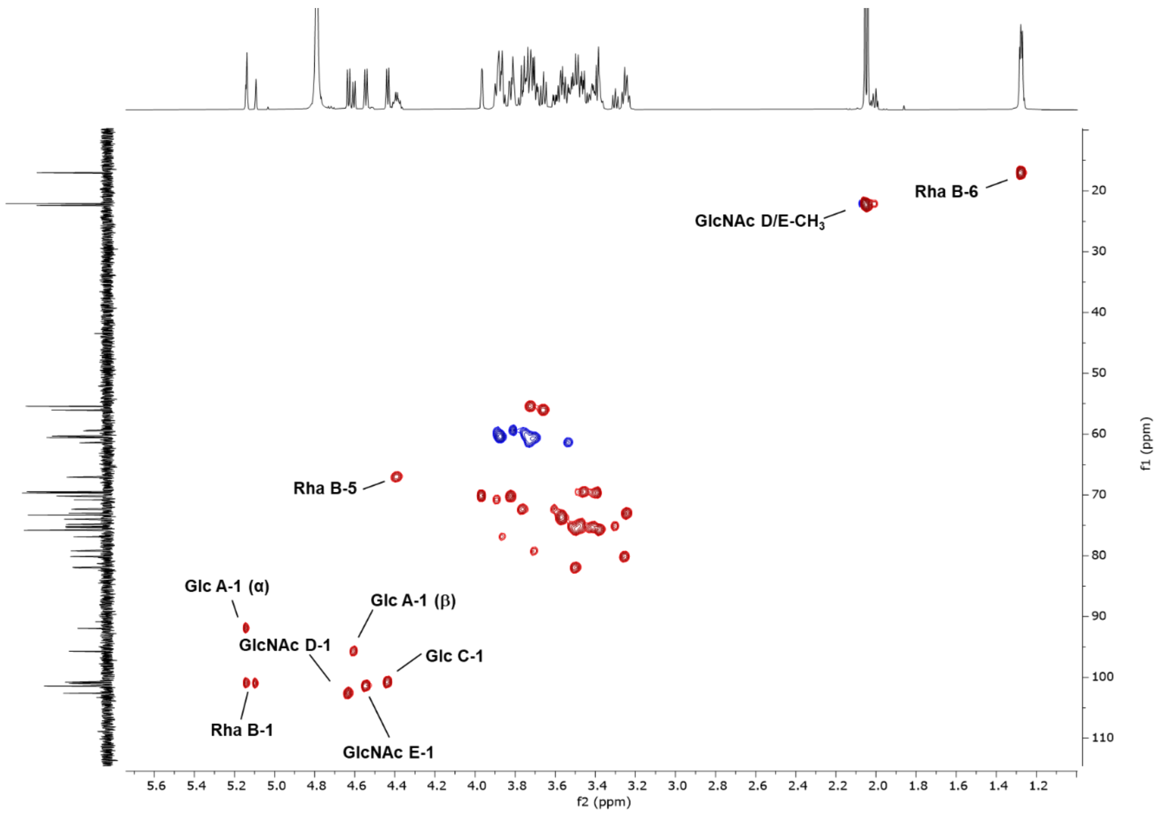

Figure S28 HSQC NMR (D_2O) of **5mer-III-NN** with assignments.

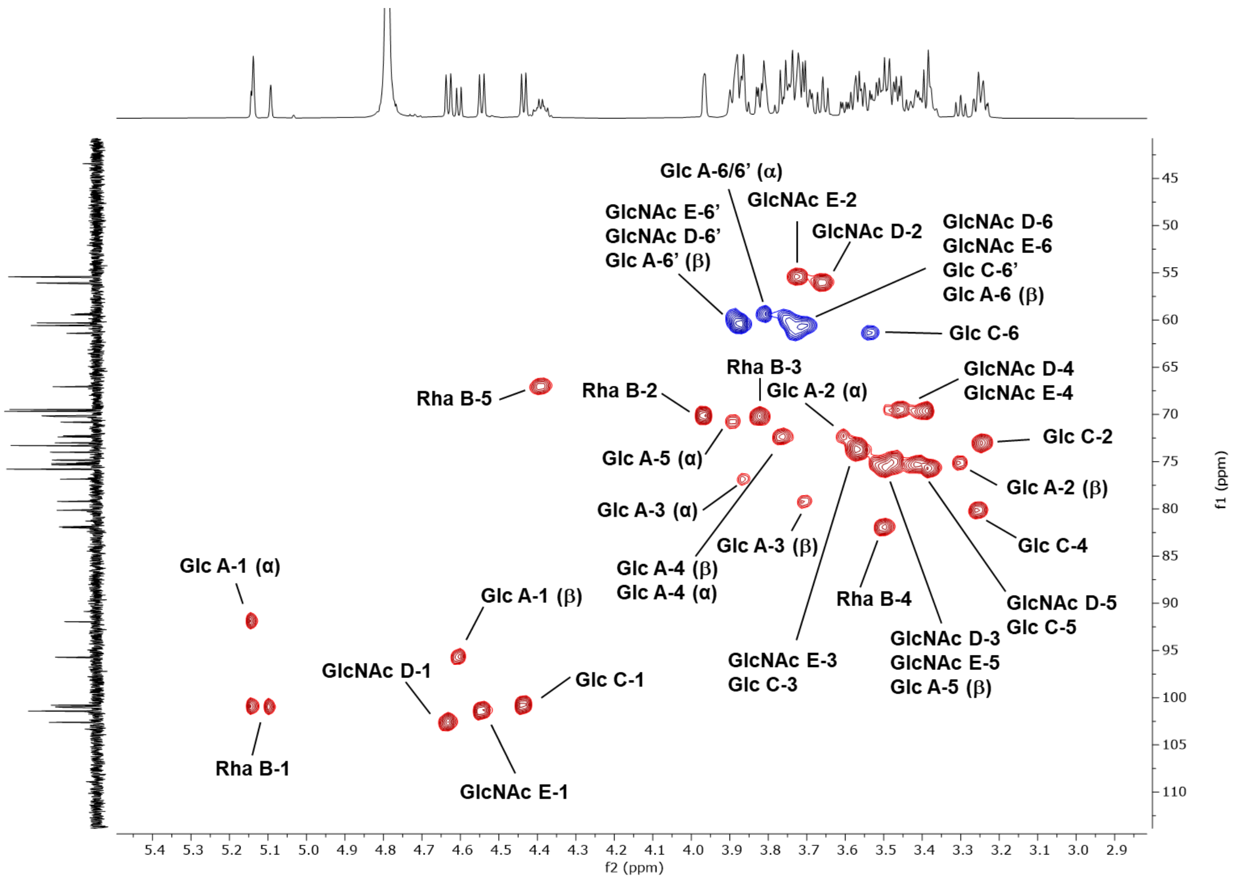

Figure S29 Excerpt of HSQC NMR (D_2O) of **5mer-III-NN** with assignments.

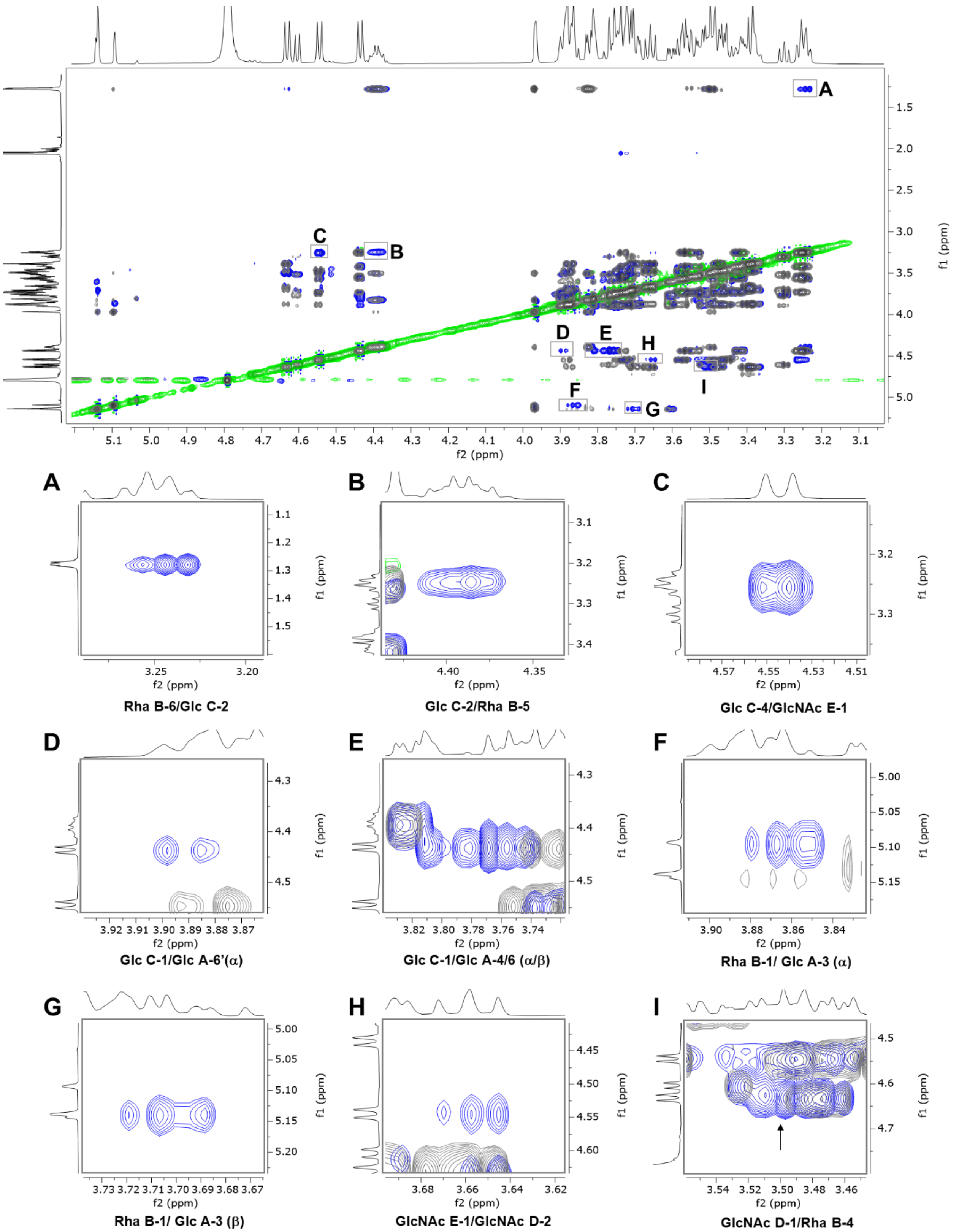

Figure S30 Overimposed 2D ROESY (green-blue, 700 MHz, p15 300 ms, 293 K, D_2O) with assignments and 2D TOCSY spectrum (gray, 700 MHz, d9 150 ms, D_2O) of **5mer-III-NN.**

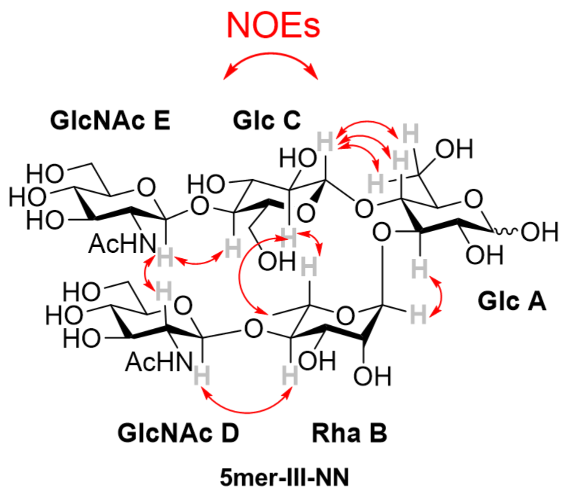

Figure S31 All experimentally observed NOEs (red arrows).

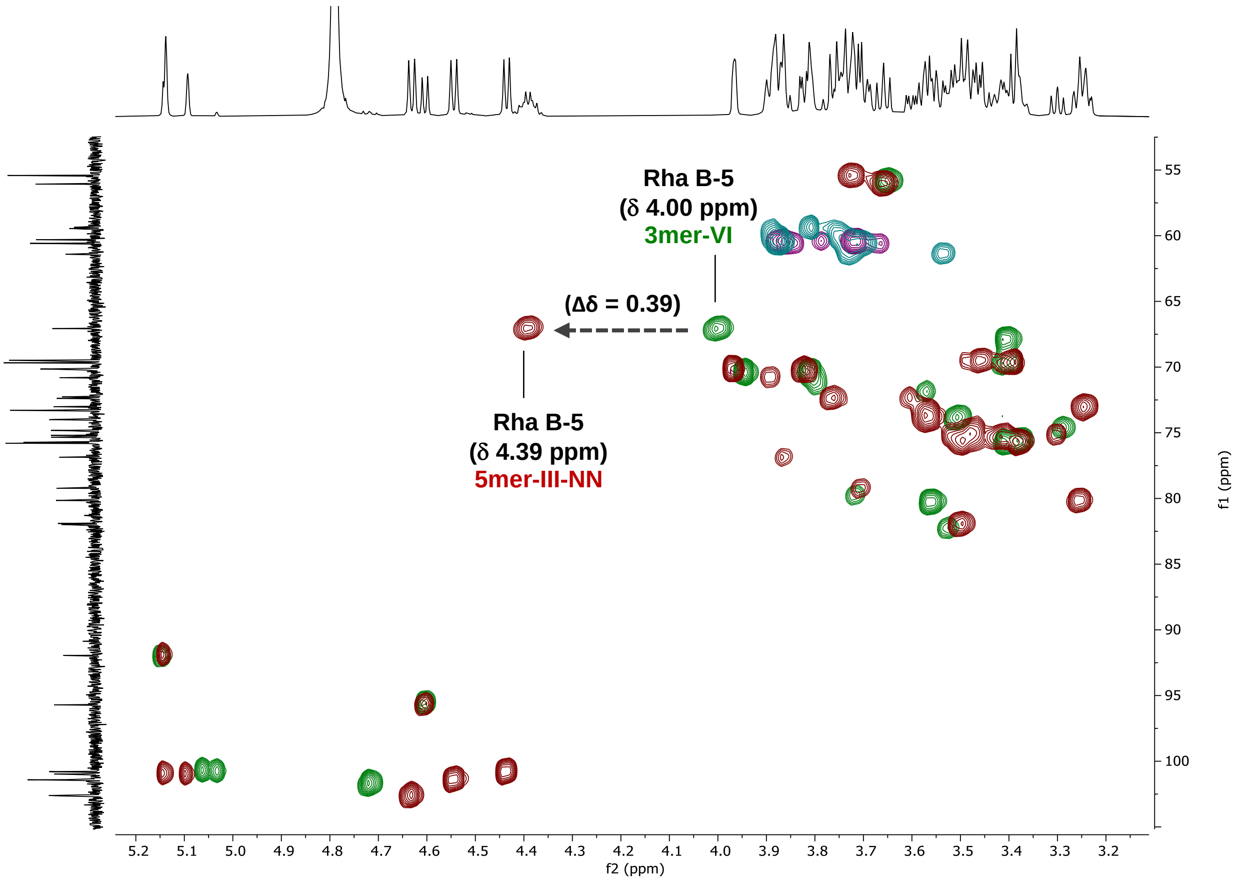

Figure S32 Overimposed 2D HSQC **3mer-VI** (green-purple) and **5mer-III-NN** (red-cyan) showing a downfield shift of Rha B-5 from 4.00 to 4.39 ppm, indicating the presence of non-conventional H-bond in **5mer-III-NN**.

4.1.1 STEP-t-ROESY inter-residue distance estimation

To determine the distance between turn residues Rha B and Glc C, NOEs from Rha B-5 were estimated. As this proton shows overlap in all the structures, a STEP-t-ROESY strategy was employed. Isotropic mixing from Rha B-6 was optimized to obtain the highest signal at Rha B-5 at the possible lower mixing time, which was 40 ms. Selective irradiation of Rha B-6 followed by TOCSY transfer of magnetization to Rha B-5 and then t-ROESY from Rha B-5 permitted us to measure the intra-residue Rha B-5/Rha B-3 (as reference) and the inter-residue Rha B-5/Glc C-2 ROEs. To determine the distance between strand residues Glc D and Glc E, NOEs from Glc D-2 were measured. Selective irradiation of Glc D-1 followed by TOCSY transfer of magnetization to Glc D-2 and then t-ROESY from Glc D-2 permitted us to measure the intra-residue Glc D-2/Glc D-4 (as reference) and the inter-residue Glc D-2/Glc E-1 ROEs. The intra-residue distance calculations for the corresponding OMe derivatives obtained with Maestro Software:

1. Minimization with MacroModel (OPLS4 force field, water)
2. Energy Optimization with Jaguar (B3LYP-D3_6-31G**, DFT, solvent=water_PBF)

	r ref B5-B3	r ref D2-D4
**L-Rha α-OMe**		2.5
**GlcNAc-β-OMe**	2.5	

The equation used to estimate the inter-residue distance is given below; where r_{ij} is estimated distance, r_{ref} is reference distance, V_{ref} is integral of NOE peak between reference proton pair and V_{ij} is integral of NOE peak between target proton pair.

$$r_{ij} = r_{ref} \times \left(\frac{V_{ref}}{V_{ij}}\right)^{1/6}$$

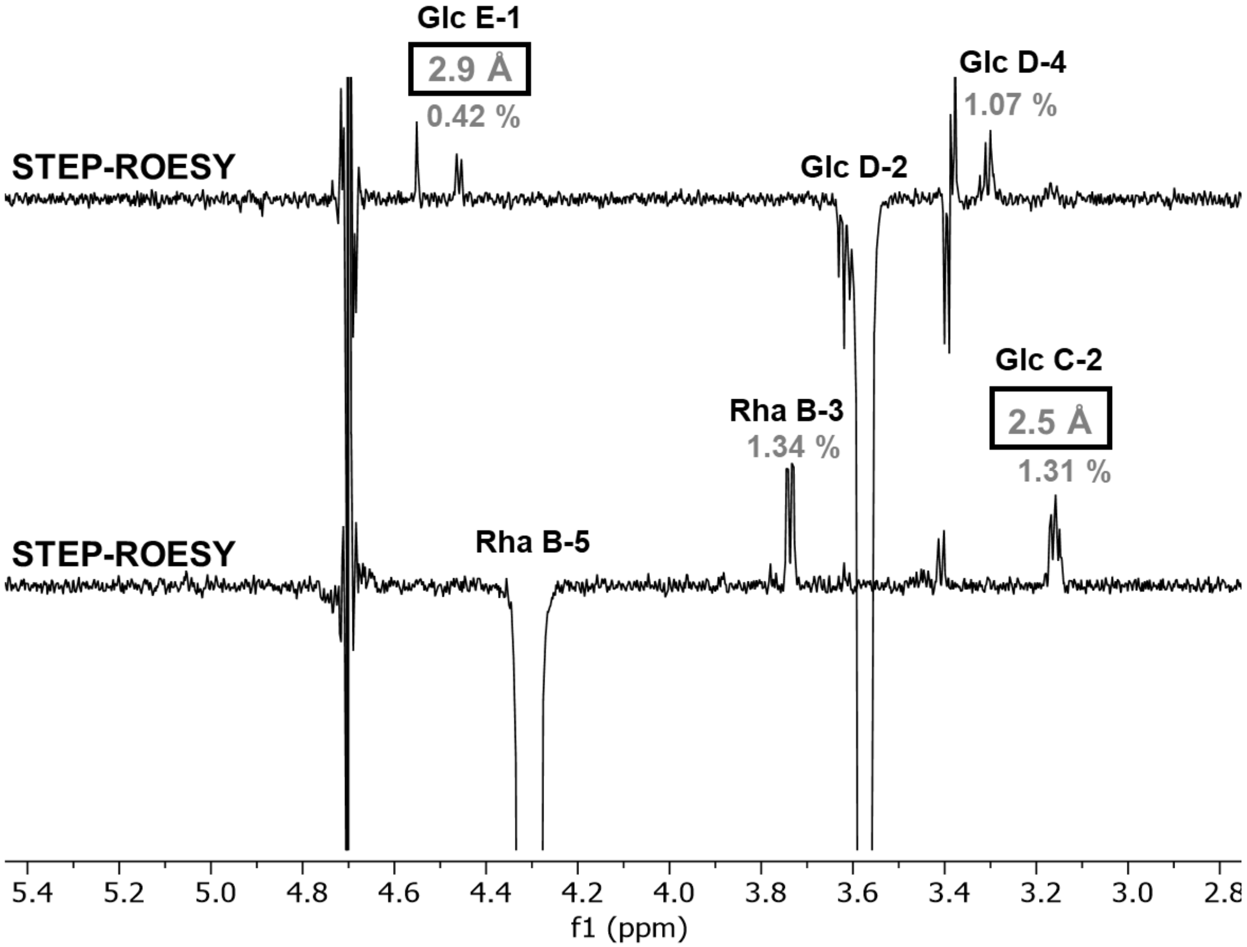

Figure S33 STEP-t-ROESY (200 ms) experiments to estimate the interstrand Glc C-2/Rha B-5 and Glc D-2/Glc E-1 NOE distance for **5mer-III-NN** (293 K, D_2O, 800 MHz). Isotropic mixing from Rha B-6 (or Glc D-2) was optimized to obtain the highest signal at Rha B-5 at the possible lower mixing time, which was 40 ms. Selective irradiation of Rha B-6 followed by TOCSY transfer of magnetization to Rha B-5 and then t-ROESY from Rha B-5 permitted us to measure the intra-residue Rha B-5/Rha B-3 (as reference) and the inter-residue Rha B-5/Glc C-2 NOEs.

4.2 H-bond study in 5mer-III-NN

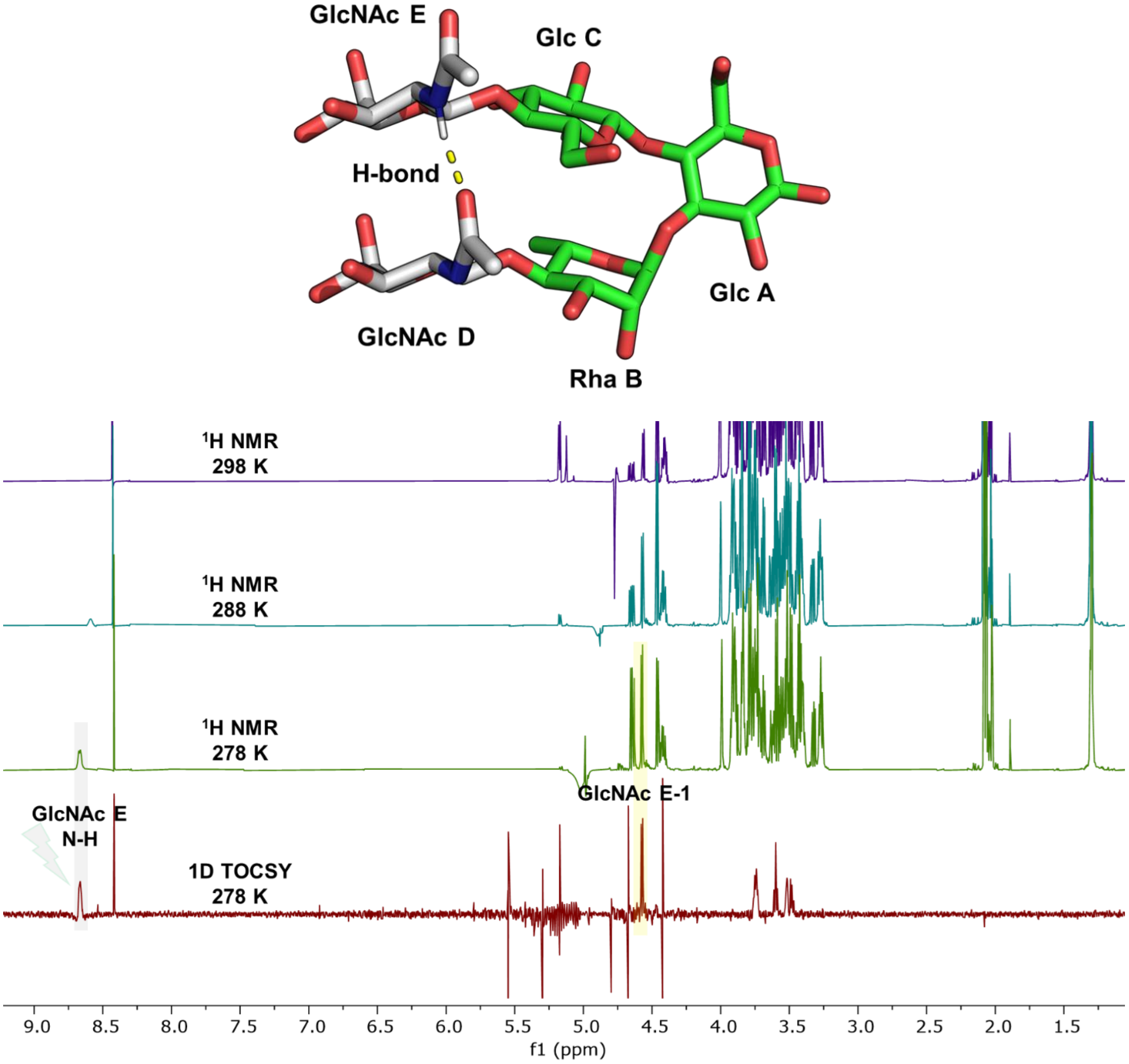

Figure S34 ^{1}H NMR spectra of **5mer-III-NN** at different temperature in D_2O:H_2O (1:9) showing a sharp N-H peak arising at 278 K (highlighted in gray). This signal belongs to the GlcNAc E residue as confirmed by 1D TOCSY NMR (in red). Resonances chosen for selective excitation are highlighted in light gray.

4.3 H-bond study of 9mer-III-NN

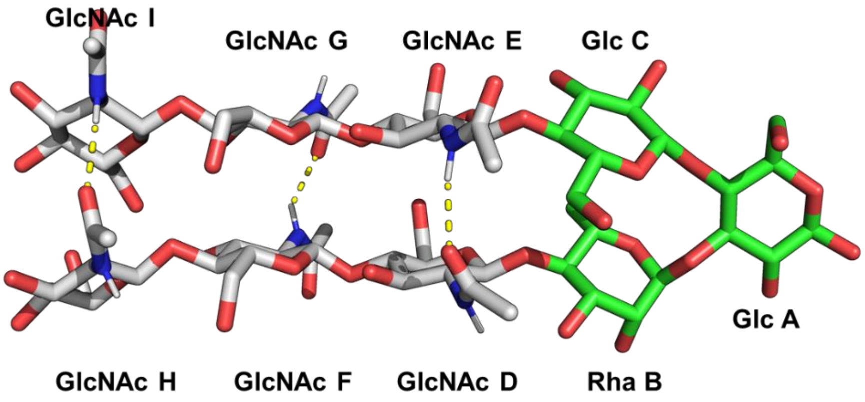

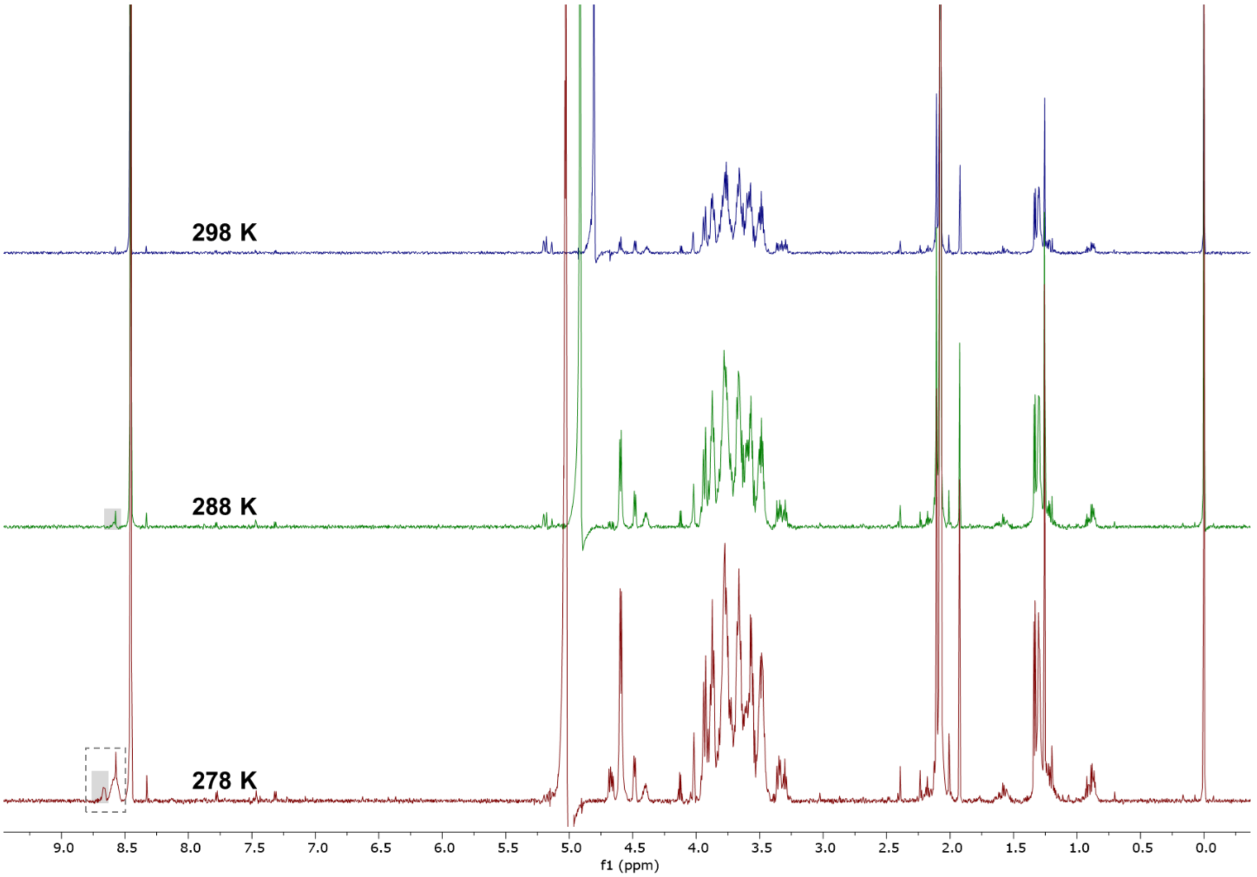

Figure S35 ^{1}H NMR spectra of **9mer-III-NN** at different temperature in D_2O:H_2O (1:9) showing N-H peaks arising at 278 K are highlighted in gray dash box. The more deshielded N-H signal highlighted with gray shading is assigned to GlcNAc E residue. This was subsequently confirmed by stacked 2D NOESY spectra of **5mer-III-NN** and **9mer-III-NN** (Figure S38).

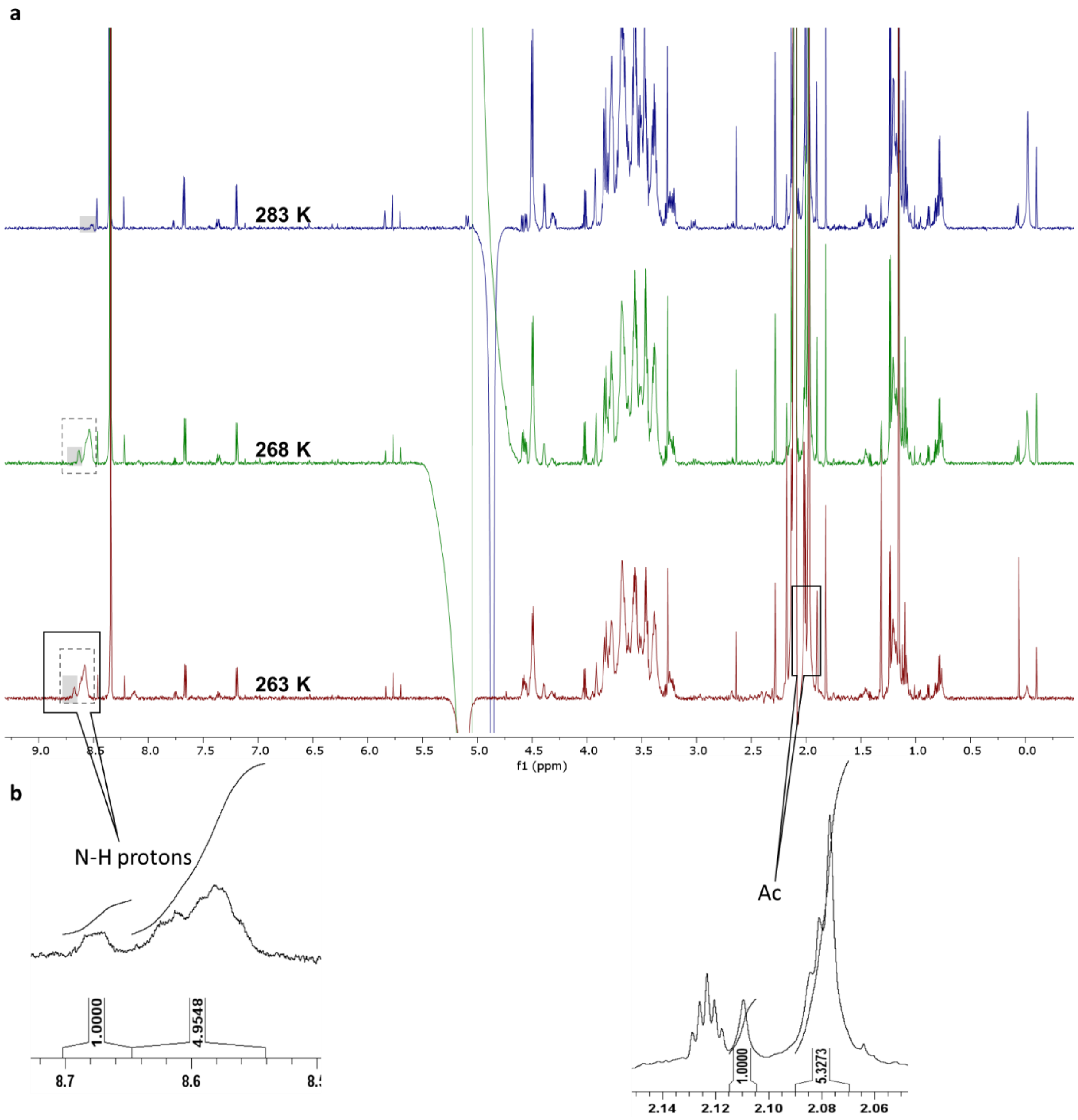

Figure S36 a) ^{1}H NMR spectra of **9mer-III-NN** at different temperature in C_3D_6O:H_2O (1:9) showing N-H peaks at lower temperatures (268 K and 263 K) highlighted in gray dash box. The more deshielded N-H signal highlighted with gray shading is assigned to GlcNAc E residue. This was subsequently confirmed by stacked 2D NOESY spectra of **5mer-III-NN** and **9mer-III-NN** (Figure S38). **b)** N-H proton and Ac group integral showing only one N-H proton and Ac are differentiated.

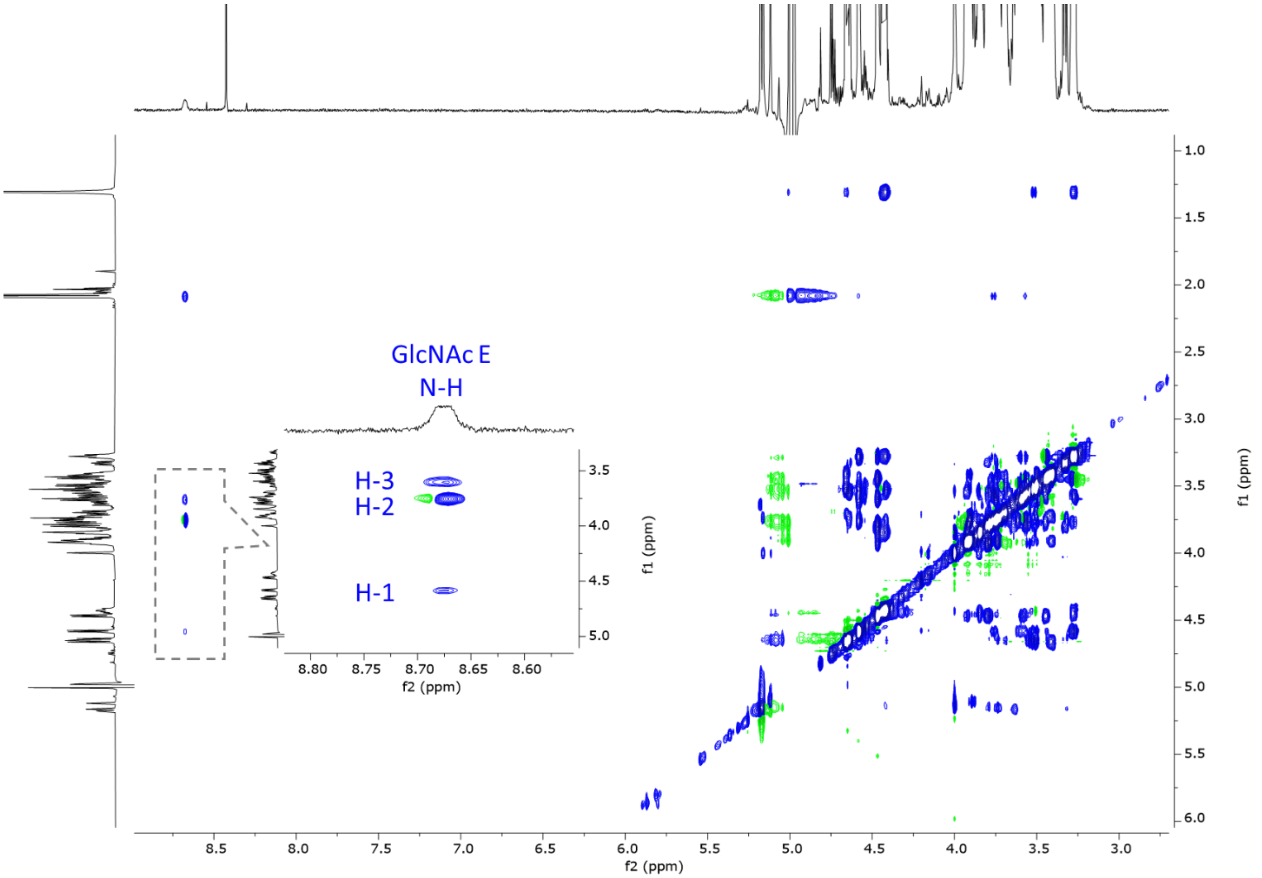

Figure S37 2D NOESY spectrum (green-blue, 800 MHz, d8 600 ms, 278 K, D_2O/H_2O (1:9)) of **5mer-III-NN**. Highlighted are the protons correlating with amide proton of GlcNAc E residue.

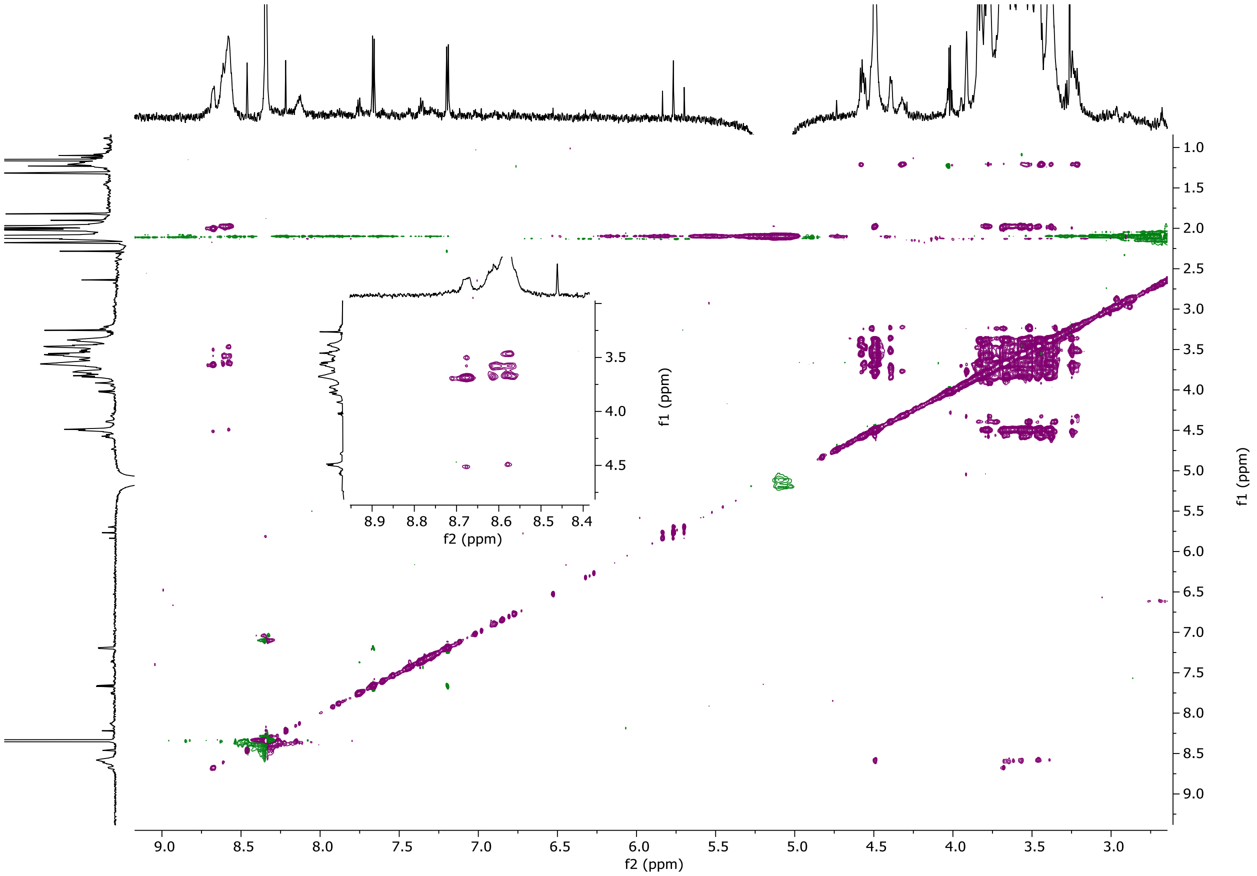

Figure S38 2D NOESY spectrum (green-blue, 800 MHz, d8 400 ms, 268 K, C_3D_6O/H_2O (1:9)) of **9mer-III-NN**. Highlighted are the protons correlating with amide protons of **9mer-III-NN**.

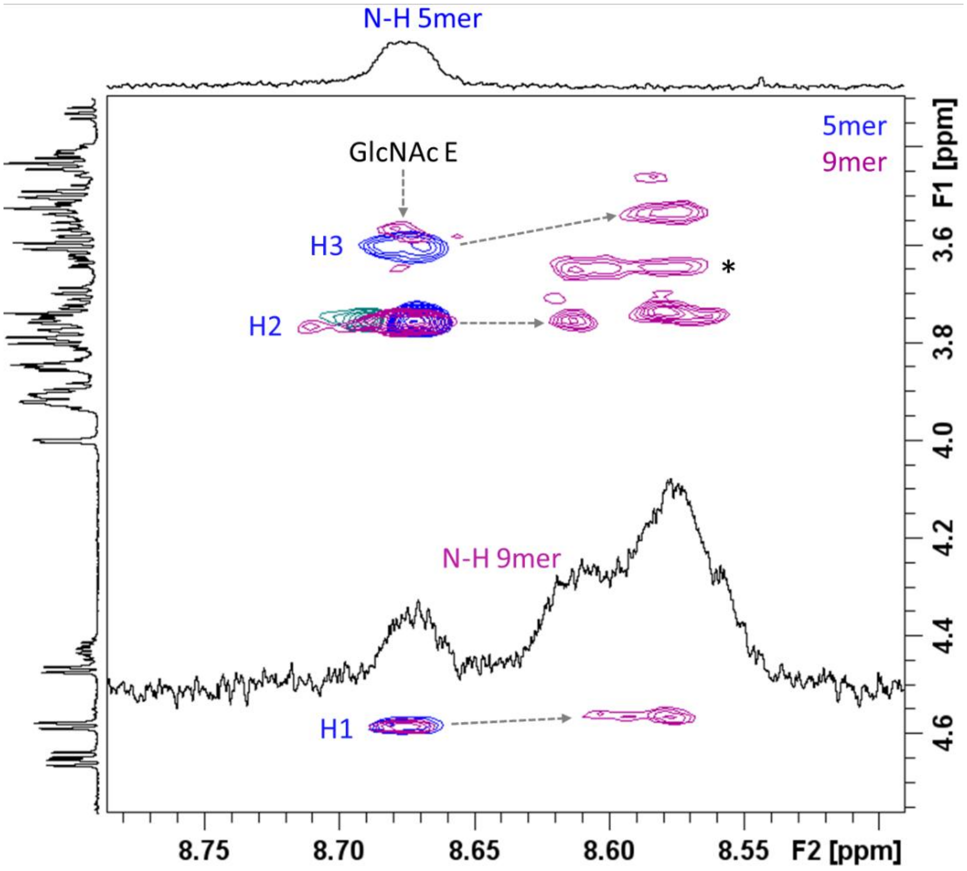

Figure S39 Overimposed 2D NOESY spectra of **5mer-III-NN (Figure S37)** and **9mer-III-NN (Figure S38)**, showing the more deshielded N-H proton in **9mer-III-NN** belongs to GlcNAc E residue as in **5mer-III-NN**. The **9mer-III-NN** spectrum was shifted to adjust to **5mer-III-NN**.

4.4 Temperature coefficient comparison for amide protons in 5mer-III-NN & 9mer-III-NN

The temperature coefficients[16] for the amido N-H protons were calculated to provide insight into hydrogen bond stability and solvent exposure in the two molecules studied.

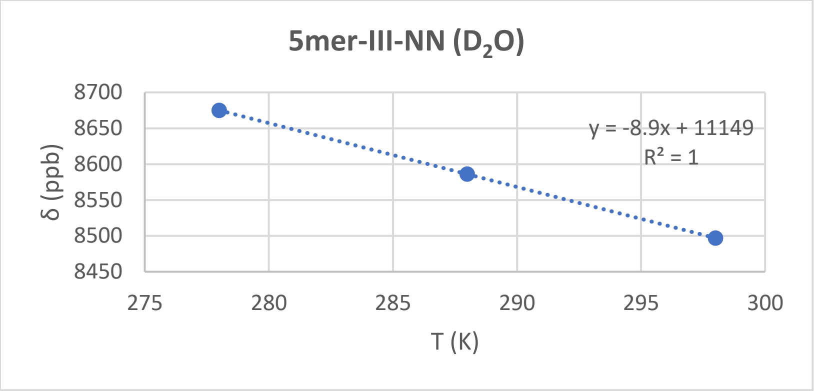

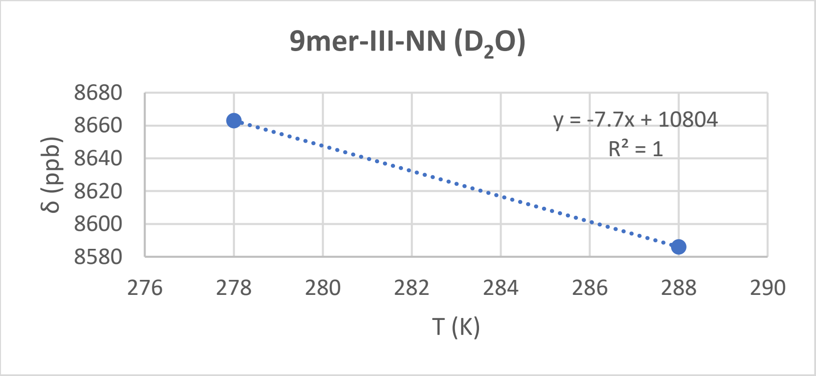

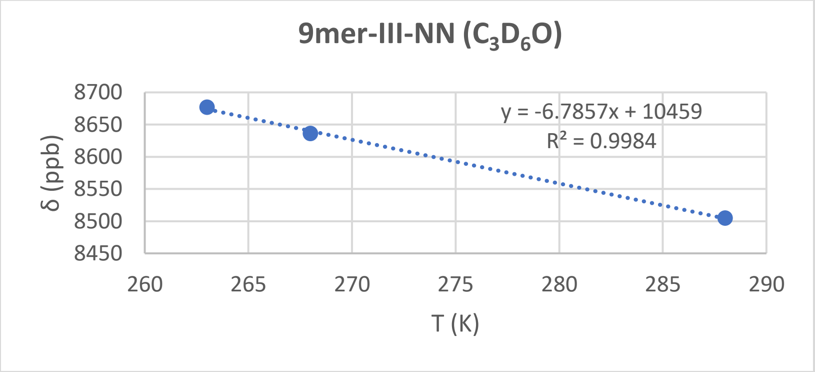

Structures and solvent used	Temperature coefficients (ppb/K)
**5mer-III-NN** (H_2O/D_2O)	-8.90
**9mer-III-NN** (H_2O/D_2O)	-7.70
**9mer-III-NN** (H_2O/C_3D_6O)	-6.79

Table S1 Temperature coefficients values for **5mer-III-NN** and **9mer-III-NN** calculated by NMR.

4.5 Proton and carbon chemical shift values of 5mer-III-NN

Residue	Proton labels	Chemical shifts δ (ppm)	
		^{1}H	^{13}C
Glc A (α)	Glc A-1 (α)	5.14	91.93
	Glc A-2 (α)	3.60	72.26
	Glc A-3 (α)	3.87	76.93
	Glc A-4 (α)	3.76	72.42
	Glc A-5 (α)	3.90	70.81
	Glc A-6 (α)	3.82	59.36
	Glc A-6' (α)	3.83	59.36
Glc A (β)	Glc A-1 (β)	4.60	95.64
	Glc A-2 (β)	3.31	75.16
	Glc A-3 (β)	3.71	79.19
	Glc A-4 (β)	3.77	72.42
	Glc A-5 (β)	3.52	75.64
	Glc A-6 (β)	3.75	60.33
	Glc A-6' (β)	3.89	60.49
Rha B	Rha B-1	5.14, 5.10	100.96, 100.96
	Rha B-2	3.96	70.16
	Rha B-3	3.82	70.16
	Rha B-4	3.50	81.93
	Rha B-5	4.39	67.10
	Rha B-6	1.28	16.93
Glc C	Glc C-1	4.45	100.80
	Glc C-2	3.25	73.07
	Glc C-3	3.57	73.71
	Glc C-4	3.26	81.16
	Glc C-5	3.42	75.64
	Glc C-6	3.54	61.29
	Glc C-6'	3.74	60.33
GlcNAc D	GlcNAc D-1	4.63	102.57
	GlcNAc D-2	3.67	55.97
	GlcNAc D-3	3.48	75.16
	GlcNAc D-4	3.39	69.68
	GlcNAc D-5	3.39	75.32
	GlcNAc D-6	3.71	60.69
	GlcNAc D-6'	3.86	60.49
	GlcNAc D-CH_3	2.08	22.27

GlcNAc E	GlcNAc E-1	4.54	101.28
	GlcNAc E-2	3.73	55.33
	GlcNAc E-3	3.57	73.71
	GlcNAc E-4	3.46	69.52
	GlcNAc E-5	3.50	75.64
	GlcNAc E-6	3.75	60.33
	GlcNAc E-6’	3.88	60.49
	GlcNAc E-CH_3	2.08	22.27

Table S2 Chemical shifts assigned for each proton of **5mer-III-NN**.

5 SAXS Analysis of 9mer-III-NN

5.1 General materials and methods

X-ray scattering experiments were performed at the BM26 beamline of the European Synchrotron Radiation Facility (ESRF). Sample of **9mer-III-NN** with concentrations of 0.25 and 1.0 % wt at 25 ^{o}C were sealed in glass capillaries and mounted on a motorized sample changer. They were exposed to monochromatic X-rays of 12 keV (λ = 1.033 Å). The scattering intensity was measured using two-dimensional pixel detectors (Pilatus1M, Dectris). The data processing was performed using pyFAI software.[17] Intensity and Rg analysis were done using Gnuplot software.

5.2 SAXS calculation

Explicit-solvent SAXS calculations were performed based on a modified version[18, 19] of GROMACS 2022.2 (GROMACS-SWAXS).[20] Details of explicit-solvent SAXS calculations are presented in a previous publication.[21] Here, in this modified GROMACS code, the solvent molecules in the solvation layer were considered in the SAXS intensity calculation, in contrast to the common approach where only the solute molecules are used for the SAXS intensity simulations. This explicit solvent model can provide realistic simulated intensities, as the density fluctuation of water molecules around the solute certainly contributes to the overall SAXS intensities. A spatial envelope was built around the hairpin at a distance of 0.7 nm. The water subtraction was carried out using > 1000 simulation frames of the pure-water simulation box. The atomic form factors were approximated by

$$f_j(q) = \sum_{k=1}^{4} c + a_k e^{-b_k\left(\frac{q}{4\pi}\right)^2},$$

where the values a_k, b_k and c are the Cromer-Mann parameters. The orientational average was carried out using 500 q-vectors for each absolute value of q, and the solvent electron density was corrected to the experimental value of 334 e/nm^3.

5.3 **Experimental Rg calculation**

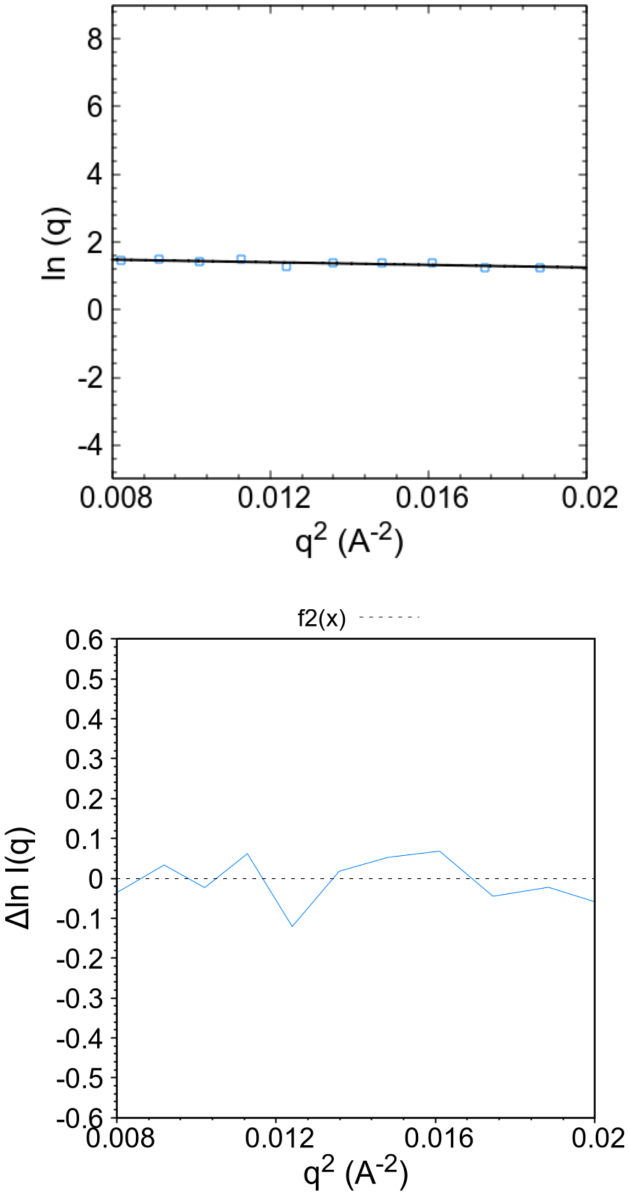

Figure S40 Guinier plots and residuals of **9mer-III-NN.**

9mer-III-NN

	**Value**	**Error**
**m**	-19.4	5.79
**n**	1.63	0.08
**R_g (Å)**	7.6	0.07
**RMSE**	0.030	

Table S3 Curve fitting of natural logarithm of I(q) vs. q^2 plots of Guinier law-SAXS measurements of **9mer-III-NN.**

6 Triggered assembly of 9mer-III-NN

6.1 Crystallization method

The following procedure was adapted from reported work.[22]

4 mL of Acetone was preheated to its boiling point in a Falcon tube for 20 minutes. Separately, 10 µL of the glycan solution (0.05% w/v) was preheated at 70 °C for 5 min and subsequently placed into the Falcon tube so that acetone could enter the Eppendorf vial exclusively via vapor diffusion. Vapor diffusion was maintained at 70 °C for 2 h, followed by gradual cooling to room temperature. The resulting crystals were stored in 60 µL of acetone.

6.2 Transmission electron microscopy imaging

Transmission electron microscopy (TEM) was performed using a JEOL JEM F200 (Jeol, Japan) (S)TEM equipped with a field emission gun and a TVIPS TemCam-F126 (2k x 2k) camera. The microscope was operated at 80 kV and a condenser aperture with a diameter of 200 µm was used. For specimen preparation, drops (2 µL) of solvent suspensions of samples were deposited on glow-discharged carbon-coated copper grids (Micro to Nano BV, Netherlands). The grid was left to dry for 1h before imaging.

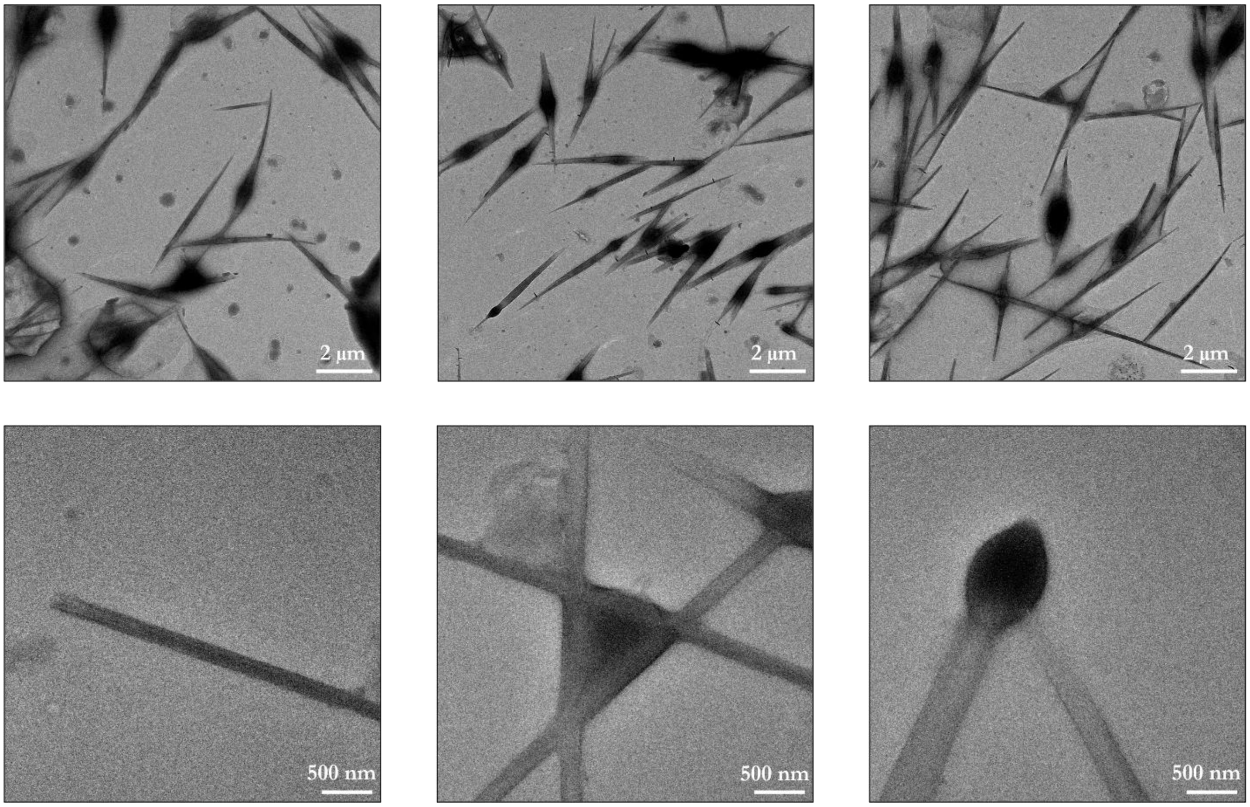

Figure S41 Representative TEM images of crystallized **9mer-III-NN** obtained through crystallization procedure described in Section 5.

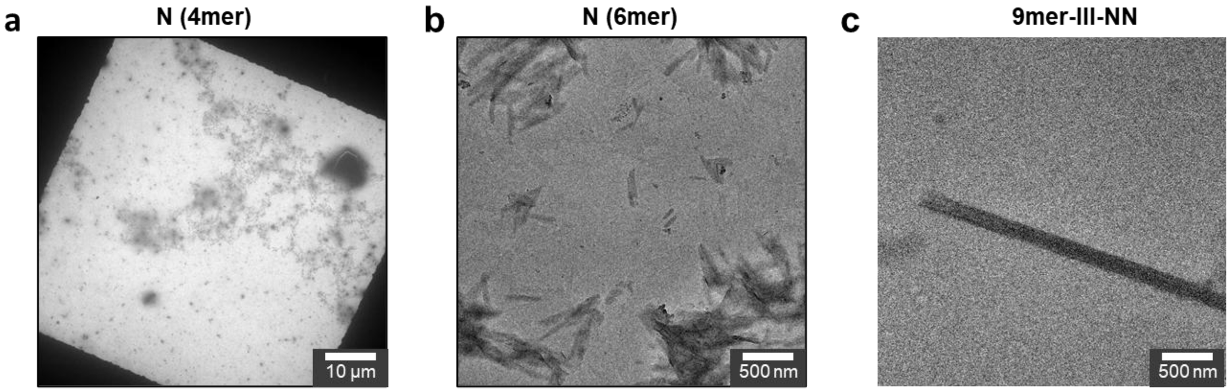

Figure S42 TEM images of previously studied linear sequences of chitin[23] **a) N (4mer)** showing no crystalline aggregation **b) N (6mer)** assembled into small crystallites, and crystallized **c) 9mer-III-NN** studied in this work.

6.3 ED analysis

Drops (2 μL) of solvent suspensions of samples was deposited on a glow-discharged carbon-coated copper grid. TEM and ED experiments were performed using a JEM-2100Plus and JEM F200cryo transmission electron microscopes (JEOL ltd., Japan) operated at an accelerating voltage of 200 kV. A Merlin 2D hybrid pixel detector (Quantum Detectors) with a pixel size of 55 x 55 μm2 was used to acquire a series of electron diffraction patterns as the sample continuously rotated within the microscope. The tilt-series ED data were processed using *PETS2*[24] software.

7 References

(1) Kirschner, K. N.; Yongye, A. B.; Tschampel, S. M.; González-Outeiriño, J.; Daniels, C. R.; Foley, B. L.; Woods, R. J. GLYCAM06: a generalizable biomolecular force field. Carbohydrates. *J. Comput. Chem.* **2008**, *29* (4), 622–655. https://doi.org/10.1002/jcc.20820.

(2) Mahoney, M. W.; Jorgensen, W. L. A five-site model for liquid water and the reproduction of the density anomaly by rigid, nonpolarizable potential functions. *J. Chem. Phys.* **2000**, *112* (20), 8910–8922. https://doi.org/10.1063/1.481505.

(3) Darden, T.; York, D.; Pedersen, L. Particle mesh Ewald: An N·log(N) method for Ewald sums in large systems. *J. Chem. Phys.* **1993**, *98* (12), 10089–10092. https://doi.org/10.1063/1.464397.

(4) Van Der Spoel, D.; Lindahl, E.; Hess, B.; Groenhof, G.; Mark, A. E.; Berendsen, H. J. C. GROMACS: Fast, flexible, and free. *J. Comput. Chem.* **2005**, *26* (16), 1701–1718. https://doi.org/10.1002/jcc.20291.

(5) Evans, D. J.; Holian, B. L. The Nose–Hoover thermostat. *J. Chem. Phys.* **1985**, *83* (8), 4069–4074. https://doi.org/10.1063/1.449071.

(6) Parrinello, M.; Rahman, A. Polymorphic transitions in single crystals: A new molecular dynamics method. *J. Appl. Phys.* **1981**, *52* (12), 7182–7190. https://doi.org/10.1063/1.328693.

(7) Ives, C. M.; Singh, O.; D'Andrea, S.; Fogarty, C. A.; Harbison, A. M.; Satheesan, A.; Tropea, B.; Fadda, E. Restoring protein glycosylation with GlycoShape. *Nat. Methods* **2024**, *21* (11), 2117–2127. https://doi.org/10.1038/s41592-024-02464-7.

(8) Gardner, K. H.; Blackwell, J. Refinement of the structure of beta-chitin. *Biopolym.* **1975**, *14* (8), 1581–1595. https://doi.org/10.1002/bip.1975.360140804.

(9) Carlstrom, D. The crystal structure of alpha-chitin (poly-N-acetyl-D-glucosamine). *J. Biophys. Biochem. Cytol.* **1957**, *3* (5), 669–683. https://doi.org/10.1083/jcb.3.5.669.

(10) Eller, S.; Collot, M.; Yin, J.; Hahm, H. S.; Seeberger, P. H. Automated solid-phase synthesis of chondroitin sulfate glycosaminoglycans. *Angew. Chem. Int. Ed.* **2013**, *52* (22), 5858–5861. https://doi.org/10.1002/anie.201210132.

(11) Fittolani, G.; Tyrikos-Ergas, T.; Poveda, A.; Yu, Y.; Yadav, N.; Seeberger, P. H.; Jiménez-Barbero, J.; Delbianco, M. Synthesis of a glycan hairpin. *Nature Chemistry* **2023**, *15* (10), 1461–1469. https://doi.org/10.1038/s41557-023-01255-5.

(12) Le Mai Hoang, K.; Pardo-Vargas, A.; Zhu, Y.; Yu, Y.; Loria, M.; Delbianco, M.; Seeberger, P. H. Traceless Photolabile Linker Expedites the Chemical Synthesis of Complex Oligosaccharides by Automated Glycan Assembly. *J. Am. Chem. Soc.* **2019**, *141* (22), 9079–9086. https://doi.org/10.1021/jacs.9b03769.

(13) Gude, M.; Ryf, J.; White, P. D. An accurate method for the quantitation of Fmoc-derivatized solid phase supports. *Lett. Pept. Sci.* **2002**, *9* (4), 203–206. https://doi.org/10.1023/A:1024148619149.

(14) Hurevich, M.; Kandasamy, J.; Ponnappa, B. M.; Collot, M.; Kopetzki, D.; McQuade, D. T.; Seeberger, P. H. Continuous photochemical cleavage of linkers for solid-phase synthesis. *Org. Lett.* **2014**, *16* (6), 1794–1797. https://doi.org/10.1021/ol500530q.

(15) Yu, Y.; Tyrikos-Ergas, T.; Zhu, Y.; Fittolani, G.; Bordoni, V.; Singhal, A.; Fair, R. J.; Grafmüller, A.; Seeberger, P. H.; Delbianco, M. Systematic Hydrogen-Bond Manipulations To Establish Polysaccharide Structure–Property Correlations. *Angew. Chem. Int. Ed.* **2019**, *58* (37), 13127–13132. https://doi.org/10.1002/anie.201906577.

(16) Cierpicki, T.; Otlewski, J. Amide proton temperature coefficients as hydrogen bond indicators in proteins. *J. Biomol. NMR* **2001**, *21* (3), 249–261. https://doi.org/10.1023/a:1012911329730.

(17) Kieffer, J.; Karkoulis, D. PyFAI, a versatile library for azimuthal regrouping. *J. Phys.: Conf. Ser.* **2013**, *425* (20), 202012. https://dx.doi.org/10.1088/1742-6596/425/20/202012.

(18) Chen, P. C.; Hub, J. S. Validating solution ensembles from molecular dynamics simulation by wide-angle X-ray scattering data. *Biophys J* **2014**, *107* (2), 435–447. https://doi.org/10.1016/j.bpj.2014.06.006.

(19) Hub, J. S. Interpreting solution X-ray scattering data using molecular simulations. *Current Opinion in Structural Biology* **2018**, *49*, 18–26. https://doi.org/10.1016/j.sbi.2017.11.002.

(20) Knight, C. J.; Hub, J. S. WAXSiS: a web server for the calculation of SAXS/WAXS curves based on explicit-solvent molecular dynamics. *Nucleic Acids Res* **2015**, *43* (W1), W225–230. https://doi.org/10.1093/nar/gkv309.

(21) Chatzimagas, L.; Hub, J. S. Chapter Fifteen - Predicting solution scattering patterns with explicit-solvent molecular simulations. In *Methods in Enzymology*, Tainer, J. A. Ed.; Vol. 677; Academic Press, 2022; pp 433–456.

(22) Montesanti, N.; Lancelon-Pin, C.; Potocki-Veronese, G.; Buléon, A.; Putaux, J.-L. A-amylose single crystals: influence of amylose concentration, crystallization temperature and surface induction on the crystal morphology. *Cellulose* **2023**, *30* (13), 8459–8473. https://doi.org/10.1007/s10570-023-05387-2.

(23) Djalali, S.; Jing, Y.; Ogawa, Y.; Delbianco, M. Synthetic chitin oligosaccharide nanocrystals and their higher-order assemblies. *Chemical Science* **2025**, *16* (3), 1390–1395. https://doi.org/10.1039/D4SC07549H.

(24) Palatinus, L.; Brazda, P.; Jelinek, M.; Hrda, J.; Steciuk, G.; Klementova, M. Specifics of the data processing of precession electron diffraction tomography data and their implementation in the program PETS2.0. *Acta Crystallographica Section B* **2019**, *75* (4), 512–522. https://doi.org/doi:10.1107/S2052520619007534.
